# Reprogramming the enduracidin NRPS assembly line via thioesterase domain relocation

**DOI:** 10.1016/j.synbio.2026.05.013

**Published:** 2026-06-24

**Authors:** Yanan Sun, Guibin Tu, Xiaocan Sun, Hanchao Zhang, Haikuan Wang, Fufeng Liu, Fuping Lu, Huitu Zhang

**Affiliations:** Key Laboratory of Industrial Fermentation Microbiology, College of Biotechnology, Tianjin University of Science & Technology, Tianjin, 300457, PR China

**Keywords:** Nonribosomal peptide synthetase (NRPS), Thioesterase (TE) domain, Enduracidin, Macrocyclic peptides

## Abstract

The rational engineering of multi-megadalton nonribosomal peptide synthetase (NRPS) assembly lines remains a formidable challenge due to their immense size and complexity. Conventional module deletion often disrupts the native cyclization, limiting the generation of analogues with predictable macrocyclic cores. In this study, we identified an unusual tri-thioesterase (tri-TE) architecture in the enduracidin NRPS assembly line, comprising one type I thioesterase domain and two type II thioesterases that contribute to enduracidin biosynthesis and assembly-line function. To explore the potential of TE repositioning, we systematically relocated the terminal thioesterase domain (EndC_TE) within the 2.0 MDa enduracidin NRPS, generating a series of engineered assembly lines that produced macrocyclic peptides with programmed backbone lengths. Notably, MS/MS analyses indicate that EndC_TE retains a conserved regioselectivity across the derivatives, with macrocyclization occurring between the invariant second threonine residue and the C-terminal carboxylate across substrates of varying lengths. Overall, our work demonstrates that TE domain relocation is a feasible strategy for reprogramming the complex enduracidin NRPS assembly line, providing a useful reference for future engineering efforts. It enables the generation of macrocyclic derivatives with tailored ring sizes and highlights EndC_TE as a catalytic domain with potential for constructing cyclic peptide libraries.

## Highlights

• Enduracidin gene cluster features an uncommon tri-TE architecture.
• Targeted editing of a megadalton NRPS assembly line via a TE relocation strategy.
• TE-relocated NRPSs produced macrocyclic peptides with programmed ring sizes.
• EndC_TE couples substrate-length tolerance with stringent regioselectivity.

## Graphical abstract

**Figure:**
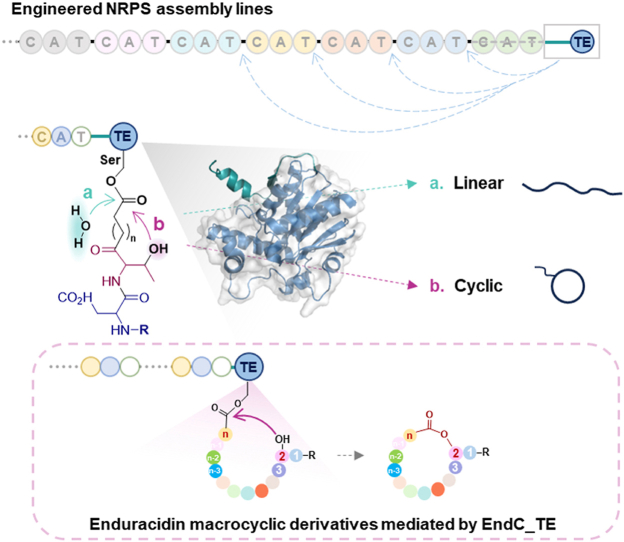

## 1 Introduction

Cyclic peptides represent an essential class of bioactive molecules distinguished by their conformational rigidity, improved stability, and enhanced target affinity relative to linear peptides [1]. Up to 2024, at least 66 cyclic peptides or their derivatives have been approved for clinical use [2] and applied across a wide range of therapeutic areas [2,3]. While diverse chemical macrocyclization strategies exist, achieving chemo-, regio-, and stereo-selectivity remains a challenging task [4,5].

In nature, many cyclic peptides are biosynthesized by nonribosomal peptide synthetases (NRPSs), whose cyclization mechanism and structural composition are more complex than those performed by chemical approaches [6,7]. However, the modular architecture of NRPSs provides an ideal platform for rational engineering of desirable nonribosomal peptide (NRP) analogues by modifying the modules or domains within the assembly line [[8], [9], [10], [11]]. A typical NRPS module consists of an adenylation (A) domain for substrate selection and activation, a peptidyl carrier protein (PCP/T) domain for intermediate tethering, and a condensation (C) domain for peptide bond formation, while optional tailoring domains further diversify the product [6,12]. The C-terminal type I thioesterase (TEI) domain functions as a gatekeeper in NRP biosynthesis by governing product release [[13], [14], [15]]. It catalyzes either intramolecular macrocyclization to yield macrolactones or macrolactams via head-to-tail or side-chain-mediated linkages, or hydrolysis to release linear peptides [13,15,16]. This step represents the terminal stage of NRPS-mediated biosynthesis and plays a central role in defining the structural scaffold and biological activity of the products [17]. In addition to TEI, some NRPS systems involve independently expressed type II thioesterases (TEIIs), which are typically involved in editing or pathway maintenance [[18], [19], [20]], but have also been shown to mediate the shuttling [21] or transfer of activated aminoacyl intermediates [22] in certain noncanonical NRPS systems. Despite this apparent modularity, NRPS assembly lines operate through highly coordinated interdomain interactions, and efficient peptide biosynthesis depends on precise structural and functional coupling between domains [12,23,24]. Consequently, engineering of multi-megadalton NRPS systems remains challenging, as perturbations to individual modules or domains can disrupt substrate transfer, intermediate processing, or overall assembly-line efficiency [25].

TEIs have attracted considerable interest as potential tools for NRPS engineering [26,27]. However, their application remains constrained by inherent substrate specificity and stringent regioselectivity. Beyond intrinsic catalytic properties, TE-mediated macrocyclization is a dynamic process governed by precise substrate presentation and conformational compatibility at the T–TE interface [23,[28], [29], [30]]. Successful macrocyclization depends on coordinated interactions between the T and TE domains, as well as the segmental flexibility of the TE “lid”, which collectively ensure the proper orientation of the tethered peptide for nucleophilic attack [29,31]. While several primary studies have demonstrated that isolated TEIs can serve as effective catalysts for the cyclization of synthetic peptide substrates in vitro, achieving efficient and selective production of defined cyclic peptides remains challenging [27,32,33]. In this context, previous studies have explored strategies such as internal module deletion [[34], [35], [36]] or TE translocation [37,38] to alter peptide chain length and modulate ring size. These studies showed that cyclic peptide variants could be generated, although reduced product yields or increased hydrolysis products were also observed in some cases [34,35,37,38], underscoring the sensitivity of NRPS assembly lines to structural perturbation and altered interdomain interactions. Collectively, these approaches provide feasible routes for constructing engineered NRPS systems and for probing TE substrate tolerance and regioselectivity.

Our study focuses on the enduracidin biosynthetic gene cluster, whose biosynthetic logic has been largely elucidated [39]. A schematic overview is provided in Fig. S40. This cluster features a predominantly linear NRPS assembly line, providing an ideal platform for manipulation of its biosynthetic machinery. Enduracidin, produced by *Streptomyces fungicidicus* TXX3120*,* consists of 17 amino acid residues and a fatty acid chain. Its macrocyclization is achieved through intramolecular ester bond formation between the hydroxyl group of the second residue (threonine, Thr) and the carboxylate of the seventeenth residue (hydroxyphenylglycine, Hpg).

In this study, we identify EndC_TE as the key thioesterase responsible for macrocyclization within the enduracidin biosynthetic system and leverage this knowledge to reprogram the NRPS assembly line. By combining targeted internal module deletions with strategic relocation of the EndC_TE domain using CRISPR-Cas9, we construct a series of module-reduced assembly lines that generate truncated enduracidin derivatives with distinct cyclic-to-linear product distributions. MS/MS analyses suggest that, despite variations in substrate length, EndC_TE catalyzes macrocyclization at a conserved regioselective site consistent with that of native enduracidin. These results reveal that EndC_TE accommodates substantial variation in substrate length while maintaining strict regioselectivity. Collectively, this work provides a detailed demonstration of accessing truncated NRP derivatives and illustrates the utility of TE domain relocation as a viable strategy for reprogramming the massive enduracidin NRPS assembly line toward the generation of structurally diverse cyclic peptides.

## 2 Material and methods

### 2.1 Strains, plasmids and reagents

The strains and plasmids used in this study are listed in Tables S1 and S2, respectively. *Streptomyces fungicidicus* TXX3120 (CICC11059), an industrial strain producing enduracidin, was supplied by Xinxing Veterinary Pharmaceutical Co., Ltd. (Tianjin, China). The derivative strain SFA was generated by targeted deletion of the native *upp* (uracil phosphoribosyltransferase) gene from *S. fungicidicus* TXX3120 and was used as the parent strain to construct various recombinant mutants. *Bacillus subtilis* CMCC (B) 63501, obtained from the China Institute of Veterinary Drug Control (Beijing, China), was used as the indicator strain to test the antibacterial activity of enduracidin. *Escherichia coli* JM109 was used for DNA cloning, while *E. coli* ET12567 harboring pUZ8002 was used as the donor strain for intergeneric conjugation with *Streptomyces*. The gene-editing plasmid pCas9-*upp*, previously constructed in our laboratory [40], was used for targeted gene editing.

Oligonucleotide synthesis (listed in Table S3) and Sanger sequencing service were provided by Genewiz (Tianjin, China). PCR amplifications were performed using ApexHF HS DNA polymerase (Accurate Biotech, Changsha, China). Restriction enzymes were obtained from Takara (Dalian, China), and the pEASY®-Basic Seamless Cloning and Assembly Kit (TransGen Biotech, Beijing, China) was used for plasmid assembly. DNA fragments from agarose gels and PCR products were purified using the Gel Extraction Kit and Cycle Pure Kit (OMEGA, USA), respectively. Plasmid DNA isolation from *E. coli* cultures was performed using the Plasmid Mini Kit (OMEGA). The enduracidin standard (4% purity) was purchased from Yuanye Bio-Technology (Shanghai, China).

### 2.2 Media, culture conditions and transformation methods

*Escherichia coli* strains were cultivated at 37 °C in Luria–Bertani (LB) medium (10 g/L tryptone, 5 g/L yeast extract, 10 g/L NaCl) supplemented with the appropriate antibiotics as required. Plasmid isolation, bacterial transformation, and competent cell preparation followed standard protocols described in *Molecular Cloning: A Laboratory Manual* [41].

*Streptomyces fungicidicus* TXX3120 and its derivative mutants were cultured on mannitol soya (MS) agar plates (20 g/L mannitol, 20 g/L soybean flour, 20 g/L agar) at 28 °C for 7 days to promote sporulation. For seed culture preparation, freshly harvested spores were inoculated into 250 mL flasks containing 50 mL seed medium (22.4 g/L corn steep powder, 20 g/L CaCO_3_, 5 g/L cottonseed cake powder, 5 g/L (NH_4_)_2_SO_4_, 1.143 g/L FeSO_4_, 0.065 g/L KH_2_PO_4_, 35 g/L corn flour; pH 7.2). The cultures were incubated at 28 °C with shaking at 220 rpm for 48 h. The resulting seed cultures were incubated into 500 mL shaking flasks containing 50 mL fermentation medium (20 g/L corn flour, 15 g/L HEPES; pH 7.2) at a 10% (v/v) inoculation rate, followed by cultivation at 28 °C and 220 rpm for 7 days.

Plasmid transfer from *E. coli* to *Streptomyces* was achieved via intergeneric conjugation. The donor strain *E. coli* ET12567/pUZ8002 was cultivated on 2 × YT medium (16 g/L tryptone, 10 g/L yeast extract, 5 g/L NaCl) at 37 °C with antibiotics (50 mg/L apramycin, 25 mg/L chloramphenicol, and 50 mg/L kanamycin). Cells were harvested at an OD_600_ of 0.4–0.6, washed three times with ice-cold LB medium, and kept at 4 °C until use. *S. fungicidicus* spores were suspended in 2 × YT medium, heat-shocked at 50 °C for 10 min, and preincubated at 28 °C for 3 h before centrifugation. MS agar plates supplemented with 10 mM MgCl_2_ were used for conjugation. After 18 h incubation at 28 °C, the plates were overlaid with sterile water containing 50 mg/L apramycin. The plates were incubated for an additional 5–7 days until exconjugants could be detected and verified.

### 2.3 Construction of TE domain site-directed mutants and complemented strains

The plasmid pKC1139-*upp*, a derivative of pKC1139, served as the backbone for constructing site-directed mutation vectors. All primers are listed in Table S3. For construction, pKC1139-*upp* was linearized by digestion with *HindIII* and *XbaI* and gel-purified. Homologous arms (HAs) flanking the desired mutation were amplified from *S. fungicidicus* TXX3120 genomic DNA using the primer pairs listed in Table S3. As a representative example, pKC1139-620S104A was constructed as follows: the upstream HA was amplified with the primer pair pkc620_S104A-Hindiii-upF/pkc620_S104A-upR and the downstream HA with the primer pair pkc620_S104A-downF2/pkc620_S104A-Xbai-downR2. The two HA fragments, containing the codon substitution (Serine to Alanine, corresponding to TCC to GCG), were gel-purified and assembled into the *HindIII/XbaI* sites of pKC1139-*upp* by seamless cloning to yield pKC1139-620S104A. All mutation plasmids were verified by Sanger sequencing prior to conjugation.

pKC1139-based plasmids were transferred into the SFA parental strain by intergeneric conjugation. The ex-conjugants were screened sequentially to confirm the occurrence of single- and double-crossover events. The double-crossover mutants were screened by PCR using primers flanking the HAs (e.g., Kno25620-cF2/Kno25620-cR2) and confirmed by Sanger sequencing. The mutant strain carrying the intended codon replacement in the TE domain was named accordingly (e.g., S104A). Other site-directed mutants were constructed through similar procedures.

Complementation constructs were generated using the integrative vector pSET152. pSET152 was linearized with *EcoRI/XbaI* and gel-purified. The *stnYp1* promoter [42] and coding sequences (for example, *CNQ36_25620*) were amplified using the primer pairs listed in Table S3 (e.g., p152-stnYp1-rbs-F/R and P152-25620-F/R), gel-purified, and assembled into pSET152 by seamless cloning to yield pSET152-stnYp1-25620. Other complement plasmids were generated by replacing the gene insert of pSET152-stnYp1-25620 via *NdeI/XbaI* cloning. For EndC_TE domain complementation, the fragment was designed to include full EndC_TE domain coding sequence, the T domain of module 17, and the intervening linker. An ATG start codon was introduced at the 5′ end, and the fragment was cloned downstream of *stnYp1* to generate pSET152-stnYp1-EndC_TE. All plasmids were sequence-verified before being introduced into the corresponding mutant strains.

### 2.4 Construction of EndC_TE domain relocation mutants using CRISPR/Cas9

The TE domain relocation strategy was designed by deleting different numbers of complete modules from the enduracidin NRPS assembly line, and EndC_TE domain was fused to the C-terminus of the T domain of the corresponding target modules, thereby generating multiple reprogrammed NRPS assembly lines. The pCas9-*upp* plasmid served as the backbone for constructing EndC_TE domain relocation strains. To avoid potential conflicts between sgRNA restriction site and restriction sites within the HAs, multiple pCas9-*upp* derivatives carrying alternative sgRNA restriction sites (*NcoI, BamHI*, and *AflII*) were constructed. First, pCas9-*upp* was linearized with *XbaI* and gel-purified for seamless cloning of HAs. The resulting constructs were sequence-verified prior to sgRNA insertion. All sgRNAs were designed using the CRISPy-web [43] and three target sites were selected for each edit.

For example, to construct pCas9-*upp*-M14-sg1, the upstream HA was amplified with primers P_cap_xbaI-te1+2-14up-F/R, and the downstream HA with primers P_cap_xbaI-te1+2-ndown-F/R. The fragment containing sgRNA was generated by annealing of primers TE1+2-14Gly-sgRNA1-F/R and then cloned into the *BamHI* site of the linearized HA-containing plasmid. The primer cb-mcsF was used for Sanger sequencing of the sgRNA insertion. The pCas9-*upp*-based plasmids for TE domain relocation were constructed following similar steps.

The recombinant plasmids were transformed into SFA by intergeneric conjugation. Ex-conjugants were sequence-verified by colony PCR using primers flanking the HAs. The correct constructs were subsequently subjected to plasmid curing to obtain the recombinant strain with TE domain relocation to module 14, designated M14. Other recombinant strains were constructed through the same procedure.

### 2.5 HPLC analysis of fermentation extract

For extraction and analysis of enduracidin, the mycelial pellet, obtained from 1 mL of fermentation broth by centrifugation, was extracted with 2 mL of extraction reagent (acetone/1 M HCl/ddH_2_O, 35:12:56, v/v/v) for 3.5 h. The extract was centrifuged to remove particulate matter, and the supernatant was filtered (0.22 μm) before analysis. The sample was analyzed using an Agilent 1260 Infinity II High Performance Liquid Chromatography (HPLC) system (Agilent Technologies, USA) equipped with an Agilent Eclipse XDB-C18 column (250 × 4.6 mm, 5 μm). A 10 μL sample was injected, and the mobile phase consisted of 30% acetonitrile (solvent A) and 70% 50 mM NaH_2_PO_4_ buffer (pH 4.5, solvent B) at a flow rate of 1.0 mL/min. Detection was performed at 267 nm, and data analysis was conducted using *Agilent OpenLab CDS* software.

### 2.6 LC–HRMS and LC–HRMS/MS analysis of enduracidin and its derivatives

High-resolution mass spectrometry (HRMS) analysis was performed using an Agilent 1290 Infinity Ultra-High Performance Liquid Chromatography (UHPLC) system coupled with an Agilent 6545B Q-TOF mass spectrometer (Agilent Technologies, USA). Electrospray ionization (ESI) was employed as the ion source. Chromatographic separation was achieved on an Agilent Eclipse XDB-C18 column (150 × 2.1 mm, 3.5 μm particle size). The mobile phase consisted of 1% formic acid in water (solvent A) and 1% formic acid in methanol (solvent B), operated under a suitable gradient elution program at a flow rate of 0.2 mL/min, with the following gradient: 0–1 min 10% B, 1–3 min 10–30% B, 3–15 min 30–70% B, 15–17 min 70–95% B, 17–20 min 95–10% B, 20–22 min 10% B. Data acquisition and processing were conducted using *Agilent MassHunter Workstation Software* (Agilent Technologies, USA).

MS/MS data were acquired on an Agilent 6545B Q-TOF mass spectrometer equipped with an electrospray ionization (ESI) source and operated in positive ion mode using auto MS/MS acquisition. The top three most intense precursor ions per cycle were selected for fragmentation based on an absolute intensity threshold of 200 counts. Precursor ions with charge states of +3, +2, and +1 were preferentially selected. Isolation was performed with a window of 4 *m/z*. Fragmentation was carried out using collision-induced dissociation (CID) with nitrogen as the collision gas, and collision energies were optimized within a range of 10–40 eV, depending on the precursor *m/z* and charge state. The precursor ions and collision energies corresponding to each MS/MS spectrum were annotated in the Supplementary Figure captions. MS and MS/MS spectra were acquired over an *m/z* range of 100–3000 at a rate of 1 spectrum/s. The fragmentor voltage was set to 175 V. The source parameters were set as follows: gas temperature, 320 °C; drying gas flow, 8 L/min; nebulizer pressure, 35 psi; capillary voltage, 3500 V.

Raw mass spectrometry data were processed using *Agilent MassHunter Workstation Software* (Qualitative Workflows, version B.08.00, Agilent Technologies). Initial compound identification was performed using the *Find by Formula algorithm* (Agilent MassHunter software) in the *Target/Suspect Screening* workflow. This algorithm detects chromatographic peaks in the LC–HRMS dataset that match the theoretical exact mass and isotopic distribution for a predefined list of molecular formulas. For instance, the compound End_C9 (C_63_H_86_N_12_O_19_; monoisotopic mass = 1314.6139 Da) was successfully identified with a retention time of 16.774 min, a matching score of 98.65, and a mass error of 0.52 ppm. The detailed output parameters including formula, mass, retention time (RT), match score, mass error (ppm), and spectral hit count are summarized in Fig. S13. Compounds with scores ≥90 were considered confidently identified, whereas those with scores <70 were regarded as tentative identifications, usually due to low signal intensity (<10^2^–10^3^ counts); compounds scoring below 60 were considered unreliable and were excluded from further analysis. The *MS Spectrum Results panel* was used to further validate assignments by checking isotopic distributions and charge states. Experimental spectra corresponding to different adducts and charge states (e.g., [M+H]^+^, [M+2H]^2+^, [M+3H]^3+^ and [M+Na]^+^) were extracted and compared to their theoretical *m/z* values, detailed in Table S9.

For further confirmation, tandem mass (MS/MS) spectrometry data were analyzed in *MassHunter Qualitative Navigator* software. We predicted the product structures and possible fragments based on the engineering assembly lines, then we calculated the theoretical *m/z* values of precursors and fragments for the singly, doubly, and triply charged species using *Molecular Mass Calculator* (place within Agilent MassHunter software). The precursor ion *m/z* values were used to generate extracted ion chromatograms (EICs), then the integrated peak areas of EICs were used to calculate the cyclic-to-linear ratios of the products.

Peptide fragmentation typically occurs at amide bonds, yielding a series of complementary b and y ions, while cyclic peptides preferentially cleave at side chains outside the ring. Enduracidin contains 17 amino acid residues, and its relatively large molecular size allows the precursor and fragment ions to be detected in multiple charge states (e.g., +1 to +3).

The cyclic enduracidin (End_CA) and its cyclic derivatives consistently generated characteristic fragment ions derived from side-chain cleavage (b ions) as well as the macrocyclic backbone fragments (y and z ions); The linear enduracidin (End_LA) and its linear derivatives consistently generated characteristic fragment b and y ions. Fragment ion assignments were validated by matching experimental MS/MS spectra to the predicted fragment ions based on mass accuracy (reported in ppm), as summarized in Tables S10–S23. Annotated MS/MS spectra, including isotopic distributions of the fragment ions, are presented in Figs. S13–S36. For low-abundance fragment ions, the MS/MS spectra were magnified for clarity. In certain cases (e.g., End_L9 and End_L16), the low-yield products affected the selection and fragmentation of precursor ions, leading to lower abundance ions that impact the mass spectra quality (Figs. S15 and S32), which were used as a reference.

### 2.7 Web servers and software used in this study

Secondary metabolite biosynthetic gene clusters (BGCs) were predicted using antiSMASH Bacterial 6.0.1 [44]. Homologous BGCs were identified using the built-in comparative analysis module of antiSMASH. Detailed domain annotation and substrate specificity were also obtained from antiSMASH. Gene cluster comparison figures were generated using CAGECAT-clinker [45]. Protein structures were predicted using AlphaFold3 [46] and subsequently visualized and structurally aligned in PyMOL 3.0 [47]. Amino acid sequences were aligned using MEGA-X [48], and the alignments were visualized with ESPript 3.0 [49]. Phylogenetic trees were constructed using the maximum-likelihood method implemented in MEGA-X and further visualized using ChiPlot [50]. The sgRNAs were designed using the CRISPy-web online server [43]. NRPS domain architecture was predicted using the PKS/NRPS Analysis Web-site [51].

## 3 Results

### 3.1 Three TEs in the enduracidin gene cluster

**Fig. 1:**
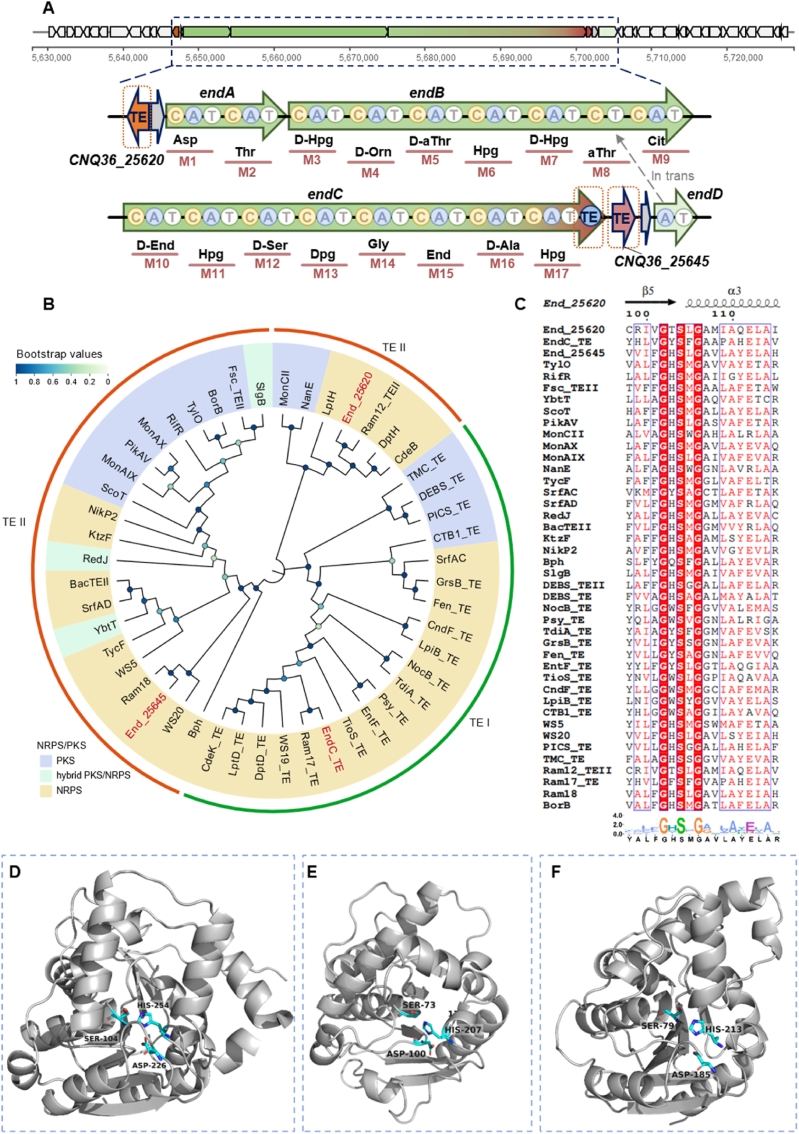
Phylogenetic and structural analyses of three thioesterase (TE) domains. (A) Gene distribution of the enduracidin biosynthetic gene cluster, with the three TE-encoding genes highlighted in red. End_25620 (gene ID: *CNQ36_25620*) is located upstream of *endA*, End_25645 (gene ID: *CNQ36_25645*) is located downstream of *endC*, and EndC_TE is an embedded C-terminal TE domain within *endC* (gene ID: *CNQ36_25640*). (B) Phylogenetic tree of the three TEs together with representative TEs from PKS, NRPS, and PKS/NRPS hybrid systems. Details of these TEs are listed in Table S24. (C) Multiple sequence alignment of the catalytic cores of these TEs, performed using MEGA-X and visualized with ESPript3, reveals the conserved GxSxG motif. (D-F) AlphaFold3-predicted structures of the three TEs, with their catalytic Ser-His-Asp triads shown in stick representation.

The enduracidin biosynthetic gene cluster encodes four NRPSs (EndA, EndB, EndC and EndD), comprising a total of 17 modules. Through bioinformatic analysis, we identified three TE domains with catalytic potential: one TEI, designated EndC_TE, located at the C-terminus of the NRPS EndC; and two stand-alone TEIIs, End_25620 and End_25645 (Fig. 1A).

These three TEs are evolutionarily conserved, retaining the characteristic α/β-hydrolase fold, the Ser-His-Asp catalytic triad, and the conserved GXSXG motif (Fig. 1C–F, Figs. S2-S3). Phylogenetic analysis including reported TE domains from PKS/NRPS systems showed that the three TEs are distantly related, suggesting that they may play distinct functional roles in enduracidin biosynthesis (Fig. 1B).

In canonical NRPS systems, macrocyclization is typically catalyzed by a single C-terminal TEI. The presence of two additional TEIIs in the enduracidin gene cluster indicates a non-canonical TE architecture, suggesting that these enzymes may perform distinct or complementary functions. However, the specific roles of the three TEs in enduracidin biosynthesis remain to be elucidated.

### 3.2 Functional characterization of three TEs via gene mutation and complementation

**Fig. 2:**
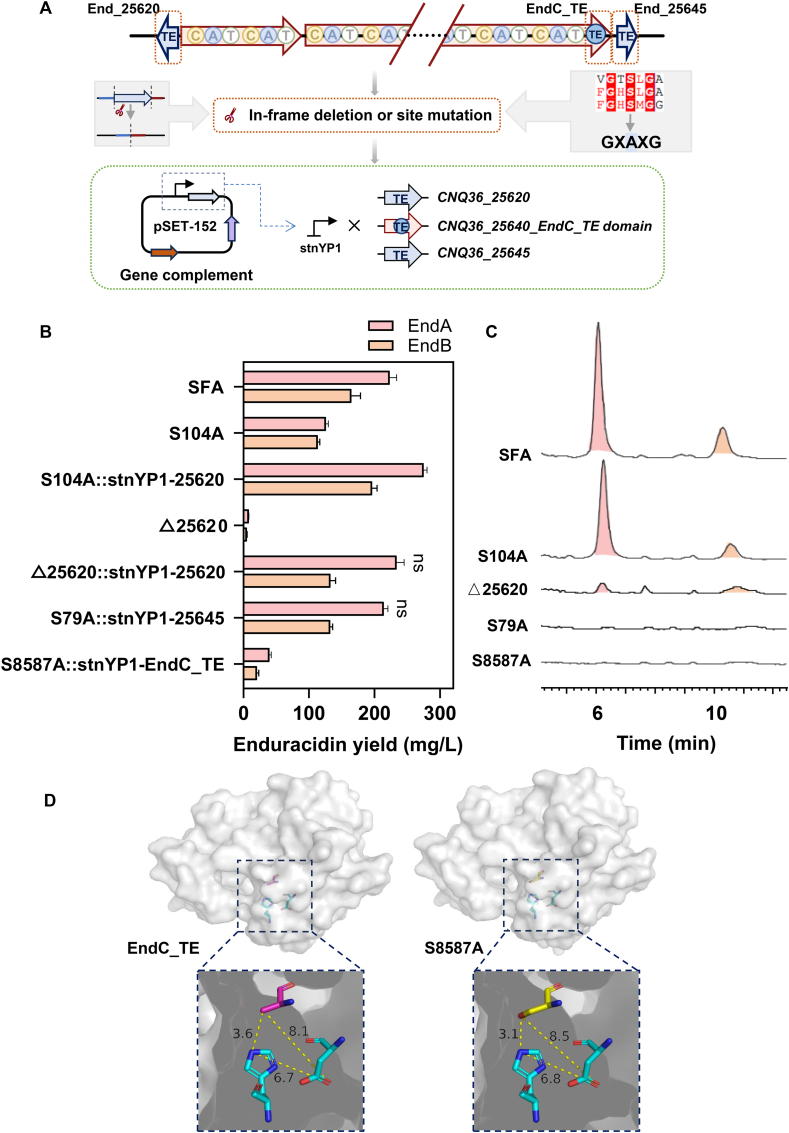
Functional validation and mechanistic insights into the three TEs in enduracidin biosynthesis. (A) Schematic representation of site-directed Ser-to-Ala mutations, in-frame deletions, and gene complementation constructs targeting the three TE domains. (B) Enduracidin production levels in the wild-type strain SFA and the corresponding engineered strains. Statistical significance was assessed using Dunnett’s multiple comparisons test (α = 0.05), with SFA as the reference group. Error bars represent standard deviations from three independent biological replicates. Unless marked as ns (not significant) in the figure, all comparisons are statistically significant (P < 0.0001). EndA and EndB denote enduracidin A and enduracidin B, respectively. (C) HPLC analysis of enduracidin production in the wild-type strain SFA and the mutants. (D) Comparison of the active sites of EndC_TE and its mutant S8587A. Hydrogen bonds between the catalytic triad Ser8587, His8614, and Asp8721 are indicated by dashed yellow lines. Structural details of End_25620 and End_25645 are provided in Figs. S10–S12.

The Ser-to-Ala substitution of the catalytic triad is a classical approach for investigating enzyme activity of the hydrolases. To determine whether the three TEs participate in enduracidin biosynthesis, we constructed mutants by replacing the conserved catalytic serine residue with alanine in each TE domain (Fig. 2A).

The S104A mutant (S104A in End_25620) showed a 43.66% reduction in enduracidin production (Fig. 2B). In contrast, the S8587A mutant (S8587A in EndC) and the S79A mutant (S79A in End_25645) completely abolished enduracidin production (Fig. 2C). These results indicate that EndC_TE and End_25645 are indispensable in enduracidin biosynthesis, whereas End_25620 contributes to production efficiency.

To further verify these observations, complementation experiments were performed using the pSET-152 vector to express the corresponding wild-type genes under the control of the constitutive *stnYp1* promoter [42]. Compared with the wild-type strain SFA, the complemented strain S104A:stnYp1-25620 restored enduracidin production and exhibited a 23.2% increase (P < 0.0001). The complemented strain S79A:stnYp1-25645 also restored production, whereas the complemented strain S8587A:stnYp1-EndC_TE only recovered 17.79% of the wild-type level (Fig. 2B).

We further constructed an in-frame deletion mutant, designated Δ25620, to evaluate the functional role of End_25620. The Δ25620 mutant retained only approximately 8% of wild-type production, indicating a more severe defect than that observed in the S104A mutant. Complementation with *CNQ36_25620* restored production to approximately 104.6% of the wild-type level (Fig. 2B), confirming that the reduced production in the Δ25620 mutant was caused by the loss of End_25620.

Together, these results demonstrate that all three TEs participate in enduracidin biosynthesis. EndC_TE and End_25645 are indispensable for product formation, whereas End_25620 plays a supporting but significant role in maintaining efficient biosynthesis.

### 3.3 Functional analysis of three TEs based on bioinformatics

**Fig. 3:**
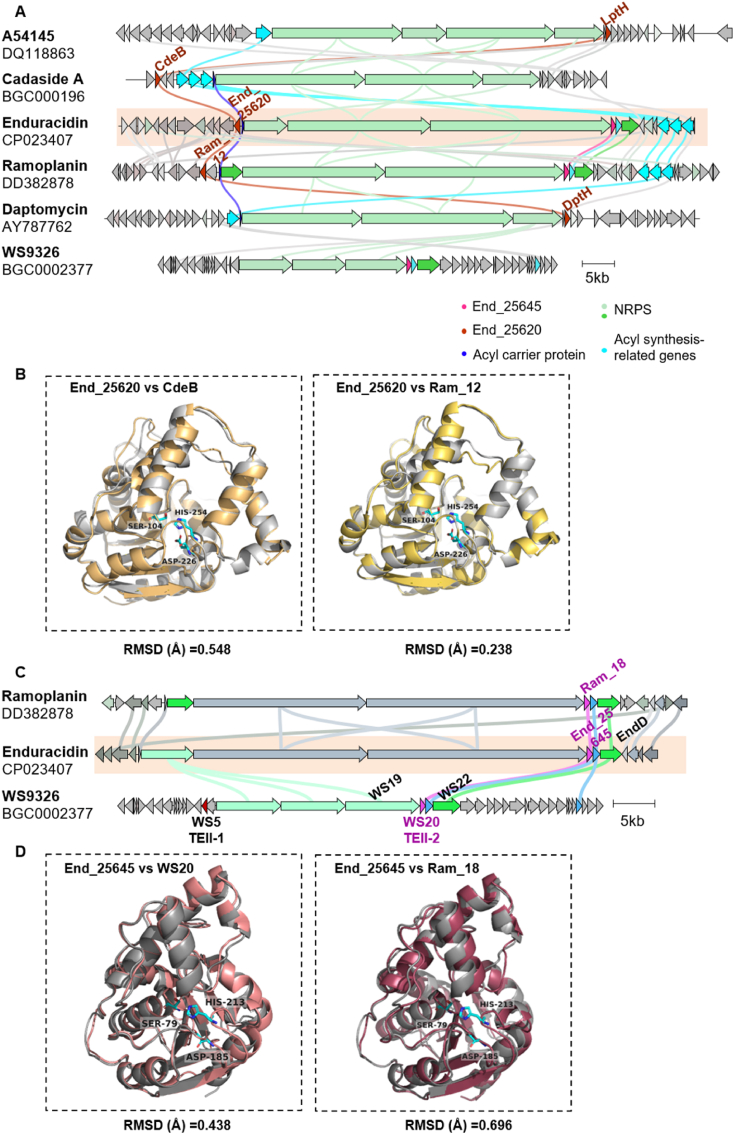
Comparative analysis of the enduracidin gene cluster and structural alignment of its TEs with homologs. (A) Comparative gene cluster alignment of enduracidin with A54145, cadaside A, ramoplanin, daptomycin, and WS9326. (B) Structural alignment of End_25620 with its TEII homologs CdeB and Ram_12. Detailed alignment parameters are summarized in Table S5. (C) Comparative gene cluster alignment of enduracidin, ramoplanin and WS9326. (D) Structural alignment of End_25645 with its TEII homologs WS20 and Ram_18. Detailed alignment parameters are summarized in Table S6.

Transcriptomic analysis of *S. fungicidicus* across different fermentation stages showed that *CNQ36_25620*, encoding End_25620 and located upstream of *endA* (*CNQ36_25630*), was consistently highly expressed during enduracidin biosynthesis (Fig. S1). Phylogenetic and structural analyses revealed that End_25620 shares high homology with TEIIs from several lipopeptide biosynthetic gene clusters, including DptH [52], LptH [53], CdeB [54], and Ram_12 [55] (Fig. 1B, Fig. 3A–B, Figs. S6 and S8). These observations support the assignment of End_25620 as a typical TEII. The S104A mutant retained partial production, further indicating that End_25620 plays a contributory but non-essential role in enduracidin biosynthesis.

The enduracidin biosynthetic gene cluster contains four NRPSs [39], whose modular architecture corresponds to the 17 amino acid residues of the peptide scaffold, while containing partially nonlinear features. Specifically, the eighth module of the second NRPS, EndB, lacks an A domain, whereas the fourth NRPS, EndD, is a standalone module NRPS containing an A–T didomain. The chemical structure of enduracidin indicates that the eighth amino acid residue is L-allo-Thr (Fig. S8). Consistent with this feature, bioinformatic analysis predicted that the adenylation (A) domain of EndD specifically recognizes L-allo-Thr (Fig. S38). To verify its essential role in enduracidin biosynthesis, we constructed an in-frame deletion mutant of *endD,* which completely abolished the production of enduracidin (Fig. S37). These observations suggest a potential functional relationship between EndD and the A domain-deficient module of EndB.

Another TEII, End_25645 (gene ID: *CNQ36_25645*), is located between *endC* and *endD,* and also exhibited sustained high transcription during fermentation (Fig. S1). Phylogenetic analysis showed that End_25645 shares high homology with WS20, a TEII involved in the nonlinear biosynthesis of WS9326A [21] (Figs. 1B and 3C). In the WS9326 system, WS20 mediates substrate shuttling by transferring the activated threonine—loaded by a stand-alone A–T NRPS module (WS22)—to the A domain–deficient seventh module of the NRPS WS19, thereby ensuring the continuity of the NRPS assembly line (Fig. S5) [21]. Notably, both biosynthetic systems exhibit analogous nonlinear assembly architectures. Structural alignment between End_25645 and WS20 further supported their remarkable conservation, with an RMSD value of 0.438 Å across 233 aligned residues and a MatchAlign score of 714 (Figs. 3D, S5 and Table S6). These observations suggest that End_25645 may perform a function similar to that of WS20 in enduracidin biosynthesis. Consistently, the S79A mutant completely abolished enduracidin production, indicating that End_25645 is essential for biosynthesis.

EndC_TE is a canonical TEI responsible for product cyclization and release. Mutation of its catalytic serine residue abolished enduracidin production, confirming its essential role in the final biosynthetic step. Sequence and structural comparisons showed that EndC_TE is most closely related to Ram18 [55,56], consistent with the structural similarity between enduracidin and ramoplanin (Fig. 1B, Figs. S7 and S8). Despite overall structural conservation between EndC_TE and other TEIs, variations in RMSD and alignment scores among TEIs suggest potential differences in substrate recognition and catalytic properties (Table S7).

Together, these bioinformatic and experimental analyses indicate that the three TEs in the enduracidin gene cluster play distinct roles, with EndC_TE serving as the terminal TEI, and End_25620 and End_25645 acting as TEIIs with distinct contributions to the biosynthetic process.

### 3.4 Engineering enduracidin NRPS assembly line via EndC_TE relocation

**Fig. 4:**
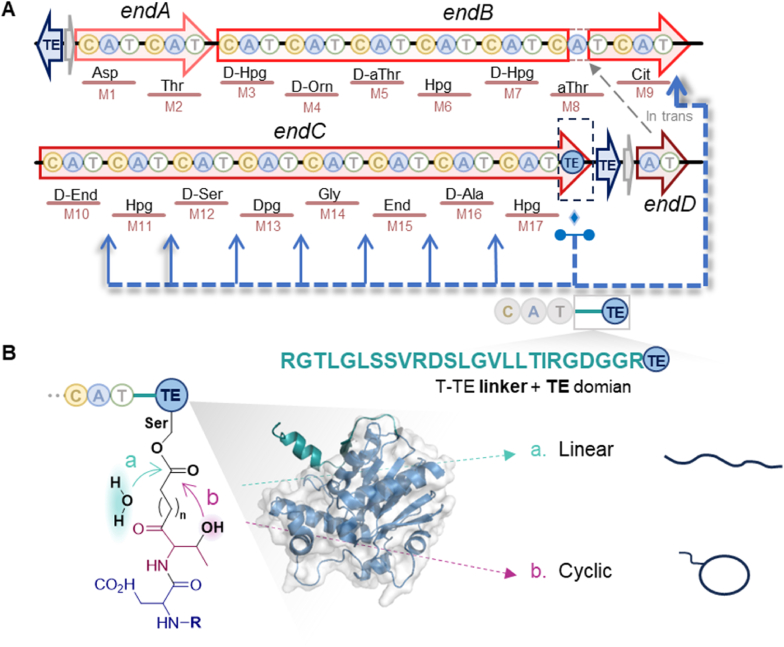
Engineering NRPS assembly lines via EndC_TE relocation. (A) Schematic representation of the EndC_TE relocation strategy. The native T–TE linker together with the EndC_TE domain was inserted after distinct modules (M9–M16) within the enduracidin NRPS assembly line. (B) EndC_TE catalyzes product release via macrocyclization or hydrolysis. The final product is produced in either linear or cyclic form, depending on whether the nucleophile is the intramolecular hydroxyl group of the second Thr residue or an external water molecule.

**Fig. 5:**
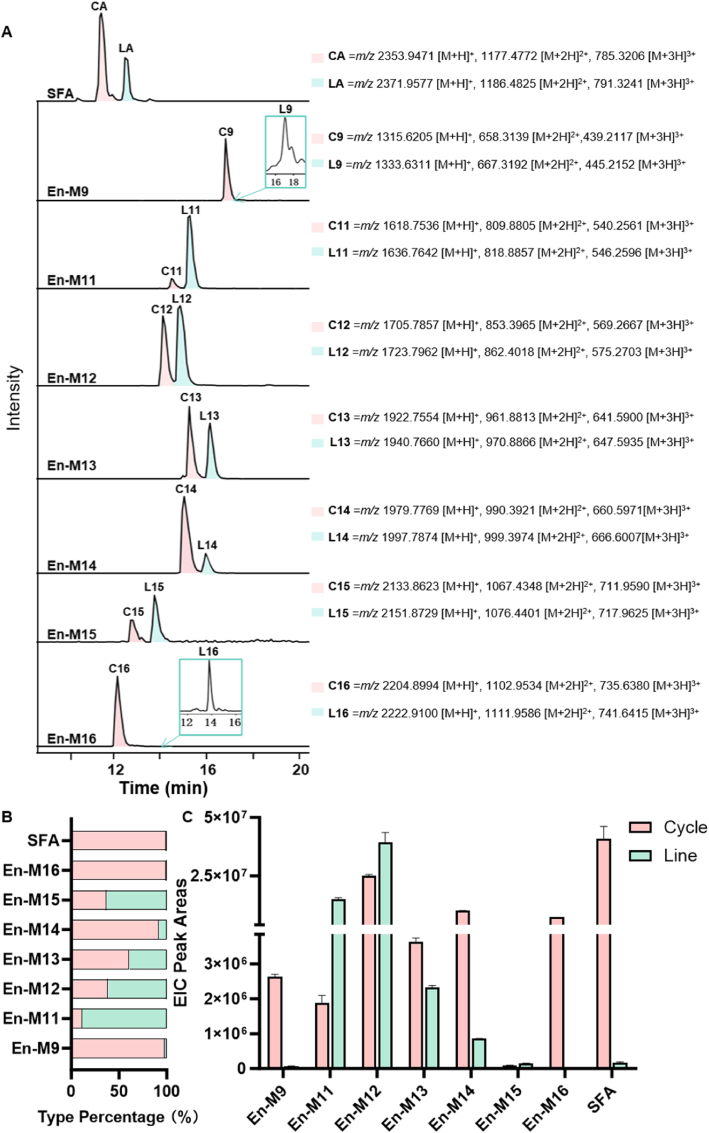
Production of cyclic and linear peptides by the engineered NRPS assembly lines via TE domain relocation. (A) LC–HRMS analysis of cyclic and linear peptides generated by the engineered strains. Extracted ion chromatograms (EICs) are shown for the +1, +2, and +3 charge states of each molecule. (B) Product profile composition. Stacked bars represent the relative proportion of the cyclic versus linear peptide for each engineered strain and SFA, calculated from their respective LC–HRMS EIC peak areas. (C) Relative EIC peak areas. Bar graphs show the LC–HRMS EIC peak areas for the cyclic and linear peptides produced by each engineered strain and the wild-type SFA. Values and error bars represent the mean ± standard deviation of three independent experiments.

For the complex megasynthase assembly lines of enduracidin, targeted engineering modifications of it remain challenging. To reduce disturbance to the assembly line, we adopted a TE domain relocation strategy by deleting multiple contiguous upstream modules and repositioning the EndC_TE domain within the assembly line. Previous studies have demonstrated that the native linker region is critical for TE catalytic function [37,57]. We retained the native linker between module 17 and EndC_TE in all engineered assembly lines. The EndC_TE domain was relocated downstream of the T domains of modules 9–16, generating eight artificial NRPS assembly lines (Fig. 4A), corresponding to strains En-M9 to En-M16. LC–HRMS analyses showed that seven strains, En-M9 and En-M11 to En-M16, produced the corresponding expected enduracidin derivatives (Fig. 5A), whereas no corresponding cyclic or linear products were detected for En-M10 under the applied conditions.

For example, the cyclic product End_C9 generated from the nine-module engineered assembly line, exhibited ions at *m/z* values of 1315.6205 and 658.3139, corresponding to the predicted [M+H]^+^ and [M+2H]^2+^ ions (Fig. S13), with mass errors of 1.67 ppm and 2.43 ppm (Table S10), respectively.

Structural assignments were supported by HRMS data and detailed comparisons of MS and MS/MS ion fragmentation patterns (Supplementary Tables S9-S23 and Figs. S13-S36). Additional support was obtained from isotopic distribution and charge-state patterns of these molecules. For derivatives produced by the 14–16 module assembly lines and the native system, characteristic chlorine isotope patterns provided additional confirmation. The isotopic distributions of precursor ions were evaluated using theoretical patterns calculated with *Agilent MassHunter Workstation Software* (Figs. S13-S36).

The engineered assembly lines produced both cyclic and linear products, with substantial variation in their relative proportions (Fig. 5B). En-M9, En-M14, and En-M16 predominantly yielded cyclic products (>90%), resembling the native distribution. In contrast, En-M11, En-M12, and En-M15 primarily produced linear products, with En-M11 generating up to 88.7% linear products. En-M13 showed an intermediate distribution, with an approximate cyclic-to-linear ratio of 6:4.

Under identical culture and analytical conditions, product levels varied markedly among the engineered strains (Fig. 5C). Although absolute quantification was not performed because authentic standards were unavailable, comparative LC–HRMS analyses indicated that En-M12 exhibited the highest overall product abundance, despite its higher proportion of linear products, whereas En-M9, En-M15, and En-M16 showed lower product levels. Notably, no native enduracidin was detected in any engineered strain, indicating successful reprogramming of the NRPS assembly line.

### 3.5 Macrocyclization of enduracidin cyclic derivatives

Comparative analysis of MS/MS data supported that macrocyclization in all enduracidin cyclic derivatives follows a conserved and regioselective pattern. In native enduracidin, cyclization occurs via an ester bond between the Thr2 hydroxyl group and the Hpg17 carboxylate. In all engineered cyclic derivatives, macrocyclization was assigned to occur between Thr2 and the C-terminal residue installed by the final module of the assembly line, indicating a conserved cyclization mode relative to the native product.

The MS/MS spectra of native cyclic and linear enduracidin (End_CA and End_LA, Fig. S36) were used as references for comparison with the engineered derivatives. Under CID conditions, fragmentation of the cyclic analogues preferentially generated N-terminal fragments external to the macrocycle. Stable b_1_ and b_2_ ions (theoretical *m/z* values of 179.1430 and 294.1700), together with their corresponding y_1_ and y_2_ ions, were observed across all derivatives, consistent with End_CA. The y_1_ and y_2_ ions were detected in multiple charge states (singly, doubly and/or triply charged) and exhibited isotopic distributions consistent with their theoretical predictions. Notably, b_3_ and all higher b-ions (b_n_, n > 2) were not observed in any cyclic derivatives or in End_CA. In addition, lower-abundance z-ion fragments were detected in some cyclic derivatives, providing additional support for fragment assignment.

Comparison between cyclic peptides and their linear counterparts revealed distinct fragmentation patterns. Cyclic derivatives exhibited truncated b-ion series, whereas linear congeners showed continuous series of b- and y-ions, consistent with native linear enduracidin End_LA. This difference is consistent with the peptide backbone being constrained from residue 2 onward in the cyclic structures.

**Fig. 6:**
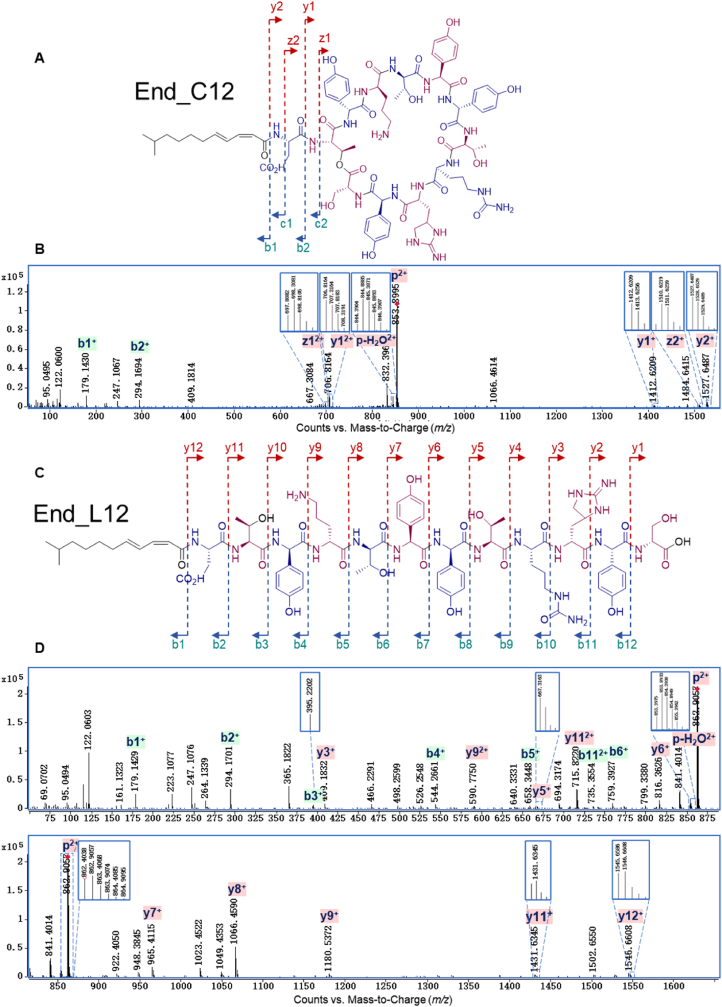
High-resolution MS/MS analysis of cyclic (End_C12) and linear (End_L12) enduracidin A analogues. (A) Proposed chemical structure of End_C12 with predicted b/y and c/z fragment ion assignments. (B) LC–HRMS/MS spectrum of End_C12, showing observed fragment ions with corresponding *m/*z values under different charge states; selected regions are enlarged for clarity. The theoretical *m/*z values and corresponding mass errors are provided in Table S13. (C) Proposed chemical structure of End_L12 with predicted b/y fragment ion assignments. (D) LC–HRMS/MS spectrum of End_L12, showing observed fragment ions with corresponding *m/*z values under different charge states; selected regions are enlarged for clarity. The theoretical *m/*z values and corresponding mass errors are provided in Table S14.

Using End_C12 as an example, this cyclic derivative was produced by an engineered assembly line consisting of the first 12 modules from the enduracidin NRPS. Its MS/MS spectrum (Fig. 6B) showed characteristic b_1_ and b_2_ ions at *m/z* 179.1430 (0.00 ppm) and 294.1694 (2.04 ppm), respectively. The y_1_ ion was detected at *m/z* 706.8164 ([M+2H]^2+^; 1.84 ppm) and 1412.6209 ([M+H]^+^; 1.49 ppm), and the y_2_ ion at *m/z* 1527.6487 ([M+H]^+^; 0.79 ppm). The isotopic distributions of these y-ions were consistent with their assigned charge states. As observed for native enduracidin, no b_3_ or higher-order b-ions were detected in End_C12, whereas its linear congener End_L12 exhibited a complete series of consecutive b- and y-ions (Fig. 6D). This fragmentation pattern is consistent with that of native enduracidin (Fig. S36), and does not align with alternative cyclization modes (e.g., 1–12 or 3–12 linkages), which supports assignment of macrocyclization between Thr2 and the C-terminal residue.

All assignable fragment ions, including those in different charge states, were annotated in the spectra (Figs. S13-36) and showed good agreement with the expected fragmentation patterns, isotopic distributions, and mass accuracy (Tables S9-S23), supporting the reliability of the fragment assignments. Together, these results support a conserved macrocyclization mode across the engineered enduracidin derivatives, consistent with that of wild-type End_CA.

## 4 Discussion

Recent studies have markedly advanced NRPS engineering by redefining recombination boundaries, including the XU [9], XUC [10], and XUT [8] concepts. These strategies have enabled efficient high-throughput generation of novel and engineered NRPS libraries and facilitated the production of structurally diverse NRP [11]. However, engineering large multi-modular NRPS systems remains challenging, as productive biosynthesis still depends on compatible interdomain interactions, efficient functional coupling between adjacent modules, and proper protein–protein interfaces [31]. In this context, previous studies have explored several engineering routes to alter peptide length and ring size in NRPS assembly lines. Early work showed that in-frame deletion of an entire module can shorten the peptide backbone and generate cyclic products with reduced ring size, while also highlighting the strong dependence of product formation on assembly-line context [[34], [35], [36]]. In the plipastatin system, forward relocation of the native TE generated truncated products and revealed that the native T–TE linker is critical for product release [37].

Building on these studies, we systematically repositioned EndC_TE at multiple internal sites in the 17–module, 2.0 MDa enduracidin NRPS. By preserving the upstream elongation modules, this design minimized perturbations to the assembly line and enabled direct evaluation of the substrate tolerance of EndC_TE and its regioselectivity in cyclization. Together, these results illustrate the application of TE relocation in a large NRPS system and provide useful insight into positional effects on substrate tolerance, product formation and macrocyclization regioselectivity, serving as a robust case study for understanding and engineering TE-mediated peptide cyclization.

Our results showed marked variation in cyclic-to-linear product ratios among the engineered assembly lines, with no obvious trend correlating with the substrate length, suggesting that EndC_TE-mediated product release does not reflect a fixed intrinsic preference for cyclization or hydrolysis. Previous in vitro studies of NRPS TEs have shown that macrocyclization is not determined by substrate length alone, for instance, the isolated TycC TE can cyclize peptide thioesters ranging from 6 to 14 residues with broadly comparable efficiency [33]. Rather, substrate preorganization and proper positioning within the TE active site appear to be critical: in the TycC system, intramolecular backbone hydrogen bonds have been proposed to stabilize such a reactive conformation, thereby restricting water access to the TE active pocket and favoring intramolecular nucleophilic attack over hydrolysis [58]. Furthermore, studies with backbone-substituted substrates further revealed that certain regions of the peptide backbone are essential for cyclization [32,[58], [59], [60]]. These observations may account for why En-M9, despite generating the shortest peptide intermediate among the constructs, still predominantly forms the cyclic product. Nevertheless, the precise structural determinants underlying this behavior remain to be further elucidated.

Preorganized conformation has also been suggested in structural studies to contribute to the regioselectivity of TE-mediated cyclization. Proper substrate fitting within the TE active site may restrict the conformational flexibility of intermediates, orient the nucleophilic group for site-specific intramolecular attack, and limit water access to the active site [29,59]. In addition, previous studies suggest that TE-mediated cyclization depends on recognition of certain key positions within the substrate, particularly the nucleophilic residue involved in ring closure and residues near the C terminus, whereas other positions may be tolerated more flexibly [32,37]. This mechanistic framework may help explain our observation that all cyclic products retained the same cyclization pattern, in which EndC_TE catalyzed lactone formation between the second residue Thr and the C-terminal residue of each engineered intermediate as observed in enduracidin. Together, these findings suggest that EndC_TE maintains its regioselectivity across substrates of different lengths.

T–TE interactions have also been proposed to enhance TE-catalyzed cyclization in vitro. Previous work on the RufT PCP–TE didomain showed that the adjacent PCP domain enhanced cyclization efficiency compared with the TE monodomain, and the authors proposed that this effect may result from changes in lid-domain conformation and reduced water access to the active site [27]. In addition, TEs functioning in vivo within the intact NRPS assembly line depend on interaction with the upstream T domain for efficient intermediate transfer [30,31]. Such T–TE interactions may also contribute to the differences in product levels among the engineered strains.

Notably, En-M10 produced no detectable cyclic or linear products. Unlike the other productive constructs, this design relocated EndC_TE to the first module of EndC and generated the shortest truncated EndC variant, reducing its molecular weight from 928.74 kDa to 137.48 kDa. Previous studies have shown that intermediate transfer in multimodular NRPS systems is highly sensitive to local domain context [61], intersubunit communication [62,63], and the compatibility of engineered domain interfaces [31]. Accordingly, the lack of detectable products of En-M10 is more likely to reflect impaired assembly-line function than a simple shift in TE-mediated release mode. In this context, the substantial truncation of EndC, together with introduction of a non-native T10–TE junction, may have compromised transfer from EndB to EndC and/or impaired productive off-loading by the relocated TE.

In the present study, we retained the native T17–EndC_TE linker in all engineered assembly lines, and several constructs successfully produced the expected products, indicating that this linker-preserving design is feasible in the enduracidin system. However, when non-native terminal TE domains are used, employing a more complete functional exchange unit, as exemplified by the T domain-based exchange unit (XUT) [8], such as a T-TE didomain, may be more favorable for maintaining assembly-line activity. Previous studies have shown that the evolved PCP domains can restore effective interactions with TEs [64]. In addition, a recent study on polyketide synthase (PKS) engineering has likewise highlighted that the carrier-domain–TE didomain insertion is more effective for product release [65]. Together, these observations suggest that future TE relocation designs, especially those involving heterologous TE domains, may benefit from fusion sites that better preserve native T–TE relationships.

Although the present work demonstrates the feasibility of TE relocation in the large and complex enduracidin NRPS assembly line, its applicability to other NRPS systems remains to be further explored. Future studies in additional NRPS systems will help further refine this engineering strategy, evaluate its broader applicability, and improve our understanding of T–TE compatibility and interdomain interactions.

In this study, we identified an uncommon tri-TE architecture in the enduracidin biosynthetic gene cluster, and proposed a division of labor among three TEs that collectively maintains the efficiency and fidelity of enduracidin biosynthesis, as illustrated in the proposed pathway model in Fig. S9. We further demonstrated the feasibility of TE relocation in the enduracidin system and showed that EndC_TE exhibits broad substrate-length tolerance together with stringent regioselectivity. HRMS/MS analyses supported a conserved macrocyclization mode among the engineered cyclic derivatives, consistent with that of the wild-type cyclic product End_CA. Although further structural confirmation by NMR would be desirable, we were unable to obtain the material of sufficient purity and quantity for reliable NMR analysis. In particular, the presence of closely related homologs, together with substantial sample loss during repeated preparative purification and desalting, prevented the isolation of suitable material. Nevertheless, the consistent HRMS/MS fragmentation patterns, together with systematic comparison to wild-type End_CA, provided strong support for the proposed cyclization-site assignments. Overall, this study improves our understanding of TE domain function in NRPS engineering and provides a practical example of TE relocation in a large NRPS system, particularly with respect to substrate-length tolerance and regioselectivity.

## Ethics approval

This article does not contain any studies with human participants or experimental animals performed by any of the authors.

## Funding

This work was supported by the Beijing-Tianjin-Hebei Natural Science Foundation Cooperative Special Program (25JJJJC0023).

## CRediT authorship contribution statement

**Yanan Sun:** Data curation, Formal analysis, Investigation, Visualization, Writing – original draft. **Guibin Tu:** Formal analysis, Validation. **Xiaocan Sun:** Formal analysis, Validation. **Hanchao Zhang:** Formal analysis, Validation. **Haikuan Wang:** Conceptualization, Supervision, Writing – review & editing. **Fufeng Liu:** Conceptualization, Formal analysis, Supervision. **Fuping Lu:** Conceptualization, Project administration, Resources, Supervision. **Huitu Zhang:** Conceptualization, Funding acquisition, Project administration, Resources, Supervision, Writing – review & editing.

## Declaration of competing interest

The authors declare that they have no known competing financial interests or personal relationships that could have appeared to influence the work reported in this paper.

## Data Availability

Data will be made available on request.
