# Supplementary material for: Reprogramming the enduracidin NRPS assembly line via thioesterase domain relocation
Source: Synth Syst Biotechnol. 2026 Jun 24;15:72–85. doi: 10.1016/j.synbio.2026.05.013 (PMC13320435; doi:10.1016/j.synbio.2026.05.013)
Supplement: Multimedia component 1 [file mmc1.docx]

**Supplementary data**

**Author Affiliations**

Key Laboratory of Industrial Fermentation Microbiology, College of Biotechnology, Tianjin University of Science & Technology, Tianjin, 300457, PR China

**^🖂^ Corresponding author:** Huitu Zhang

**Mailing address:** No. 29, 13 Main Street, Tianjin Economic and Technological Development Area, Tianjin 300457, PR China

**Phone:** +86-22-60601958

**Fax:** +86-22-60600810

**E-mail:** [hzhang@tust.edu.cn](mailto:hzhang@tust.edu.cn)

Table of Contents

[Supplementary Tables 3](#_Toc227942465)

[**Table S1** The strains used in this study 3](#_Toc227942466)

[**Table S2** The plasmids used in this study 5](#_Toc227942467)

[**Table S3** The primers used in this study 7](#_Toc227942468)

[**Table S4** Expression levels (TPM) of genes in the enduracidin gene cluster of *Streptomyces fungicidicus* TXX3120 at different time points. 15](#_Toc227942469)

[**Table S5** Structural alignment parameters between End_25620 and homologous proteins 17](#_Toc227942470)

[**Table S6** Structural alignment parameters between End_25645 and homologous proteins 17](#_Toc227942471)

[**Table S7** Structural alignment parameters between EndC_TE and homologous proteins 18](#_Toc227942472)

[**Table S8** Gene IDs, protein numbers, and annotations of the enduracidin biosynthetic gene cluster 19](#_Toc227942473)

[**Table S9** Theoretical and observed *m/z* values of enduracidin and its derivatives at different charge states identified via the Find-by-Formula algorithm 20](#_Toc227942474)

[**Table S10** Theoretical and observed *m/z* values of parent and fragment ions of End_C9. 21](#_Toc227942475)

[**Table S11** Theoretical and observed *m/z* values of parent and fragment ions of End_C11. 22](#_Toc227942476)

[**Table S12** Theoretical and observed *m/z* values of parent and fragment ions of End_L11. 23](#_Toc227942477)

[**Table S13** Theoretical and observed *m/z* values of parent and fragment ions of End_C12. 24](#_Toc227942478)

[**Table S14** Theoretical and observed *m/z* values of parent and fragment ions of End_L12. 25](#_Toc227942479)

[**Table S15** Theoretical and observed *m/z* values of parent and fragment ions of End_C13. 26](#_Toc227942480)

[**Table S16** Theoretical and observed *m/z* values of parent and fragment ions of End_L13. 27](#_Toc227942481)

[**Table S17** Theoretical and observed *m/z* values of parent and fragment ions of End_C14. 28](#_Toc227942482)

[**Table S18** Theoretical and observed *m/z* values of parent and fragment ions of End_L14. 29](#_Toc227942483)

[**Table S19** Theoretical and observed *m/z* values of parent and fragment ions of End_C15. 30](#_Toc227942484)

[**Table S20** Theoretical and observed *m/z* values of parent and fragment ions of End_L15. 31](#_Toc227942485)

[**Table S21** Theoretical and observed *m/z* values of parent and fragment ions of End_C16. 32](#_Toc227942486)

[**Table S22** Theoretical and observed *m/z* values of parent and fragment ions of End_CA. 33](#_Toc227942487)

[**Table S23** Theoretical and observed *m/z* values of parent and fragment ions of End_LA. 34](#_Toc227942488)

[**Table S24** Details of the TEs included in the phylogenetic analysis (Figure 1E). 36](#_Toc227942489)

[Supplementary Figures 37](#_Toc227942490)

[**Figure S1** Expression levels (TPM) of genes in the enduracidin gene cluster of *Streptomyces fungicidicus* TXX3120 at different time points 37](#_Toc227942491)

[**Figure S2** Conservation analysis of amino acid residues in three TEs. 39](#_Toc227942492)

[**Figure S3** Conservation of the catalytic triad in three TEs. 40](#_Toc227942493)

[**Figure S4** Predicted aligned error (PAE) maps and predicted template modeling (pTM) scores of the three TEs predicted by AlphaFold3. 41](#_Toc227942494)

[**Figure S5** Comparative gene cluster, structural, and confidence analysis of End_25645 with related TEIIs from ramoplanin and WS9326 biosynthetic gene clusters. 42](#_Toc227942495)

[**Figure S6** Comparative gene cluster, structural, and confidence analysis of End_25620 with related TEIIs from biosynthetic gene clusters. 43](#_Toc227942496)

[**Figure S7** Structural and confidence analysis of EndC_TE and related TEIs from biosynthetic gene clusters. 44](#_Toc227942497)

[**Figure S8** Chemical structures of representative products from diverse NRPS biosynthetic gene clusters. 45](#_Toc227942498)

[**Figure S9** Proposed biosynthetic pathway of enduracidin. 46](#_Toc227942499)

[**Figure S10** Comparison of interatomic distances within the catalytic triads of End_25620 and the mutant S104A. 47](#_Toc227942500)

[**Figure S11** Comparison of interatomic distances within the catalytic triads of End_25645 and the mutant S79A. 48](#_Toc227942501)

[**Figure S12** Comparison of interatomic distances within the catalytic triads of EndC_TE and the mutant S8587A. 49](#_Toc227942502)

[**Figure S13** LC–HRMS and isotopic distribution analysis of End_C9. 50](#_Toc227942503)

[**Figure S14** LC–HRMS/MS spectrum of End_C9. 51](#_Toc227942504)

[**Figure S15** LC–HRMS spectrum and isotopic distribution analysis of End_L9. 52](#_Toc227942505)

[**Figure S16** LC–HRMS and isotopic distribution analysis of End_C11. 53](#_Toc227942506)

[**Figure S17** LC–HRMS and isotopic distribution analysis of End_L11. 54](#_Toc227942507)

[**Figure S18** LC–HRMS/MS spectra of End_C11 and End_L11. 55](#_Toc227942508)

[**Figure S19** LC–HRMS and isotopic distribution analysis of End_C12. 56](#_Toc227942509)

[**Figure S20** LC–HRMS and isotopic distribution analysis of End_L12. 57](#_Toc227942510)

[**Figure S21** LC–HRMS/MS spectra of End_C12 and End_L12. 58](#_Toc227942511)

[**Figure S22** LC–HRMS and isotopic distribution analysis of End_C13. 59](#_Toc227942512)

[**Figure S23** LC–HRMS and isotopic distribution analysis of End_L13. 60](#_Toc227942513)

[**Figure S24** LC–HRMS/MS spectra of End_C13 and End_L13. 61](#_Toc227942514)

[**Figure S25** LC–HRMS and isotopic distribution analysis of End_C14. 62](#_Toc227942515)

[**Figure S26** LC–HRMS and isotopic distribution analysis of End_L14. 63](#_Toc227942516)

[**Figure S27** LC–HRMS/MS spectra of End_C14 and End_L14. 64](#_Toc227942517)

[**Figure S28** LC–HRMS and isotopic distribution analysis of End_C15. 65](#_Toc227942518)

[**Figure S29** LC–HRMS and isotopic distribution analysis of End_L15. 66](#_Toc227942519)

[**Figure S30** LC–HRMS/MS spectra of End_C15 and End_L15. 67](#_Toc227942520)

[**Figure S31** LC–HRMS and isotopic distribution analysis of End_C16. 68](#_Toc227942521)

[**Figure S32** LC–HRMS and isotopic distribution analysis of End_L16. 69](#_Toc227942522)

[**Figure S33** LC–HRMS/MS spectrum of End_C16. 70](#_Toc227942523)

[**Figure S34** LC–HRMS and isotopic distribution analysis of End_CA. 71](#_Toc227942524)

[**Figure S35** LC–HRMS and isotopic distribution analysis of End_LA. 72](#_Toc227942525)

[**Figure S36** LC–HRMS/MS spectra of End_CA and End_LA. 73](#_Toc227942526)

[**Figure S37** HPLC–DAD analysis of the EndD (*CNQ36_25655*) in-frame deletion mutant and wild-type strain SFA. 74](#_Toc227942527)

[**Figure S38** Bioinformatic analysis of NRPS modules and domain organization in the enduracidin biosynthetic gene cluster. 75](#_Toc227942528)

[**Figure S39** HPLC–DAD and LC–HRMS analyses of the EndC_TE S8587A mutant and wild-type strain SFA. 76](#_Toc227942529)

[**Figure S40** Schematic illustration of the biosynthetic logic of enduracidin. 77](#_Toc227942530)

Supplementary Tables

**Table S1** The strains used in this study

| **Strains** | **Relevant characteristics** | **Sources** |
| --- | --- | --- |
| *E. coli* JM109 | Host for plasmid general cloning | Maintained in lab |
| *E. coli* ET12567/pUZ8002 | For conjugation transfer between *E. coli* and *Streptomyces* | Maintained in lab |
| *S. fungicidicus* TXX3120 | Wild type, industrial producer strain of enduracidin | Xinxing Veterinary Pharmaceutical Co.  (Tianjin, China). |
| *B. subtilis* CMCC (B) 63501 | Used for antimicrobial activity of enduracidin | Maintained in lab |
| SFA | Derivative of wild type *S.fungicidicus* TXX3120 with *upp* gene (*CNQ36_16770*) deletion | Maintained in lab |
| S104A | Derivative of SFA with a Ser to Ala mutation at position 104 of End_25620 | This work |
| S104A::stnYp1-25620 | Complemented derivative of S104A carrying the integrative plasmid pSET152-stnYp1-25620 | This work |
| △25620 | Derivative of SFA with an in-frame deletion of the gene *CNQ36_25620* | Maintained in lab |
| △25620::stnYp1-25620 | Complemented derivative of △25620 carrying the integrative plasmid pSET152-stnYp1-25620 | This work |
| S79A | Derivative of SFA with a Ser to Ala mutation at position 79 of End_25645 | This work |
| S79A::stnYp1-25645 | Complemented derivative of S79A carrying the integrative plasmid pSET152-stnYp1-25645 | This work |
| S8587A | Derivative of SFA with a Ser to Ala mutation at position 8587 of EndC | This work |
| S8587A::stnYp1-EndC_TE | Complemented derivative of S8587A carrying the integrative plasmid pSET152-stnYp1-EndC_TE | This work |
| SFA-M14 | NRPS assembly line engineered to terminate at module 14, with the terminal EndC_TE domain repositioned downstream to module 14 | This work |
| SFA-M15 | NRPS assembly line engineered to terminate at module 15, with the terminal EndC_TE domain repositioned downstream to module 15 | This work |
| SFA-M16 | NRPS assembly line engineered to terminate at module 16, with the terminal EndC_TE domain repositioned downstream to module 16 | This work |
| SFA-M9 | NRPS assembly line engineered to terminate at module 9, with the terminal EndC_TE domain repositioned downstream to module 9 | This work |
| SFA-M11 | NRPS assembly line engineered to terminate at module 11, with the terminal EndC_TE domain repositioned downstream to module 11 | This work |
| SFA-M12 | NRPS assembly line engineered to terminate at module 12, with the terminal EndC_TE domain repositioned downstream to module 12 | This work |
| SFA-M13 | NRPS assembly line engineered to terminate at module 13, with the terminal EndC_TE domain repositioned downstream to module 13 | This work |
| SFA-M10 | NRPS assembly line engineered to terminate at module 10, with the terminal EndC_TE domain repositioned downstream to module 10 | This work |

**Table S2** The plasmids used in this study

| **Plasmids** | **Relevant characteristics** | **Sources** |
| --- | --- | --- |
| pKC1139-*upp* | Derived from pKC1139, gene editing vector for traditional HR method with the *upp* counterselection marker | Maintained in lab |
| pKC1139-620S104A | pKC1139-*upp* containing two homologous arms (HA) and mutation sites for *CNQ36_25620* | This work |
| pKC1139-640S8587A | pKC1139-*upp* containing two HAs and mutation sites for *CNQ36_25640* | This work |
| pKC1139-645S79A | pKC1139-*upp* containing two HAs and mutation sites for *CNQ36_25645* | This work |
| pSET152 | Used for gene complementation or overexpression in *Streptomyces* | Maintained in lab |
| P1 | Vector containing the stnYp1 promoter, commercially synthesized | Maintained in lab |
| pSET152-stnYp1-25620 | pSET152 containing gene *CNQ36_25620* under the control of the promoter stnYp1. | This work |
| pSET152-stnYp1-25645 | pSET152 containing gene *CNQ36_25645* under the control of the promoter stnYp1. | This work |
| pSET152-stnYp1-EndC_TE | pSET152 containing gene *EndC_TE* under the control of the promoter stnYp1. | This work |
| pCas9-*upp* | pCas9 containing reverse screening marker *upp*, used for gene editing | Maintained in lab |
| pCas9-*upp-*M14-sg1 | pCas9-*upp-*M14 containing two HAs and sgRNA1 | This work |
| pCas9-*upp-*M14-sg2 | pCas9-*upp-*M14 containing two HAs and sgRNA2 | This work |
| pCas9-*upp-*M14-sg3 | pCas9-*upp-*M14 containing two HAs and sgRNA3 | This work |
| pCas9-*upp-*M15-sg1 | pCas9-*upp-*M15 containing two HAs and sgRNA1 | This work |
| pCas9-*upp-*M15-sg2 | pCas9-*upp-*M15 containing two HAs and sgRNA2 | This work |
| pCas9-*upp-*M15-sg3 | pCas9-*upp-*M15 containing two HAs and sgRNA3 | This work |
| pCas9-*upp-*M16-sg1 | pCas9-*upp-*M16 containing two HAs and sgRNA1 | This work |
| pCas9-*upp-*M16-sg2 | pCas9-*upp-*M16 containing two HAs and sgRNA2 | This work |
| pCas9-*upp-*M16-sg3 | pCas9-*upp-*M16 containing two HAs and sgRNA3 | This work |
| pCas9-*upp-*M9-sg1 | pCas9-*upp-*M9 containing two HAs and sgRNA1 | This work |
| pCas9-*upp-*M9-sg2 | pCas9-*upp-*M9 containing two HAs and sgRNA2 | This work |
| pCas9-*upp-*M9-sg3 | pCas9-*upp-*M9 containing two HAs and sgRNA3 | This work |
| pCas9-*upp-*M11-sg1 | pCas9-*upp-*M11 containing two HAs and sgRNA1 | This work |
| pCas9-*upp-*M11-sg2 | pCas9-*upp-*M11 containing two HAs and sgRNA2 | This work |
| pCas9-*upp-*M11-sg3 | pCas9-*upp-*M11 containing two HAs and sgRNA3 | This work |
| pCas9-*upp-*M12-sg1 | pCas9-*upp-*M12 containing two HAs and sgRNA1 | This work |
| pCas9-*upp-*M12-sg2 | pCas9-*upp-*M12 containing two HAs and sgRNA2 | This work |
| pCas9-*upp-*M12-sg3 | pCas9-*upp-*M12 containing two HAs and sgRNA3 | This work |
| pCas9-*upp-*M13-sg1 | pCas9-*upp-*M13 containing two HAs and sgRNA1 | This work |
| pCas9-*upp-*M13-sg2 | pCas9-*upp-*M13 containing two HAs and sgRNA2 | This work |
| pCas9-*upp-*M13-sg3 | pCas9-*upp-*M13 containing two HAs and sgRNA3 | This work |
| pCas9-*upp-*M10-sg1 | pCas9-*upp-*M10 containing two HAs and sgRNA1 | This work |
| pCas9-*upp-*M10-sg2 | pCas9-*upp-*M10 containing two HAs and sgRNA2 | This work |
| pCas9-*upp-*M10-sg3 | pCas9-*upp-*M10 containing two HAs and sgRNA3 | This work |

**Table S3** The primers used in this study

| **Primers** | **Sequences (5’ to 3’)** | **Functions** |
| --- | --- | --- |
| pKC*upp*-M13-cF | GTAACGCCAGGGTTTTCCCAGT | Sequencing primers targeting regions flanking the homology arms on the vector pKC1139-*upp* |
| pKC*upp*-ermE-cR | CGAACGGACACTCGCATGCAT |  |
| 152-upCAP_cF | GGCAGTGAGCGCAACGCAAT | Sequencing primers targeting regions on the vector pSET152 |
| 152-upCAP_cF2 | GAGCGCAGCGAGTCAGTGAGC |  |
| 152-donm13f-cR | GGGATGTGCTGCAAGGCGATT |  |
| S104A-inUP-tF | CCGTGGCCGCGTGCGATAGT | Sequencing primers for HAs for TE domains mutation |
| S8587A-inUP-tF | CGTCGGCGTCGACGACGACT |  |
| S79A-inUP-tF | GGCCTCGTTCTTCCACCCGT |  |
| pkc620_S104A-Hindiii-upF | GTAAAACGACGGCCAGTGCCAAGCTTCGAGTCCAGTCCGTAGTGCG | Amplification of HAs containing mutation sites for *CNQ36_25620* |
| pkc620_S104A-upR | TCGCCCCCAGCGCGGTGCCCACG |  |
| pkc620_S104A-downF2 | ATCGTGGGCACCGCGCTGGG |  |
| pkc620_S104A-Xbai-downR2 | CGCGCGGCCGCGGATCCTCTAGAGAGGCCGAACACGACGGACGT |  |
| pkc645_S79A-Hindiii-upF | GTAAAACGACGGCCAGTGCCAAGCTTCGACTACATCGAGCAGCTGCGC | Amplification of HAs containing mutation sites for *CNQ36_25645* |
| pkc645_S79A-upR | CACCGCGCCGAGCGCGTGCCCGAAGAT |  |
| pkc645_S79A-downF | ATCTTCGGGCACGCGCTCGGCGCGGT |  |
| pkc645_S79A-XbaI-downR | CGCGCGGCCGCGGATCCTCTAGACGTTGTTGAGCAGCACGTCGAT |  |
| pkc640_S8587A-Hindiii-upF | GTAAAACGACGGCCAGTGCCAAGCTTCGCAGTACGAGGTCGCCGATG | Amplification of HAs containing mutation sites for *CNQ36_25640* |
| pkc640_S8587A-upR2 | CGCGCCGAAGGCGTATCCGACGAG |  |
| pkc640_S8587A-downF | CTCGTCGGATACGCCTTCGGCGCG |  |
| pkc640_S8587A-XbaI-downR2 | CGCGCGGCCGCGGATCCTCTAGAGTCTGCTCGGCCGTGACCAGGT |  |
| Kno25620-cF2 | GGATGAAGAACAGCGGGATCAT | PCR primers for genomic fragment containing mutation sites for *CNQ36_25620*, or validation for the deletion of *CNQ36_25620* |
| Kno25620-cR2 | GATCTCCACGTACTCGGCGAT |  |
| MS79A-tF | CACATCCGTCCGGGACCTGAT | PCR primers for genomic fragment containing mutation sites for *CNQ36_25645* |
| MS79A-tR | GCGAAGTTGCGGTATCCGACCT |  |
| MS8587A-tF | GACCTGTCCCATGTGCATGT | PCR primers for genomic fragment containing mutation sites for *CNQ36_25640* |
| MS8587A-tR | GTCCTCGGCGATGTACATGT |  |
| TS104A-228F | GGCGAACCGGTGCTCATGAT | Primers for sequencing fragments containing mutation sites |
| TS79A-242F | GAACAGACATGCGACGGACGT |  |
| TS8587A-255F | CTCGGCGTGCTGCTGACGAT |  |
| p152-stnYp1-rbs-F | ACAGCTATGACATGATTACGAATTCTGTGCGAGCATAACCTCTGCC | PCR primers for the promoter stnYp1 fragment |
| p152-stnYp-rbs-R | TCCTGTGGAGTGGTTCTGTCTTAAGCTCTTCAGCAGTCATGTGAGCAT |  |
| P152-25620-F | CCACTCCACAGGAGGACCCATATGGACCACACCGCGGGCCG | PCR primers for the gene *CNQ36_25620* fragment |
| P152-25620-R | GGGCTGCAGGTCGACTCTAGATTAGGCGTCGCGTCAGTGCTTGTCG |  |
| P152-79-F | CCACTCCACAGGAGGACCCATATGCGACGGACGTCTCTGCT | PCR primers for the gene *CNQ36_25645* fragment |
| P152-79-R | GGGCTGCAGGTCGACTCTAGATTACTACCGGGCGAGCGCGTCGT |  |
| P152-8587-F | CCACTCCACAGGAGGACCCATATGGAACAGGCCATGTGCGAGGC | PCR primers for the TE domain of EndC (encoded within gene *CNQ36_25640*) |
| P152-8587-R | GGGCTGCAGGTCGACTCTAGATTATCAACGGGACTTCAGCCAGGC |  |
| P_cap_xbaI-te1+2-14up-F | CAAGCTTGGGCTGCAGGTCGACTCTAGA GTCGCCGAGGACGCGGACGT | Amplification of upstream HA used for relocating the TE domain to module 14 |
| P_cap_xbaI-te1+2-14up-R | GACAGGCCCAGCGTGCCGCGCGCCGCTCCGGAAGCGGCC |  |
| P_cap_xbaI-te1+2-15up-F | CAAGCTTGGGCTGCAGGTCGACTCTAGA TTCCTGCTGGAGGAGCGCGT | Amplification of upstream HA used for relocating the TE domain to module 15 |
| P_cap_xbaI-te1+2-15up-R | GACAGGCCCAGCGTGCCGCGCGCGGCGTGAGCGAGTCCG |  |
| P_cap_xbaI-te1+2-16up-F | CAAGCTTGGGCTGCAGGTCGACTCTAGAGGTTCGGGGCGAGCCTGATGCAG | Amplification of upstream HA used for relocating the TE domain to module 16 |
| P_cap_xbaI-te1+2-16up-R | GACAGGCCCAGCGTGCCGCGCGTCTCCGGTCCCGCGGCC |  |
| P_cap_xbaI-te1+2-ndown-F | CGCGGCACGCTGGGCCTGTC | Amplification of downstream HA used for relocating the TE domain |
| P_cap_xbaI-te1+2-ndown-R | TCCCAACAGTTGCGCAGCTCTAGA GAGCACCGCGCCGAGGCTGT |  |
| TE1+2-indown-cR | CGCGCCCTCCCACGACAGGT | Primers for sequencing HAs used for TE domain relocation |
| cap-ud2F | CCAGAGCATCACCGGCCTGT | Sequencing primers for HAs on the vector pCas9-*upp* |
| cap-ud3R | CGACGTGGAGACGCATGTCGT |  |
| TE1+2-14Gly-sgRNA1-F | CGGTTGGTAGGATCGACGGCTGTCGAGTTGAGTTATGCGGGTTTTAGAGCTAGAAATAGC | Annealing to form dsDNA for sgRNA insertion, used for TE domain relocation to module 14 |
| TE1+2-14Gly-sgRNA1-R | GCTATTTCTAGCTCTAAAACCCGCATAACTCAACTCGACAGCCGTCGATCCTACCAACCG |  |
| TE1+2-14Gly-sgRNA2-F | CGGTTGGTAGGATCGACGGCGCCTGCCGTCACATGCACATGTTTTAGAGCTAGAAATAGC |  |
| TE1+2-14Gly-sgRNA2-R | GCTATTTCTAGCTCTAAAACATGTGCATGTGACGGCAGGCGCCGTCGATCCTACCAACCG |  |
| TE1+2-14Gly-sgRNA3-F | CGGTTGGTAGGATCGACGGCCCGGCCGGACAACTGCTGAGGTTTTAGAGCTAGAAATAGC |  |
| TE1+2-14Gly-sgRNA3-R | GCTATTTCTAGCTCTAAAACCTCAGCAGTTGTCCGGCCGGGCCGTCGATCCTACCAACCG |  |
| TE1+2-15End-sgRNA1-F | CGGTTGGTAGGATCGACGGCCGGTCGCGAACAGCCAGGCTGTTTTAGAGCTAGAAATAGC | Annealing to form dsDNA for sgRNA insertion, used for TE domain relocation to module 15 |
| TE1+2-15End-sgRNA1-R | GCTATTTCTAGCTCTAAAACAGCCTGGCTGTTCGCGACCGGCCGTCGATCCTACCAACCG |  |
| TE1+2-15End-sgRNA2-F | CGGTTGGTAGGATCGACGGCCCACGAAACGCTCACTGGTCGTTTTAGAGCTAGAAATAGC |  |
| TE1+2-15End-sgRNA2-R | GCTATTTCTAGCTCTAAAACGACCAGTGAGCGTTTCGTGGGCCGTCGATCCTACCAACCG |  |
| TE1+2-15End-sgRNA3-F | CGGTTGGTAGGATCGACGGCCACCCCGGAGATACTGACCCGTTTTAGAGCTAGAAATAGC |  |
| TE1+2-15End-sgRNA3-R | GCTATTTCTAGCTCTAAAACGGGTCAGTATCTCCGGGGTGGCCGTCGATCCTACCAACCG |  |
| TE1+2-16Ala-sgRNA1-F | CGGTTGGTAGGATCGACGGCGGACGTGTACGTGACACCCAGTTTTAGAGCTAGAAATAGC | Annealing to form dsDNA for sgRNA insertion, used for TE domain relocation to module 16 |
| TE1+2-16Ala-sgRNA1-R | GCTATTTCTAGCTCTAAAACTGGGTGTCACGTACACGTCCGCCGTCGATCCTACCAACCG |  |
| TE1+2-16Ala-sgRNA2-F | CGGTTGGTAGGATCGACGGCTCGACCAGGGTCAGTATCTCGTTTTAGAGCTAGAAATAGC |  |
| TE1+2-16Ala-sgRNA2-R | GCTATTTCTAGCTCTAAAACGAGATACTGACCCTGGTCGAGCCGTCGATCCTACCAACCG |  |
| TE1+2-16Ala-sgRNA3-F | CGGTTGGTAGGATCGACGGCCAGGGTGTTGATGAACAGTCGTTTTAGAGCTAGAAATAGC |  |
| TE1+2-16Ala-sgRNA3-R | GCTATTTCTAGCTCTAAAACGACTGTTCATCAACACCCTGGCCGTCGATCCTACCAACCG |  |
| cb-mcsF | GAGCGTCGATTTTTGTGATGC | Sequencing primers for the sgRNA insertion of pCas9-*upp* |
| M14-tF | TCCTGGAGCTCTATGTGCCGCT | PCR validation primers flanking the genomic homology arms, used for TE domain relocation to module 14, 15 and 16 |
| M15-tF | GTTCGACGCGTTCTGTGAGGAGT |  |
| M16-tF | GTGACGACGGTGACCTTCGACAT |  |
| M1n-tR | CTCCTTGACGCGCAGCAGGAACT |  |
| M14-t2F | GGGACCGGAACACCTCGTCGT |  |
| M15-t2F | CTTCCTGACGGAGACCGCCGAT |  |
| M16-t2F | CGTCGCCGAACTCGCGGATCT |  |
| M14-t3F | GGCGTCGGACCGGAGTCGT |  |
| M1n-t3R | TCGGCCGTGACCAGGTCGT |  |
| P_xcap_xbaI-t5620up-F | CAAGCTTGGGCTGCAGGTCGACTCTAGAGTCCTCCCCCTCGAAGGCCCT | Amplification of HAs of the gene *CNQ36_25620* |
| P_xca-t5620up-R | CTGTGTGTCGTCGGGGCGTCGCCCGCCTCTCCCTCCTCGTCGT |  |
| P_xca-t5620down-F | ACGACGAGGAGGGAGAGGCGGGCGACGCCCCGACGACACACAGCG |  |
| P_xcap_xbaI-t5620down-R | TCCCAACAGTTGCGCAGCTCTAGAAGCAGCGAGGTGAGGGCCT |  |
| xcas-5620-sgRNA78-F | CGGTTGGTAGGATCGACGGCTCATGAGCACCGGTTCGCCCGTTTTAGAGCTAGAAATAGC | Annealing to form dsDNA for sgRNA insertion, used for deletion of the gene *CNQ36_25620* |
| xcas-5620-sgRNA78-R | GCTATTTCTAGCTCTAAAACGGGCGAACCGGTGCTCATGAGCCGTCGATCCTACCAACCG |  |
| xcas-5620-sgRNA123-F | CGGTTGGTAGGATCGACGGCTCTGGTGCAGGGTCCAGGCAGTTTTAGAGCTAGAAATAGC |  |
| xcas-5620-sgRNA123-R | GCTATTTCTAGCTCTAAAACTGCCTGGACCCTGCACCAGAGCCGTCGATCCTACCAACCG |  |
| xcas-5620-sgRNA275-F | CGGTTGGTAGGATCGACGGCCCCTGGATCTGGCGCCGTGCGTTTTAGAGCTAGAAATAGC |  |
| xcas-5620-sgRNA275-R | GCTATTTCTAGCTCTAAAACGCACGGCGCCAGATCCAGGGGCCGTCGATCCTACCAACCG |  |
| P_cap_xbaI-te1+2-11up-F | CAAGCTTGGGCTGCAGGTCGACTCTAGAGGCACCGTGGTGATCGCGC | Amplification of upstream HA used for relocating the TE domain to module 11 |
| P_cap_xbaI-te1+2-11up-R | GACAGGCCCAGCGTGCCGCGTACGGCGGTCTCGGTCAGCC |  |
| P_cap_xbaI-te1+2-M9up-F | CAAGCTTGGGCTGCAGGTCGACTCTAGATGGCTGCTCGCACTGCTGC | Amplification of upstream HA used for relocating the TE domain to module 9 |
| P_cap_xbaI-te1+2-M9up-R | GACAGGCCCAGCGTGCCGCGCGCCGTCAGCCACTCGGC |  |
| P_cap_xbaI-te1+2-12up-F | CAAGCTTGGGCTGCAGGTCGACTCTAGATGCTGCACGGCGGACGCCTG | Amplification of upstream HA used for relocating the TE domain to module 12 |
| P_cap_xbaI-te1+2-12up-R | GACAGGCCCAGCGTGCCGCGCGCGGCCGCGACGGCGGCCA |  |
| P_cap_xbaI-te1+2-13up-F | CAAGCTTGGGCTGCAGGTCGACTCTAGACCGGCGGCACGGTCGTCAT | Amplification of upstream HA used for relocating the TE domain to module 13 |
| P_cap_xbaI-te1+2-13up-R | GACAGGCCCAGCGTGCCGCGGGCCTGGACGAGCCAGCGC |  |
| P_cap_xbaI-te1+2-10up-F | CAAGCTTGGGCTGCAGGTCGACTCTAGAGTGCGGTTCCTGCTGGAGGA | Amplification of upstream HA used for relocating the TE domain to module 10 |
| P_cap_xbaI-te1+2-10up-R | GACAGGCCCAGCGTGCCGCGGGCCGCCGCCGCCAGCGCG |  |
| M9up-c1F | CGAACTCCACCTCGCCGGCT | Primers for sequencing HAs used for TE domain relocation to module 9, 10, 11, 12 and 13 |
| M9up-c2F | TCGTCAGTGCGGGACTCGCT |  |
| M11up-c1F | CTACGCCAACCGCGCCGAGT |  |
| M12up-c1F | CGCAGGCCGCGCGCGGCTAT |  |
| M13up-c1F | TACGTAGCCGGTGCCGGCGT |  |
| M10up-c1F | GCGGATGTACCGCACCGGT |  |
| TE-M25640-sgRNA1-F_x1213 | CGGTTGGTAGGATCGACGGCGCCAGCGGAAGGTTGTACGTGTTTTAGAGCTAGAAATAGC | Annealing to form dsDNA for sgRNA insertion, used for TE domain relocation to module 9–13. Among them, sgRNA1 is not suitable for TE domain relocation to module 12 and 13. |
| TE-M25640-sgRNA1-R_x1213 | GCTATTTCTAGCTCTTAAAACACGTACAACCTTCCGCTGGCGCCGTCGCGCCGTCGATCCTACCAACCG |  |
| TE-M25640-sgRNA2-F | CGGTTGGTAGGATCGACGGCGCCTGCCGTCACATGCACATGTTTTAGAGCTAGAAATAGC |  |
| TE-M25640-sgRNA2-R | GCTATTTCTAGCTCTAAAACATGTGCATGTGACGGCAGGCGCCGTCGATCCTACCAACCG |  |
| TE-M25640-sgRNA3-F | CGGTTGGTAGGATCGACGGCCAGGGTGTTGATGAACAGTCGTTTTAGAGCTAGAAATAGC |  |
| TE-M25640-sgRNA3-R | GCTATTTCTAGCTCTAAAACGACTGTTCATCAACACCCTGGCCGTCGATCCTACCAACCG |  |
| TE-M25640-sgRNA4-F | CGGTTGGTAGGATCGACGGCTGTCGAGTTGAGTTATGCGGGTTTTAGAGCTAGAAATAGC |  |
| TE-M25640-sgRNA4-R | GCTATTTCTAGCTCTAAAACCCGCATAACTCAACTCGACAGCCGTCGATCCTACCAACCG |  |
| M9up-tF | GCGTCCGTCGGCTTCGACAT | PCR validation primers flanking the genomic homology arms, used for TE domain relocation |
| M9up-tF2 | GTCATCGACGACCCCGACTTCCT |  |
| M11up-tF | GACGCCTCCTGCTACGAGCT |  |
| M11up-tF2 | GACGGACACCGTGGTGGACCT |  |
| M12up-tF | CGCGTTCGACGTCTCCGTAT |  |
| M12up-tF2 | GCCTTCGGAGCAGACGACGT |  |
| M13up-tF | GACGCCTCGACGTACGAGCT |  |
| M13up-tF2 | AGGCGGCGTACGTGATGTACACAT |  |
| Mndown-tR | CAGCAGGTGCGCGAGTTCGT |  |
| Mndown-tR2 | GCGCAGCAGGAACTCCTCGT |  |
| M13up-tF3 | GGCCTCGGCATTCCGACACTCT |  |
| Mndown-tR3 | AGCTCGCGCATGTCCGGATG |  |
| M10up-tF | CGACGCGTTCTGCGAGGAGT |  |
| M10up-tF2 | TGTGGCGAGTCATGTCCGGT |  |

**Table S4** Expression levels (TPM) of genes in the enduracidin gene cluster of *Streptomyces fungicidicus* TXX3120 at different time points.

| **Gene ID** | **Expression level (TPM)** | | | | | | | | | | | | | | |
| --- | --- | --- | --- | --- | --- | --- | --- | --- | --- | --- | --- | --- | --- | --- | --- |
|  | 32 h | | | 48 h | | | | 72 h | | | | 144 h | | | |
|  | R2-2 | R2-1 | | R4-1 | | R4-2 | | R5-1 | | R5-2 | | R6-1 | | R6-2 | |
| \| *CNQ36_25560* \| \| --- \| \| *CNQ36_25565* \| \| *CNQ36_25570* \| \| *CNQ36_25575* \| \| *CNQ36_25580* \| \| *CNQ36_25585* \| \| *CNQ36_25590* \| \| *CNQ36_25595* \| \| *CNQ36_25600* \| \| *CNQ36_25605* \| \| *CNQ36_25610* \| \| *CNQ36_25615* \| \| *CNQ36_25620* \| \| *CNQ36_25625* \| \| *CNQ36_25630* \| \| *CNQ36_25635* \| \| *CNQ36_25640* \| \| *CNQ36_25645* \| \| *CNQ36_25650* \| \| *CNQ36_25655* \| \| *CNQ36_25660* \| \| *CNQ36_25665* \| \| *CNQ36_25670* \| \| *CNQ36_25675* \| \| *CNQ36_25680* \| \| *CNQ36_25685* \| \| *CNQ36_25690* \| | 10.627019 | | 11.214493 | | 332.7973 | | 287.74146 | | 325.09229 | | 546.72382 | | 880.19006 | | 940.429871 |
|  | 9.213732 | | 11.155021 | | 189.26137 | | 165.45914 | | 185.53336 | | 283.63748 | | 84.872643 | | 120.473106 |
|  | 5.471091 | | 29.951546 | | 345.96997 | | 222.30794 | | 275.5018 | | 514.2467 | | 140.15131 | | 125.096001 |
|  | 2.513077 | | 9.863022 | | 298.58942 | | 357.84 | | 306.46207 | | 853.16943 | | 178.19161 | | 175.450943 |
|  | 4.639999 | | 0 | | 66.06073 | | 53.97015 | | 109.3896 | | 149.28754 | | 27.09841 | | 41.264881 |
|  | 0.475626 | | 0 | | 170.03543 | | 168.53288 | | 177.46944 | | 7.653848 | | 1.135959 | | 9.617417 |
|  | 2.176401 | | 18.570839 | | 123.63009 | | 142.94127 | | 119.01989 | | 257.22427 | | 65.050224 | | 73.0326 |
|  | 3.935214 | | 13.035502 | | 202.64838 | | 158.95081 | | 147.19247 | | 302.68552 | | 82.300583 | | 91.664742 |
|  | 13.009603 | | 20.035074 | | 460.53403 | | 435.55444 | | 390.76123 | | 1208.0342 | | 335.23557 | | 334.96524 |
|  | 21.177471 | | 57.192226 | | 962.85565 | | 450.59271 | | 381.37607 | | 711.87958 | | 1362.28 | | 1594.18237 |
|  | 1.846842 | | 34.467945 | | 14.776981 | | 7.760389 | | 340.10864 | | 605.26288 | | 1480.5427 | | 1502.07166 |
|  | 3.628966 | | 10.962452 | | 153.58051 | | 112.44106 | | 64.295311 | | 227.71964 | | 24.188993 | | 23.808092 |
|  | 13.581071 | | 20.731144 | | 1842.5093 | | 1646.6727 | | 967.93701 | | 1360.1613 | | 309.43478 | | 400.743683 |
|  | 0 | | 0 | | 185.94579 | | 284.37122 | | 188.6373 | | 333.04886 | | 25.235107 | | 9.478438 |
|  | 6.082912 | | 2.370303 | | 176.31923 | | 190.34283 | | 150.20917 | | 263.65173 | | 30.931007 | | 32.638607 |
|  | 5.088231 | | 0 | | 200.56357 | | 37.502777 | | 133.42116 | | 285.237 | | 75.805405 | | 7.46666 |
|  | 1.490336 | | 12.016882 | | 44.106602 | | 39.69487 | | 47.955727 | | 2.522943 | | 22.811783 | | 8.185555 |
|  | 3.645404 | | 7.598986 | | 314.18692 | | 145.13141 | | 126.97286 | | 414.18991 | | 46.244038 | | 71.136345 |
|  | 3.441842 | | 45.070133 | | 200.27914 | | 152.19658 | | 135.17354 | | 188.44026 | | 30.726086 | | 59.827774 |
|  | 2.910178 | | 6.313008 | | 145.17276 | | 117.1247 | | 106.25691 | | 193.23344 | | 44.241608 | | 57.526516 |
|  | 24.697897 | | 28.576149 | | 368.03781 | | 400.49365 | | 264.24167 | | 287.04321 | | 371.45392 | | 421.393646 |
|  | 0.89746 | | 3.114964 | | 16.382708 | | 293.01971 | | 244.45723 | | 427.5188 | | 659.2536 | | 788.120667 |
|  | 41.112469 | | 87.197487 | | 1418.4427 | | 915.36395 | | 605.08484 | | 613.40991 | | 4027.1399 | | 3723.82642 |
|  | 0.885798 | | 12.521597 | | 5.366202 | | 2.592642 | | 5.542051 | | 3.536508 | | 2.595995 | | 2.602988 |
|  | 7.107913 | | 6.049083 | | 438.11359 | | 372.65335 | | 315.64014 | | 371.92639 | | 153.6725 | | 145.883453 |
|  | 11.109687 | | 24.010262 | | 538.00537 | | 389.60391 | | 339.25272 | | 529.06702 | | 171.48163 | | 187.615631 |
|  | 31.773844 | | 45.236454 | | 665.77106 | | 455.48447 | | 650.15729 | | 709.14728 | | 212.16689 | | 182.379593 |

**Table S5** Structural alignment parameters between End_25620 and homologous proteins

| **Comparison** | **End_25620**  **vs**  **DptH** | **End_25620**  **vs**  **LptH** | **End_25620**  **vs**  **CdeB** | **End_25620**  **vs**  **Ram_12** |
| --- | --- | --- | --- | --- |
| Aligned residues | 262 | 257 | 261 | 264 |
| Aligned atoms | 217 | 220 | 216 | 222 |
| RMSD (Å) | 0.736 | 0.691 | 0.548 | 0.238 |
| MatchAlign score | 602.5 | 637 | 727 | 1067 |

**Table S6** Structural alignment parameters between End_25645 and homologous proteins

| **Comparison** | **End_25645**  **vs**  **WS20** | **End_25645**  **vs**  **Ram_18** | **End_25645**  **vs**  **WS5** | **WS20**  **vs**  **WS5** |
| --- | --- | --- | --- | --- |
| Aligned residues | 233 | 233 | 205 | 213 |
| Aligned atoms | 180 | 195 | 168 | 166 |
| RMSD (Å) | 0.438 | 0.696 | 2.44 | 1.688 |
| MatchAlign score | 714 | 781 | 203.5 | 255.5 |

**Table S7** Structural alignment parameters between EndC_TE and homologous proteins

| **Comparison** | **EndC_TE**  **vs**  **DptD_TE** | **EndC_TE**  **vs**  **LptD_TE** | **EndC_TE**  **vs**  **Ram17_TE** | **EndC_TE**  **vs**  **CdeK_TE** | **EndC_TE**  **vs**  **WS19_TE** |
| --- | --- | --- | --- | --- | --- |
| Aligned residues | 200 | 208 | 223 | 200 | 210 |
| Aligned atoms | 148 | 151 | 177 | 151 | 160 |
| RMSD (Å) | 1.037 | 1.001 | 0.852 | 0.86 | 1.044 |
| MatchAlign score | 356 | 328 | 746 | 356.5 | 364 |

**Table S8** Gene IDs, protein numbers, and annotations of the enduracidin biosynthetic gene cluster

| Gene ID | Protein Number  (user defined) | Gene description |
| --- | --- | --- |
| *CNQ36_25560* | End_25560 | transcriptional regulator |
| *CNQ36_25565* | End_25565 | prephenate dehydrogenase |
| *CNQ36_25570* | End_25570 | streptomycin biosynthesis protein |
| *CNQ36_25575* | End_25575 | 4-hydroxyphenylpyruvate dioxygenase |
| *CNQ36_25580* | End_25580 | enduracididine biosynthesis enzyme MppR |
| *CNQ36_25585* | End_25585 | enduracididine biosynthesis enzyme MppQ |
| *CNQ36_25590* | End_25590 | enduracididine biosynthesis enzyme MppP |
| *CNQ36_25595* | End_25595 | aminotransferase class I/II-fold pyridoxal phosphate-dependent enzyme |
| *CNQ36_25600* | End_25600 | tryptophan 7-halogenase |
| *CNQ36_25605* | End_25605 | ABC transporter permease subunit |
| *CNQ36_25610* | End_25610 | ABC transporter ATP-binding protein |
| *CNQ36_25615* | End_25615 | ABC transporter |
| *CNQ36_25620* | End_25620 | alpha/beta hydrolase |
| *CNQ36_25625* | End_25625 | acyl carrier protein |
| *CNQ36_25630* | EndA | non-ribosomal peptide synthetase |
| *CNQ36_25635* | EndB | non-ribosomal peptide synthetase |
| *CNQ36_25640* | EndC | non-ribosomal peptide synthetase |
| *CNQ36_25645* | End_25645 | thioesterase II family protein |
| *CNQ36_25650* | End_25650 | SDR family oxidoreductase |
| *CNQ36_25655* | EndD | amino acid adenylation domain-containing protein |
| *CNQ36_25660* | End_25660 | response regulator transcription factor /DNA-binding response regulator |
| *CNQ36_25665* | End_25665 | two-component sensor histidine kinase |
| *CNQ36_25670* | End_25670 | metal transporter |
| *CNQ36_25675* | End_25675 | acyl-CoA dehydrogenase |
| *CNQ36_25680* | End_25680 | acyl-CoA dehydrogenase family protein |
| *CNQ36_25685* | End_25685 | fatty acyl-AMP ligase |
| *CNQ36_25690* | End_25690 | MbtH family protein |
| *CNQ36_25695* | End_25695 | rhomboid family intramembrane serine protease |
| *CNQ36_25700* | End_25700 | hypothetical protein |
| *CNQ36_25705* | End_25705 | FAD-dependent oxidoreductase |
| *CNQ36_25710* | End_25710 | DUF4349 domain-containing protein |
| *CNQ36_25715* | End_25715 | protoporphyrinogen oxidase |
| *CNQ36_25720* | End_25720 | hydrogen peroxide-dependent heme synthase |

**Table S9** Theoretical and observed *m/z* values of enduracidin and its derivatives at different charge states identified via the Find-by-Formula algorithm

|  | Formula | [M+1H]^1+^ | | | [M+2H]^2+^ | | | [M+3H]^3+^ | | |
| --- | --- | --- | --- | --- | --- | --- | --- | --- | --- | --- |
|  |  | Calc. | Obs. | Err. | Calc. | Obs. | Err. | Calc. | Obs. | Err. |
| End_C9 | C_63_H_86_N_12_O_19_ | 1315.6205 | 1315.6227 | 1.6722 | 658.3139 | 658.3155 | 2.4305 | 439.2117 | - |  |
| End_L9 | C_63_H_88_N_12_O_20_ | 1333.6311 | - |  | 667.3192 | - |  | 445.2152 | - |  |
| End_C11 | C_77_H_103_N_17_O_22_ | 1618.7536 | 1618.7546 | 0.6178 | 809.8805 | 809.8820 | 1.8521 | 540.2561 | 540.2569 | 1.4808 |
| End_L11 | C_77_H_105_N_17_O_23_ | 1636.7642 | 1636.7680 | 2.3217 | 818.8857 | 818.8880 | 2.8087 | 546.2596 | 546.2611 | 2.7459 |
| End_C12 | C_80_H_108_N_18_O_24_ | 1705.7857 | 1705.7885 | 1.6415 | 853.3965 | 853.3978 | 1.5233 | 569.2667 | 569.2680 | 2.2836 |
| End_L12 | C_80_H_110_N_18_O_25_ | 1723.7962 | 1723.7993 | 1.7984 | 862.4018 | 862.4037 | 2.2031 | 575.2703 | 575.2714 | 1.9121 |
| End_C13 | C_88_H_113_Cl_2_N_19_O_26_ | 1922.7554 | 1922.7558 | 0.2080 | 961.8813 | 961.8835 | 2.2872 | 641.5900 | 641.5917 | 2.6497 |
| End_L13 | C_88_H_115_Cl_2_N_19_O_27_ | 1940.7660 | 1940.7691 | 1.5973 | 970.8866 | 970.8897 | 3.1930 | 647.5935 | 647.5948 | 2.0074 |
| End_C14 | C_90_H_116_N_20_O_27_Cl_2_ | 1979.7769 | 1979.7801 | 1.6163 | 990.3921 | 990.3947 | 2.6252 | 660.5971 | 660.5990 | 2.8762 |
| End_L14 | C_90_H_118_N_20_O_28_Cl_2_ | 1997.7874 | 1997.7793 | -4.0545 | 999.3974 | 999.3986- | 1.2007 | 666.6007 | 666.6026 | 2.8503 |
| End_C15 | C_96_H_126_N_24_O_28_Cl_2_ | 2133.8623 | - |  | 1067.4348 | 1067.4352 | 0.3747 | 711.9590 | 711.9605 | 2.1069 |
| End_L15 | C_96_H_128_N_24_O_29_Cl_2_ | 2151.8729 | - |  | 1076.4401 | 1076.4430 | 2.6941 | 717.9625 | 717.9650 | 3.4821 |
| End_C16 | C_99_H_131_N_25_O_29_Cl_2_ | 2204.8994 | 2204.9010 | 0.7257 | 1102.9534 | 1102.9554 | 1.8133 | 735.638 | 735.6395 | 2.0390 |
| End_L16 | C_99_H_133_N_25_O_30_Cl_2_ | 2222.9100 | - |  | 1111.9586 | 1111.9769 | 16.4574 | 741.6415 | 741.6541 | 16.9893 |
| End_CA | C_107_H_138_N_26_O_31_Cl_2_ | 2353.9471 | 2353.9444 | -1.1470 | 1177.4772 | 1177.4807 | 2.9725 | 785.3206 | 785.3236 | 3.8201 |
| End_LA | C_107_H_140_Cl_2_N_26_O_32_ | 2371.9577 |  |  | 1186.4825 | 1186.4771 | -4.5513 | 791.3241 | 791.3216 | -3.1593 |
|  | Formula | **[M+1NH_4_]^1+^** | | | **[M+2]** | | | **[M+3(NH_4_)]^3+^** | | |
|  |  | Calc. | Obs. | Err. | Calc. | Obs. | Err. | Calc. | Obs. | Err. |
| End_L9 | C_63_H_88_N_12_O_20_ | 1350.6576 | 1350.6314 | -19.3980 | 666.3113 | 666.3305 | 28.8154 | 462.2418 | 462.2430 | 2.5960 |
|  |  | **[M+1Na]^1+^** | | |  | | | | | |
|  |  | Calc. | Obs. | Err. |  |  |  |  |  |  |
|  |  | 1355.6130 | 1355.6203 | 5.3850 |  |  |  |  |  |  |
|  |  | **[M+1K]^1+^** | | |  |  |  |  |  |  |
|  |  | Calc. | Obs. | Err. |  |  |  |  |  |  |
|  |  | 1371.5869 | 1371.6253 | 27.9968 |  |  |  |  |  |  |

**Table S10** Theoretical and observed *m/z* values of parent and fragment ions of End_C9.

| End_C9 | Formula | [M+3H]^3+^ | | | [M+2H]^2+^ | | | [M+1H]^1+^ | | |
| --- | --- | --- | --- | --- | --- | --- | --- | --- | --- | --- |
|  |  | Calc. | Obs. | Err. | Calc. | Obs. | Err. | Calc. | Obs. | Err. |
| parent | C_63_H_86_N_12_O_19_ | 439.2117 | - |  | 658.3139 | 658.3155 | 2.4305 | 1315.6205 | 1315.6227 | 1.6722 |
| p-H_2_O | C_63_H_84_N_12_O_18_ | 433.2082 | - |  | 649.3086 | 649.3106 | 3.0802 | 1297.6099 | - |  |
| z1 | C_47_H_59_N_10_O_15_ | 335.4793 | - |  | 502.7153 | 502.7284 | 26.0585 | 1004.4234 | 1004.4368 | 13.3410 |
| y1 | C_47_H_63_N_11_O_15_ | 341.4908 | - |  | 511.7325 | 511.7331 | 1.1725 | 1022.4578 | 1022.4569 | -0.8802 |
| z2 | C_51_H_64_N_11_O_18_ | 373.8216 | - |  | 560.2288 | - |  | 1120.4582 | 1120.4662 | 7.1399 |
| y2 | C_51_H_68_N_12_O_18_ | 379.8331 | - |  | 569.246 | - |  | 1137.4847 | 1137.4845 | -0.1758 |
| b1 | C_12_H_18_O |  |  |  |  |  |  | 179.1430 | 179.1433 | 1.6746 |
| b2 | C_16_H_23_NO_4_ |  |  |  |  |  |  | 294.1700 | 294.1699 | -0.3399 |

**Table S11** Theoretical and observed *m/z* values of parent and fragment ions of End_C11.

| End_C11 | Formula | [M+3H]^3+^ | | | [M+2H]^2+^ | | | [M+1H]^1+^ | | |
| --- | --- | --- | --- | --- | --- | --- | --- | --- | --- | --- |
|  |  | Calc. | Obs. | Err. | Calc. | Obs. | Err. | Calc. | Obs. | Err. |
| parent | C_77_H_103_N_17_O_22_ | 540.2561 | - |  | 809.8805 | 809.8820 | 1.8521 | 1618.7536 |  |  |
| p-H_2_O | C_77_H_101_N_17_O_21_ | 534.2525 | - |  | 800.8752 | 800.8776 | 2.9967 | 1600.7431 | - |  |
| z1 | C_61_H_76_N_15_O_18_ | 436.8596 | - |  | 654.7858 |  |  | 1308.5644 | 1308.5639 | -0.3821 |
| y1 | C_61_H_80_N_16_O_18_ | 442.5352 | - |  | 663.2991 | 663.3018 | 4.0706 | 1325.5909 | 1325.5954 | 3.3947 |
| z2 | C_65_H_82_N_16_O_21_ | 475.2020 | - |  | 712.2993 | 712.3048 | 7.7215 | 1423.5913 | 1423.5945 | 2.2478 |
| y2 | C_65_H_85_N_17_O_21_ | 480.8775 | - |  | 720.8126 |  |  | 1440.6179 | 1440.6176 | -0.2082 |
| b1 | C_12_H_19_O |  |  |  |  |  |  | 179.1430 | 179.1433 | 1.6746 |
| b2 | C_16_H_24_NO_4_ |  |  |  |  |  |  | 294.1700 | 294.1713 | 4.4192 |

**Table S12** Theoretical and observed *m/z* values of parent and fragment ions of End_L11.

| End_L11 | Formula | [M+3H]^3+^ | | | [M+2H]^2+^ | | | [M+1H]^1+^ | | |
| --- | --- | --- | --- | --- | --- | --- | --- | --- | --- | --- |
|  |  | Calc. | Obs. | Err. | Calc. | Obs. | Err. | Calc. | Obs. | Err. |
| y1 | C_8_H_9_NO_3_ |  |  |  |  |  |  | 168.0655 |  |  |
| y2 | C_14_H_19_N_5_O_4_ |  |  |  |  |  |  | 322.1510 | 322.1510 | 0.0000 |
| y3 | C_20_H_30_N_8_O_6_ |  |  |  |  |  |  | 479.2361 |  |  |
| y4 | C_24_H_37_N_9_O_8_ |  |  |  | 290.6455 |  |  | 580.2838 | 580.2844 | 1.0340 |
| y5 | C_32_H_44_N_10_O_10_ |  |  |  | 365.1694 |  |  | 729.3315 | 729.3313 | -0.2742 |
| y6 | C_40_H_51_N_11_O_12_ |  |  |  | 439.6932 |  |  | 878.3791 | 878.3784 | -0.7969 |
| y7 | C_44_H_58_N_12_O_14_ |  |  |  | 490.2170 |  |  | 979.4268 | 979.4278 | 1.0210 |
| y8 | C_49_H_68_N_14_O_15_ |  |  |  | 547.2567 | 547.2567 | 0.0000 | 1093.5061 | 1093.5079 | 1.6461 |
| y9 | C_57_H_75_N_15_O_17_ |  |  |  | 621.7805 |  |  | 1242.5538 |  |  |
| y10 | C_61_H_82_N_16_O_19_ |  |  |  | 672.3044 | 672.3052 | 1.1899 | 1343.6015 | 1343.6017 | 0.1489 |
| y11 | C_65_H_87_N_17_O_22_ |  |  |  | 729.8179 |  |  | 1458.6284 | 1458.6289 | 0.3428 |
| p-H_2_O | C_77_H_103_N_17_O_22_ | 540.2561 |  |  | 809.8805 | 809.8822 | 2.0991 | 1618.7536 |  |  |
| parent | C_77_H_105_N_17_O_23_ | 546.2596 |  |  | 818.8857 | 818.8862 | 0.6106 | 1636.7642 |  |  |
| b1 | C_12_H_18_O |  |  |  |  |  |  | 179.1430 | 179.1431 | 0.5582 |
| b2 | C_16_H_23_NO_4_ |  |  |  |  |  |  | 294.1700 | 294.1696 | -1.3598 |
| b3 | C_20_H_30_N_2_O_6_ |  |  |  |  |  |  | 395.2177 | 395.2191 | 3.5424 |
| b4 | C_28_H_37_N_3_O_8_ |  |  |  |  |  |  | 544.2653 | 544.2657 | 0.7349 |
| b5 | C_33_H_47_N_5_O_9_ |  |  |  |  |  |  | 658.3447 | 658.3424 | -3.4936 |
| b6 | C_37_H_54_N_6_O_11_ |  |  |  | 380.1998 |  |  | 759.3923 | 759.3928 | 0.6584 |
| b7 | C_45_H_61_N_7_O_13_ |  |  |  | 454.7236 |  |  | 908.4400 |  |  |
| b8 | C_53_H_68_N_8_O_15_ |  |  |  | 529.2475 |  |  | 1057.4877 |  |  |
| b9 | C_57_H_75_N_9_O_17_ |  |  |  | 579.7713 |  |  | 1158.5354 |  |  |
| b10 | C_63_H_86_N_12_O_19_ |  |  |  | 658.3139 |  |  | 1315.6205 |  |  |
| b11 | C_69_H_96_N_16_O_20_ |  |  |  | 735.3566 |  |  | 1469.7060 |  |  |

**Table S13** Theoretical and observed *m/z* values of parent and fragment ions of End_C12.

| End_C12 | Formula | [M+3H]^3+^ | | | | | | [M+2H]^2+^ | | | | | | [M+1H]^1+^ | | | |
| --- | --- | --- | --- | --- | --- | --- | --- | --- | --- | --- | --- | --- | --- | --- | --- | --- | --- |
|  |  | Calc. | | Obs. | | Err. | | Calc. | | Obs. | | Err. | | Calc. | | Obs. | Err. |
| parent | C_80_H_108_N_18_O_24_ | 569.2667 | | 569.2680 | | 2.2836 | | 853.3965 | | 853.3978 | | 1.5233 | | 1705.7857 | | 1705.7885 | 1.6415 |
| p-H_2_O | C_80_H_106_N_18_O_23_ | 563.2632 | |  | |  | | 844.3912 | | 844.3904 | | -0.9474 | | 1687.7751 | |  |  |
| z1 | C_64_H_81_N_16_O_20_ | 465.5344 | |  | |  | | 697.7979 | | 697.8082 | | 14.7607 | | 1394.5886 | |  |  |
| y1 | C_64_H_85_N_17_O_20_ | 471.5458 | |  | |  | | 706.8151 | | 706.8164 | | 1.8392 | | 1412.6230 | | 1412.6209 | -1.4866 |
| z2 | C_68_H_86_N_17_O_23_ | 503.8767 | |  | |  | | 755.3114 | |  | |  | | 1509.6155 | |  |  |
| y2 | C_68_H_90_N_18_O_23_ | 509.8882 | |  | |  | | 764.3286 | |  | |  | | 1527.6499 | | 1527.6487 | -0.7855 |
| b1 | C_12_H_18_O |  | |  | |  | |  | |  | |  | | 179.1430 | | 179.1430 | 0.0000 |
| b2 | C_16_H_23_NO_4_ | |  | |  | |  | |  | |  | |  | | 294.1700 | 294.1694 | -2.0396 |

**Table S14** Theoretical and observed *m/z* values of parent and fragment ions of End_L12.

| End_L12 | Formula | [M+3H]^3+^ | | | [M+2H]^2+^ | | | [M+1H]^1+^ | | |
| --- | --- | --- | --- | --- | --- | --- | --- | --- | --- | --- |
|  |  | Calc. | Obs. | Err. | Calc. | Obs. | Err. | Calc. | Obs. | Err. |
| y1 | C_3_H_7_NO_3_ |  |  |  |  |  |  | 106.0499 |  |  |
| y2 | C_11_H_14_N_2_O_5_ |  |  |  |  |  |  | 255.0975 |  |  |
| y3 | C_17_H_24_N_6_O_6_ |  |  |  |  |  |  | 409.183 | 409.1832 | 0.4888 |
| y4 | C_23_H_35_N_9_O_8_ |  |  |  | 283.6377 |  |  | 566.2681 |  |  |
| y5 | C_27_H_42_N_10_O_10_ | 223.1101 |  |  | 334.1615 |  |  | 667.3158 | 667.3163 | 0.7493 |
| y6 | C_35_H_49_N_11_O_12_ | 272.7927 |  |  | 408.6854 |  |  | 816.3635 | 816.3626 | -1.1025 |
| y7 | C_43_H_56_N_12_O_14_ | 322.4752 |  |  | 483.2092 |  |  | 965.4112 | 965.4115 | 0.3107 |
| y8 | C_47_H_63_N_13_O_16_ | 356.1578 |  |  | 533.7331 |  |  | 1066.4588 | 1066.4590 | 0.1875 |
| y9 | C_52_H_73_N_15_O_17_ | 394.1842 |  |  | 590.7727 | 590.775 | 3.8932 | 1180.5382 | 1180.5372 | -0.8471 |
| y10 | C_60_H_80_N_16_O_19_ | 443.8668 |  |  | 665.2966 |  |  | 1329.5858 |  |  |
| y11 | C_64_H_87_N_17_O_21_ | 477.5494 |  |  | 715.8204 | 715.822 | 2.2352 | 1430.6335 |  |  |
| y12 | C_68_H_92_N_18_O_24_ | 515.8917 |  |  | 773.3339 |  |  | 1545.6605 | 1545.6586 | -1.2292 |
| p-H_2_O | C_80_H_108_N_18_O_24_ | 569.2667 |  |  | 853.3965 | 853.3975 | 1.1718 | 1705.7857 |  |  |
| parent | C_80_H_110_N_18_O_25_ | 575.2703 |  |  | 862.4018 | 862.4038 | 2.3191 | 1723.7962 |  |  |
| b1 | C_12_H_18_O |  |  |  |  |  |  | 179.1430 | 179.1429 | -0.5582 |
| b2 | C_16_H_23_NO_4_ |  |  |  |  |  |  | 294.1700 | 294.1701 | 0.3399 |
| b3 | C_20_H_30_N_2_O_6_ |  |  |  |  |  |  | 395.2177 | 395.2202 | 6.3256 |
| b4 | C_28_H_37_N_3_O_8_ |  |  |  |  |  |  | 544.2653 | 544.2661 | 1.4699 |
| b5 | C_33_H_47_N_5_O_9_ |  |  |  |  |  |  | 658.3447 | 658.3448 | 0.1519 |
| b6 | C_37_H_54_N_6_O_11_ |  |  |  | 380.1998 |  |  | 759.3923 | 759.3927 | 0.5267 |
| b7 | C_45_H_61_N_7_O_13_ |  |  |  | 454.7236 |  |  | 908.4400 |  |  |
| b8 | C_53_H_68_N_8_O_15_ |  |  |  | 529.2475 |  |  | 1057.4877 |  |  |
| b9 | C_57_H_75_N_9_O_17_ |  |  |  | 579.7713 |  |  | 1158.5354 |  |  |
| b10 | C_63_H_86_N_12_O_19_ | 439.2117 |  |  | 658.3139 |  |  | 1315.6205 |  |  |
| b11 | C_69_H_96_N_16_O_20_ | 490.5735 |  |  | 735.3566 | 735.3554 | -1.6319 | 1469.7060 |  |  |
| b12 | C_77_H_103_N_17_O_22_ | 540.2561 |  |  | 809.8805 |  |  | 1618.7536 |  |  |

**Table S15** Theoretical and observed *m/z* values of parent and fragment ions of End_C13.

| End_C13 | Formula | [M+3H]^3+^ | | | | | | [M+2H]^2+^ | | | | | | [M+1H]^1+^ | | | |
| --- | --- | --- | --- | --- | --- | --- | --- | --- | --- | --- | --- | --- | --- | --- | --- | --- | --- |
|  |  | Calc. | | Obs. | | Err. | | Calc. | | Obs. | | Err. | | Calc. | | Obs. | Err. |
| parent | C_88_H_113_Cl_2_N_19_O_26_ | 641.5900 | |  | |  | | 961.8813 | | 961.8835 | | 2.2872 | | 1922.7554 | |  |  |
| p-H_2_O | C_88_H_111_Cl_2_N_19_O_25_ | 635.5865 | |  | |  | | 952.8761 | | 952.8777 | | 1.6791 | | 1904.7448 | |  |  |
| z1 | C_72_H_86_Cl_2_N_17_O_22_ | 537.8576 | |  | |  | | 806.2828 | | 806.2934 | | 13.1468 | | 1611.5583 | |  |  |
| y1 | C_72_H_90_Cl_2_N_18_O_22_ | 543.8691 | |  | |  | | 815.3000 | | 815.3010 | | 1.2265 | | 1629.5927 | |  |  |
| z2 | C_76_H_91_Cl_2_N_18_O_25_ | 576.1999 | |  | |  | | 863.7963 | |  | |  | | 1726.5853 | |  |  |
| y2 | C_76_H_95_Cl_2_N_19_O_25_ | 582.2114 | |  | |  | | 872.8135 | |  | |  | | 1744.6196 | | 1744.6228 | 1.8342 |
| b1 | C_12_H_18_O |  | |  | |  | |  | |  | |  | | 179.1430 | | 179.1430 | 0.0000 |
| b2 | C_16_H_23_NO_4_ | |  | |  | |  | |  | |  | |  | | 294.1700 | 294.1708 | 2.7195 |

**Table S16** Theoretical and observed *m/z* values of parent and fragment ions of End_L13.

| End_L13 | Formula | [M+3H]^3+^ | | | [M+2H]^2+^ | | | [M+1H]^1+^ | | |
| --- | --- | --- | --- | --- | --- | --- | --- | --- | --- | --- |
|  |  | Calc. | Obs. | Err. | Calc. | Obs. | Err. | Calc. | Obs. | Err. |
| y1 | C_8_H_7_Cl_2_NO_3_ |  |  |  |  |  |  | 235.9876 |  |  |
| y2 | C_11_H_12_Cl_2_N_2_O_5_ |  |  |  |  |  |  | 323.0196 |  |  |
| y3 | C_19_H_19_Cl_2_N_3_O_7_ |  |  |  |  |  |  | 472.0673 |  |  |
| y4 | C_25_H_29_Cl_2_N_7_O_8_ |  |  |  |  |  |  | 626.1527 | 626.1505 | -3.5135 |
| y5 | C_31_H_40_Cl_2_N_10_O_10_ |  |  |  |  |  |  | 783.2379 |  |  |
| y6 | C_35_H_47_Cl_2_N_11_O_12_ |  |  |  |  |  |  | 884.2855 |  |  |
| y7 | C_43_H_54_Cl_2_N_12_O_14_ | 345.1159 |  |  | 517.1703 |  |  | 1033.3332 | 1033.3350 | 1.7419 |
| y8 | C_51_H_61_Cl_2_N_13_O_16_ | 394.7985 |  |  | 591.6941 |  |  | 1182.3809 | 1182.3800 | -0.7612 |
| y9 | C_55_H_68_Cl_2_N_14_O_18_ | 428.4810 |  |  | 642.2179 |  |  | 1283.4286 | 1283.4270 | -1.2467 |
| y10 | C_60_H_78_Cl_2_N_16_O_19_ | 466.5075 |  |  | 699.2576 |  |  | 1397.5079 |  |  |
| y11 | C_68_H_85_Cl_2_N_17_O_21_ | 516.1900 |  |  | 773.7814 |  |  | 1546.5556 |  |  |
| y12 | C_72_H_92_Cl_2_N_18_O_23_ | 549.8726 |  |  | 824.3053 | 824.3065 | 1.4558 | 1647.6033 |  |  |
| y13 | C_76_H_97_Cl_2_N_19_O_26_ | 588.2149 |  |  | 881.8187 |  |  | 1762.6302 |  |  |
| p-H_2_O | C_88_H_113_Cl_2_N_19_O_26_ | 641.5900 |  |  | 961.8813 | 961.8814 | 0.1040 | 1922.7554 |  |  |
| parent | C_88_H_115_Cl_2_N_19_O_27_ | 647.5935 |  |  | 970.8866 | 970.8897 | 3.1930 | 1940.7660 |  |  |
| b1 | C_12_H_18_O |  |  |  |  |  |  | 179.1430 | 179.1429 | -0.5582 |
| b2 | C_16_H_23_NO_4_ |  |  |  |  |  |  | 294.1700 | 294.1704 | 1.3598 |
| b3 | C_20_H_30_N_2_O_6_ |  |  |  |  |  |  | 395.2177 | 395.2163 | -3.5424 |
| b4 | C_28_H_37_N_3_O_8_ |  |  |  |  |  |  | 544.2653 | 544.2651 | -0.3675 |
| b5 | C_33_H_47_N_5_O_9_ |  |  |  |  |  |  | 658.3447 | 658.3408 | -5.9239 |
| b6 | C_37_H_54_N_6_O_11_ |  |  |  | 380.1998 |  |  | 759.3923 | 759.3955 | 4.2139 |
| b7 | C_45_H_61_N_7_O_13_ |  |  |  | 454.7236 |  |  | 908.4400 |  |  |
| b8 | C_53_H_68_N_8_O_15_ |  |  |  | 529.2475 |  |  | 1057.4877 |  |  |
| b9 | C_57_H_75_N_9_O_17_ |  |  |  | 579.7713 |  |  | 1158.5354 |  |  |
| b10 | C_63_H_86_N_12_O_19_ |  |  |  | 658.3139 |  |  | 1315.6205 |  |  |
| b11 | C_69_H_96_N_16_O_20_ | 490.5735 |  |  | 735.3566 |  |  | 1469.7060 |  |  |
| b12 | C_77_H_103_N_17_O_22_ | 540.2561 |  |  | 809.8805 |  |  | 1618.7536 |  |  |
| b13 | C_80_H_108_N_18_O_24_ | 569.2667 |  |  | 853.3965 |  |  | 1705.7857 |  |  |

**Table S17** Theoretical and observed *m/z* values of parent and fragment ions of End_C14.

| End_C14 | Formula | [M+3H]^3+^ | | | | | | [M+2H]^2+^ | | | | | | [M+1H]^1+^ | | | |
| --- | --- | --- | --- | --- | --- | --- | --- | --- | --- | --- | --- | --- | --- | --- | --- | --- | --- |
|  |  | Calc. | | Obs. | | Err. | | Calc. | | Obs. | | Err. | | Calc. | | Obs. | Err. |
| parent | C_90_H_116_N_20_O_27_Cl_2_ | 660.5971 | |  | |  | | 990.3921 | | 990.3947 | | 2.6252 | | 1979.7769 | |  |  |
| p-H_2_O | C_90_H_114_N_20_O_26_Cl_2_ | 654.3656 | |  | |  | | 981.3868 | |  | |  | | 1961.7663 | |  |  |
| z1 | C_74_H_89_Cl_2_N_18_O_23_• | 556.8648 | |  | |  | | 834.7935 | | 834.8068 | | 15.9321 | | 1668.5798 | |  |  |
| y1 | C_74_H_93_Cl_2_N_19_O_23_ | 562.8762 | |  | |  | | 843.8107 | | 843.8132 | | 2.9627 | | 1686.6142 | |  |  |
| z2 | C_78_H_94_Cl_2_N_19_O_26_ | 595.2071 | |  | |  | | 892.3070 | |  | |  | | 1783.6067 | |  |  |
| y2 | C_78_H_98_Cl_2_N_20_O_26_ | 601.2185 | |  | |  | | 901.3242 | |  | |  | | 1801.6411 | | 1801.6426 | 0.8326 |
| b1 | C_12_H_18_O |  | |  | |  | |  | |  | |  | | 179.1430 | | 179.1434 | 2.2329 |
| b2 | C_16_H_23_NO_4_ | |  | |  | |  | |  | |  | |  | | 294.1700 | 294.1702 | 0.6799 |

**Table S18** Theoretical and observed *m/z* values of parent and fragment ions of End_L14.

| End_L14 | Formula | [M+3H]^3+^ | | | [M+2H]^2+^ | | | [M+1H]^1+^ | | |
| --- | --- | --- | --- | --- | --- | --- | --- | --- | --- | --- |
|  |  | Calc. | Obs. | Err. | Calc. | Obs. | Err. | Calc. | Obs. | Err. |
| y1 | C_2_H_5_NO_2_ |  |  |  |  |  |  | 76.0393 |  |  |
| y2 | C_10_H_10_Cl_2_N_2_O_4_ |  |  |  |  |  |  | 147.0082 |  |  |
| y3 | C_13_H_15_Cl_2_N_3_O_6_ |  |  |  |  |  |  | 190.5242 |  |  |
| y4 | C_21_H_22_Cl_2_N_4_O_8_ |  |  |  | 265.0480 |  |  | 529.0887 |  |  |
| y5 | C_27_H_32_Cl_2_N_8_O_9_ |  |  |  | 342.0907 |  |  | 683.1742 |  |  |
| y6 | C_33_H_43_Cl_2_N_11_O_11_ |  |  |  | 420.6333 |  |  | 840.2593 |  |  |
| y7 | C_37_H_50_Cl_2_N_12_O_13_ |  |  |  | 471.1571 |  |  | 941.3070 |  |  |
| y8 | C_45_H_57_Cl_2_N_13_O_15_ | 364.1231 |  |  | 545.6810 |  |  | 1090.3547 | 1090.3464 | -7.6122 |
| y9 | C_53_H_64_Cl_2_N_14_O_17_ | 413.8056 |  |  | 620.2048 |  |  | 1239.4024 | 1239.391 | -9.1980 |
| y10 | C_57_H_71_Cl_2_N_15_O_19_ | 447.4882 |  |  | 670.7287 |  |  | 1340.4500 | 1340.4505 | 0.3730 |
| y11 | C_62_H_81_Cl_2_N_17_O_20_ | 485.5146 |  |  | 727.7683 |  |  | 1454.5294 | 1454.5175 | -8.1813 |
| y12 | C_70_H_88_Cl_2_N_18_O_22_ | 535.1972 |  |  | 802.2922 |  |  | 1603.5770 |  |  |
| y13 | C_74_H_95_Cl_2_N_19_O_24_ | 568.8798 |  |  | 852.8160 | 852.8204 | 5.1594 | 1704.6247 |  |  |
| y14 | C_78_H_100_Cl_2_N_20_O_27_ | 607.2221 |  |  | 910.3295 |  |  | 1819.6517 |  |  |
| p-H_2_O | C_90_H_116_Cl_2_N_20_O_27_ | 660.5971 |  |  | 990.3921 |  |  | 1979.7769 |  |  |
| parent | C_90_H_118_Cl_2_N_20_O_28_ | 666.6007 |  |  | 999.3974 | 999.3986 | 1.2007 | 1997.7874 |  |  |
| b1 | C_12_H_18_O |  |  |  |  |  |  | 179.1430 |  |  |
| b2 | C_16_H_23_NO_4_ |  |  |  |  |  |  | 294.1700 | 294.1697 | -1.0198 |
| b3 | C_20_H_30_N_2_O_6_ |  |  |  |  |  |  | 395.2177 | 395.2155 | -5.5666 |
| b4 | C_28_H_37_N_3_O_8_ |  |  |  |  |  |  | 544.2653 | 544.2588 | -11.9427 |
| b5 | C_33_H_47_N_5_O_9_ |  |  |  |  |  |  | 658.3447 | 658.3396 | -7.7467 |
| b6 | C_37_H_54_N_6_O_11_ |  |  |  | 380.1998 |  |  | 759.3923 | 759.3921 | -0.2634 |
| b7 | C_45_H_61_N_7_O_13_ |  |  |  | 454.7236 |  |  | 908.4400 |  |  |
| b8 | C_53_H_68_N_8_O_15_ |  |  |  | 529.2475 |  |  | 1057.4877 |  |  |
| b9 | C_57_H_75_N_9_O_17_ |  |  |  | 579.7713 |  |  | 1158.5354 |  |  |
| b10 | C_63_H_86_N_12_O_19_ |  |  |  | 658.3139 |  |  | 1315.6205 |  |  |
| b11 | C_69_H_96_N_16_O_20_ | 490.5735 |  |  | 735.3566 |  |  | 1469.7060 |  |  |
| b12 | C_77_H_103_N_17_O_22_ | 540.2561 |  |  | 809.8805 |  |  | 1618.7536 |  |  |
| b13 | C_80_H_108_N_18_O_24_ | 569.2667 |  |  | 853.3965 |  |  | 1705.7857 |  |  |
| b14 | C_88_H_113_Cl_2_N_19_O_26_• | 641.5900 |  |  | 961.8813 |  |  | 1922.7554 |  |  |

**Table S19** Theoretical and observed *m/z* values of parent and fragment ions of End_C15.

| End_C15 | Formula | [M+3H]^3+^ | | | | | | [M+2H]^2+^ | | | | | | [M+1H]^1+^ | | | |
| --- | --- | --- | --- | --- | --- | --- | --- | --- | --- | --- | --- | --- | --- | --- | --- | --- | --- |
|  |  | Calc. | | Obs. | | Err. | | Calc. | | Obs. | | Err. | | Calc. | | Obs. | Err. |
| parent | C_96_H_126_N_24_O_28_Cl_2_ | 711.9590 | | 711.9605 | | 2.1069 | | 1067.4348 | | 1067.4352 | | 0.3747 | | 2133.8623 | |  |  |
| p-H_2_O | C_96_H_124_N_24_O_27_Cl_2_ | 705.9554 | |  | |  | | 1058.4295 | |  | |  | | 2115.8518 | |  |  |
| z1 | C_80_H_99_Cl_2_N_22_O_24_ | 608.2266 | |  | |  | | 911.8363 | |  | |  | | 1822.6652 | |  |  |
| y1 | C_80_H_103_Cl_2_N_23_O_24_ | 614.2381 | | 614.2327 | | -8.7914 | | 920.8534 | | 920.8661 | | 13.7916 | | 1840.6996 | |  |  |
| z2 | C_84_H_104_Cl_2_N_23_O_27_ | 646.5689 | |  | |  | | 969.3497 | |  | |  | | 1937.6922 | |  |  |
| y2 | C_84_H_108_Cl_2_N_24_O_27_ | 652.5804 | |  | |  | | 978.3669 | | 978.3650 | | -1.9420 | | 1955.7266 | |  |  |
| b1 | C_12_H_18_O |  | |  | |  | |  | |  | |  | | 179.1430 | | 179.1427 | -1.6746 |
| b2 | C_16_H_23_NO_4_ | |  | |  | |  | |  | |  | |  | | 294.1700 |  |  |

**Table S20** Theoretical and observed *m/z* values of parent and fragment ions of End_L15.

| End_L15 | Formula | [M+3H]^3+^ |  |  | [M+2H]^2+^ | | | [M+1H]^1+^ | | |
| --- | --- | --- | --- | --- | --- | --- | --- | --- | --- | --- |
|  |  | Calc. | Obs. | Err. | Calc. | Obs. | Err. | Calc. | Obs. | Err. |
| y1 | C_6_H_12_N_4_O_2_ |  |  |  |  |  |  | 173.1033 | 173.1032 | -0.5777 |
| y2 | C_8_H_15_N_5_O_3_ |  |  |  |  |  |  | 230.1248 | 230.1253 | 2.1727 |
| y3 | C_16_H_20_Cl_2_N_6_O_5_ |  |  |  |  |  |  | 447.0945 |  |  |
| y4 | C_19_H_25_Cl_2_N_7_O_7_ |  |  |  | 267.5669 |  |  | 534.1265 |  |  |
| y5 | C_27_H_32_Cl_2_N_8_O_9_ |  |  |  | 342.0907 |  |  | 683.1742 | 683.1731 | -1.6101 |
| y6 | C_33_H_42_Cl_2_N_12_O_10_ | 279.7581 |  |  | 419.1335 |  |  | 837.2597 | 837.2535 | -7.4051 |
| y7 | C_39_H_53_Cl_2_N_15_O_12_ | 332.1198 |  |  | 497.6760 |  |  | 994.3448 |  |  |
| y8 | C_43_H_60_Cl_2_N_16_O_14_ | 365.8023 |  |  | 548.1999 |  |  | 1095.3925 |  |  |
| y9 | C_51_H_67_Cl_2_N_17_O_16_ | 415.4849 |  |  | 622.7237 | 622.7261 | 3.8540 | 1244.4402 |  |  |
| y10 | C_59_H_74_Cl_2_N_18_O_18_ | 465.1675 |  |  | 697.2476 |  |  | 1393.4878 |  |  |
| y11 | C_63_H_81_Cl_2_N_19_O_20_ | 498.8500 |  |  | 747.7714 | 747.7761 | 6.2853 | 1494.5355 |  |  |
| y12 | C_68_H_91_Cl_2_N_21_O_21_ | 536.8765 |  |  | 804.8110 |  |  | 1608.6148 |  |  |
| y13 | C_76_H_98_Cl_2_N_22_O_23_ | 586.5590 |  |  | 879.3349 |  |  | 1757.6625 |  |  |
| y14 | C_80_H_105_Cl_2_N_23_O_25_ | 620.2416 |  |  | 929.8587 | 929.8529 | -6.2375 | 1858.7102 |  |  |
| y15 | C_84_H_110_Cl_2_N_24_O_28_ | 658.5839 |  |  | 987.3722 | 987.3770 | 4.8614 | 1973.7371 |  |  |
| p-H_2_O | C_96_ H_126_ N_24_ O_28_ Cl_2_ | 711.9590 |  |  | 1067.4348 |  |  | 2133.8623 |  |  |
| parent | C_96_ H_128_ N_24_ O_29_ Cl_2_ | 717.9625 | 717.9650 | 3.4821 | 1076.4401 |  |  | 2151.8729 |  |  |
| b1 | C_12_H_18_O |  |  |  |  |  |  | 179.1430 | 179.1427 | -1.6746 |
| b2 | C_16_H_23_NO_4_ |  |  |  |  |  |  | 294.1700 | 294.1688 | -4.0793 |
| b3 | C_20_H_30_N_2_O_6_ |  |  |  |  |  |  | 395.2177 |  |  |
| b4 | C_28_H_37_N_3_O_8_ |  |  |  |  |  |  | 544.2653 |  |  |
| b5 | C_33_H_47_N_5_O_9_ |  |  |  |  |  |  | 658.3447 |  |  |
| b6 | C_37_H_54_N_6_O_11_ |  |  |  | 380.1998 |  |  | 759.3923 |  |  |
| b7 | C_45_H_61_N_7_O_13_ |  |  |  | 454.7236 |  |  | 908.4400 |  |  |
| b8 | C_53_H_68_N_8_O_15_ |  |  |  | 529.2475 |  |  | 1057.4877 |  |  |
| b9 | C_57_H_75_N_9_O_17_ |  |  |  | 579.7713 |  |  | 1158.5354 |  |  |
| b10 | C_63_H_86_N_12_O_19_ |  |  |  | 658.3139 |  |  | 1315.6205 |  |  |
| b11 | C_69_H_96_N_16_O_20_ | 490.5735 |  |  | 735.3566 |  |  | 1469.7060 |  |  |
| b12 | C_77_H_103_N_17_O_22_ | 540.2561 |  |  | 809.8805 |  |  | 1618.7536 |  |  |
| b13 | C_80_H_108_N_18_O_24_ | 569.2667 |  |  | 853.3965 |  |  | 1705.7857 |  |  |
| b14 | C_88_H_113_Cl_2_N_19_O_26_• | 641.5900 |  |  | 961.8813 |  |  | 1922.7554 |  |  |
| b15 | C90H116Cl2N20O27 | 660.5971 |  |  | 990.3921 |  |  | 1979.7769 |  |  |

**Table S21** Theoretical and observed *m/z* values of parent and fragment ions of End_C16.

| End_C16 | Formula | [M+3H]^3+^ | | | | | | [M+2H]^2+^ | | | | | | [M+1H]^1+^ | | | |
| --- | --- | --- | --- | --- | --- | --- | --- | --- | --- | --- | --- | --- | --- | --- | --- | --- | --- |
|  |  | Calc. | | Obs. | | Err. | | Calc. | | Obs. | | Err. | | Calc. | | Obs. | Err. |
| parent | C_99_H_131_N_25_O_29_Cl_2_ | 735.6380 | | 735.6389 | | 1.2234 | | 1102.9534 | |  | |  | | 2204.8994 | |  |  |
| p-H_2_O | C_99_H_129_N_25_O_28_Cl_2_ | 729.6345 | |  | |  | | 1093.9481 | |  | |  | | 2186.8889 | |  |  |
| z1 | C_83_H_104_Cl_2_N_23_O_25_ | 631.9056 | |  | |  | | 947.3548 | |  | |  | | 1893.7024 | |  |  |
| y1 | C_83_H_108_Cl_2_N_24_O_25_ | 637.9171 | | 637.9177 | | 0.9406 | | 956.3720 | | 956.3729 | | 0.9411 | | 1911.7367 | |  |  |
| z2 | C_87_H_109_Cl_2_N_24_O_28_ | 670.2479 | |  | |  | | 1004.8683 | | 1004.8815 | | 13.1360 | | 2008.7293 | |  |  |
| y2 | C_87_H_113_Cl_2_N_25_O_28_ | 676.2594 | |  | |  | | 1013.8855 | | 1013.8864 | | 0.8877 | | 2026.7637 | |  |  |
| b1 | C_12_H_19_O |  | |  | |  | |  | |  | |  | | 179.1430 | | 179.1431 | 0.5582 |
| b2 | C_16_H_24_NO_4_ | |  | |  | |  | |  | |  | |  | | 294.1700 | 294.1700 | 0.0000 |

**Table S22** Theoretical and observed *m/z* values of parent and fragment ions of End_CA.

| End_CA | Formula | [M+3H]^3+^ | | | | | | [M+2H]^2+^ | | | | | | [M+1H]^1+^ | | | |
| --- | --- | --- | --- | --- | --- | --- | --- | --- | --- | --- | --- | --- | --- | --- | --- | --- | --- |
|  |  | Calc. | | Obs. | | Err. | | Calc. | | Obs. | | Err. | | Calc. | | Obs. | Err. |
| parent | C_107_H_138_N_26_O_31_C_l2_ | 785.3206 | | 785.3177 | | -3.6928 | | 1177.4772 | | 1177.4744 | | -2.3780 | | 2353.9471 | | 2353.9497 | 1.1045 |
| p-H_2_O | C_107_H1_36_C_l2_N_26_O_30_ | 779.3170 | |  | |  | | 1168.4719 | |  | |  | | 2335.9365 | |  |  |
| z1 | C_91_H_111_C_l2_N_24_O_27_ | 681.5882 | |  | |  | | 1021.8787 | |  | |  | | 2042.75 | |  |  |
| y1 | C_91_H_115_C_l2_N_25_O_27_ | 687.5997 | | 687.6019 | | 3.1995 | | 1030.8958 | | 1030.8986 | | 2.7161 | | 2060.7844 | |  |  |
| z2 | C_95_H_116_C_l2_N_25_O_30_ | 719.9305 | |  | |  | | 1079.3921 | |  | |  | | 2087.7715 | |  |  |
| y2 | C_95_H_120_C_l2_N_26_O_30_ | 725.942 | |  | |  | | 1088.4093 | | 1088.4115 | | 2.0212 | | 2157.777 | |  |  |
| b1 | C_12_H_19_O |  | |  | |  | |  | |  | |  | | 179.1430 | | 179.1434 | 2.2329 |
| b2 | C_16_H_24_NO_4_ | |  | |  | |  | |  | |  | |  | | 294.1700 | 294.1701 | 0.3399 |

**Table S23** Theoretical and observed *m/z* values of parent and fragment ions of End_LA.

| End_LA | Formula | [M+3H]^3+^ | | | [M+2H]^2+^ | | | [M+1H]^1+^ | | |
| --- | --- | --- | --- | --- | --- | --- | --- | --- | --- | --- |
|  |  | Calc. | Obs. | Err. | Calc. | Obs. | Err. | Calc. | Obs. | Err. |
| y1 | C_8_H_9_NO_3_ |  |  |  |  |  |  | 168.0655 |  |  |
| y2 | C_11_H_14_N_2_O_4_ |  |  |  |  |  |  | 239.1026 |  |  |
| y3 | C_17_H_24_N_6_O_5_ |  |  |  | 197.0977 |  |  | 393.1881 |  |  |
| y4 | C_19_H_27_N_7_O_6_ |  |  |  | 225.6084 |  |  | 450.2096 |  |  |
| y5 | C_27_H_32_Cl_2_N_8_O_8_ | 223.0646 |  |  | 334.0933 |  |  | 667.1793 |  |  |
| y6 | C_30_H_37_Cl_2_N_9_O_10_ | 252.0753 |  |  | 377.6093 |  |  | 754.2113 |  |  |
| y7 | C_38_H_44_Cl_2_N_10_O_12_ | 301.7579 |  |  | 452.1331 |  |  | 903.2590 |  |  |
| y8 | C_44_H_54_Cl_2_N_14_O_13_ | 353.1197 |  |  | 529.1759 |  |  | 1057.3445 |  |  |
| y9 | C_50_H_65_Cl_2_N_17_O_15_ | 405.4814 |  |  | 607.7184 |  |  | 1214.4296 |  |  |
| y10 | C_54_H_72_Cl_2_N_18_O_17_ | 439.1639 |  |  | 658.2423 |  |  | 1315.4773 |  |  |
| y11 | C_62_H_79_Cl_2_N_19_O_19_ | 488.8465 |  |  | 732.7661 | 732.7674 | 1.7741 | 1464.5249 |  |  |
| y12 | C_70_H_86_Cl_2_N_20_O_21_ | 538.5291 |  |  | 807.2899 | 807.2914 | 1.8581 | 1613.5726 |  |  |
| y13 | C_74_H_93_Cl_2_N_21_O_23_ | 572.2116 |  |  | 857.8138 | 857.8152 | 1.6321 | 1714.6203 |  |  |
| y14 | C_79_H_103_Cl_2_N_23_O_24_ | 610.2381 |  |  | 914.8534 | 914.8546 | 1.3117 | 1828.6996 |  |  |
| y15 | C_87_H_110_Cl_2_N_24_O_26_ | 659.9206 |  |  | 989.3773 |  |  | 1977.7473 |  |  |
| y16 | C_91_H_117_Cl_2_N_25_O_28_ | 693.6032 | 693.6068 | 5.1903 | 1039.9011 | 1039.9060 | 4.7120 | 2078.7950 |  |  |
| y17 | C_95_H_122_Cl_2_N_26_O_31_ | 731.9455 |  |  | 1097.4146 | 1097.4178 | 2.9159 | 2193.8219 |  |  |
| p-H_2_O | C_107_H_139_Cl_2_N_26_O_31_ | 785.3206 | 785.3212 | 0.7640 | 1175.4626 |  |  | 2351.9326 |  |  |
| parent | C_107_H_141_Cl_2_N_26_O_32_ | 791.3241 | 791.3277 | 4.5493 | 1186.4825 |  |  | 2371.9577 |  |  |
| b1 | C_12_H_18_O |  |  |  |  |  |  | 179.1430 | 179.1432 | 1.1164 |
| b2 | C_16_H_23_NO_4_ |  |  |  |  |  |  | 294.1700 | 294.1704 | 1.3598 |
| b3 | C_20_H_30_N_2_O_6_ |  |  |  |  |  |  | 395.2177 |  |  |
| b4 | C_28_H_37_N_3_O_8_ |  |  |  |  |  |  | 544.2653 |  |  |
| b5 | C_33_H_47_N_5_O_9_ |  |  |  |  |  |  | 658.3447 |  |  |
| b6 | C_37_H_54_N_6_O_11_ |  |  |  | 380.1998 |  |  | 759.3923 |  |  |
| b7 | C_45_H_61_N_7_O_13_ |  |  |  | 454.7236 |  |  | 908.4400 |  |  |
| b8 | C_53_H_68_N_8_O_15_ |  |  |  | 529.2475 |  |  | 1057.4877 |  |  |
| b9 | C_57_H_75_N_9_O_17_ |  |  |  | 579.7713 |  |  | 1158.5354 |  |  |
| b10 | C_63_H_86_N_12_O_19_ |  |  |  | 658.3139 |  |  | 1315.6205 |  |  |
| b11 | C_69_H_96_N_16_O_20_ | 490.5735 |  |  | 735.3566 |  |  | 1469.7060 |  |  |
| b12 | C_77_H_103_N_17_O_22_ | 540.2561 |  |  | 809.8805 |  |  | 1618.7536 |  |  |
| b13 | C_80_H_108_N_18_O_24_ | 569.2667 |  |  | 853.3965 |  |  | 1705.7857 |  |  |
| b14 | C_88_H_113_Cl_2_N_19_O_26_• | 641.5900 |  |  | 961.8813 |  |  | 1922.7554 |  |  |
| b15 | C_90_H_116_Cl_2_N_20_O_27_ | 660.5971 |  |  | 990.3921 |  |  | 1979.7769 |  |  |
| b16 | C_96_H_126_Cl_2_N_24_O_28_• | 711.9590 |  |  | 1067.4348 |  |  | 2133.8623 |  |  |
| b17 | C_99_H_131_Cl_2_N_25_O_29_ | 735.6380 |  |  | 1102.9534 |  |  | 2204.8994 |  |  |

**Table S24** Details of the TEs included in the phylogenetic analysis (Figure 1E).

| **Protein** | **Accession** | **Organism** | **Type** | **NRPS/PKS** |
| --- | --- | --- | --- | --- |
| TylO | WP_043472398 | *Streptomyces fradiae* | TE II | PKS |
| RifR | AAG52991.1 | *Amycolatopsis mediterranei* S699 | TE II | PKS/NRPS hybrid |
| Fsc_TEII | AAQ82559 | *Streptomyces* sp. FR-008 | TE II | PKS |
| YbtT | AAC69590 | *Yersinia pestis* | TE II | PKS/NRPS hybrid |
| ScoT | CAC37888 | *Streptomyces coelicolor*A3(2) | TE II | PKS |
| PikAV | BCN92182 | *Streptomyces venezuelae* | TE II | PKS |
| MonCII | ANZ52474 | *Streptomyces virginiae* | TE II | PKS |
| MonAX | ANZ52473 | *Streptomyces virginiae* | TE II | PKS |
| MonAIX | ANZ52458 | *Streptomyces virginiae* | TE II | PKS |
| NanE | AAP42868 | *Streptomyces nanchangensis*NS3226 | TE II | PKS |
| TycF | AAC45933 | *Bacillus brevis* | TE II | NRPS |
| SrfAC | AHZ14355 | *Bacillus velezensis* SQR9 | TE I | NRPS |
| SrfAD | AHZ14356 | *Bacillus velezensis* SQR9 | TE II | NRPS |
| RedJ | CAA16185 | *Streptomyces coelicolor*A3(2) | TE II | PKS/NRPS hybrid |
| DEBS_TEII | QUH02367 | *Saccharopolyspora erythraea* | TE II | PKS |
| BacTEII | AZN80917 | *Bacillus licheniformis* | TE II | NRPS |
| KtzF | ABV56586 | *Kutzneria*sp. 744 | TE II | NRPS |
| NikP2 | CAC11138 | *Streptomyces tendae*Tü901 | TE II | NRPS |
| Bph | RSM48735 | *Amycolatopsis balhimycina* DSM 5908 | TE II | NRPS |
| SlgB | CBA11581 | *Streptomyces lydicus*NRRL2433 | TE II | PKS/NRPS hybrid |
| LovG_TEII | GFF13604 | *Aspergillus terreus* | TE II | PKS |
| DEBS_TE | 1KEZ | *Saccharopolyspora erythraea* | TE I | PKS |
| NocB_TE | AAT09805 | *Nocardia uniformis* subsp. *tsuyamanensis* | TE I | NRPS |
| Psy_TE | WP_064118616 | *Pseudomonas fluorescens* | TE I | NRPS |
| TdiA_TE | ABU51602 | *Aspergillus nidulans* | TE I | NRPS |
| GrsB_TE | CAA43838 | *Brevibacillus brevis* | TE I | NRPS |
| Fen_TE | 2CBG | *Bacillus subtilis* | TE I | NRPS |
| entF_TE | CDQ53511 | *Klebsiella pneumoniae* | TE I | NRPS |
| TioS_TE | CAJ34375 | *Micromonospora* sp. ML1 | TE I | NRPS |
| CndF_TE | CAQ43084 | *Chondromyces crocatus* | TE I | NRPS |
| LpiB_TE | AFV70301 | *Pseudomonas fluorescens* | TE I | NRPS |
| CTB1_TE | AAT69682 | *Cercospora nicotianae* | TE I | PKS |
| WS5 | QBA57731 | *Streptomyces* sp. SNM55 | TE II | NRPS |
| WS20 | QBA57738 | *Streptomyces* sp. SNM55 | TE II | NRPS |
| PICS_TE | 1MNQ | *Streptomyces venezuelae* | TE I | PKS |
| TMC_TE | 3LCR | *Streptomyces* sp. CK4412 | TE I | PKS |
| End_25620 | AYL38482 | *Streptomyces fungicidicus* | TE II | NRPS |
| EndC_TE | AYL38486 | *Streptomyces fungicidicus* | TE I | NRPS |
| End_25645 | AYL38487 | *Streptomyces fungicidicus* | TE II | NRPS |
| Ram12_TEII | WZN15521 | *Paractinoplanes ramoplaninifer* | TE II | NRPS |
| Ram17_TE | WZN15526 | *Paractinoplanes ramoplaninifer* | TE I | NRPS |
| Ram18 | WZN15527 | *Paractinoplanes ramoplaninifer* | TE II | NRPS |
| BorB | CAE45660 | *Streptomyces parvulus* | TE II | PKS |

Supplementary Figures

**Figure S1** TPM expression levels of key genes for enduracidin biosynthesis in *Streptomyces fungicidicus* TXX3120 at different time points

**>End_25620**


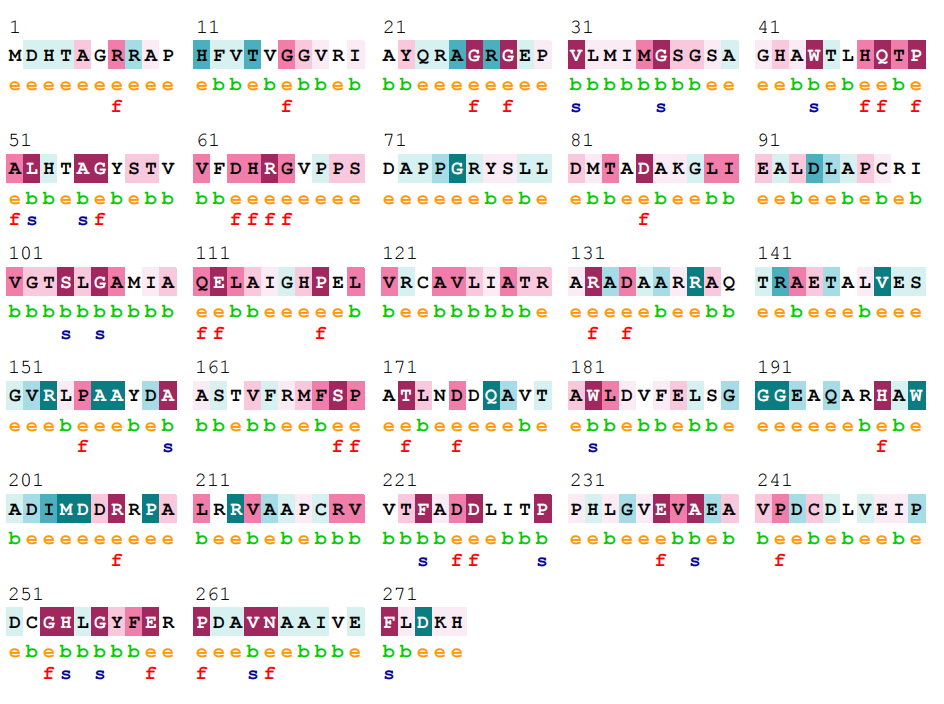


**>EndC_TE**


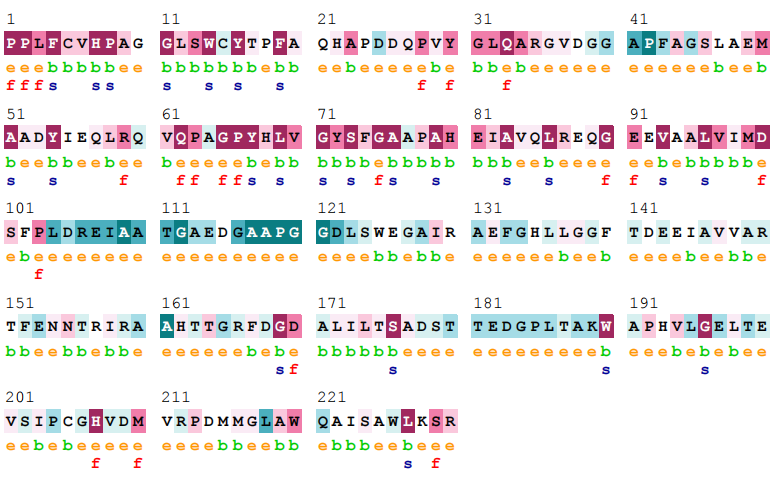


**>End_25645**


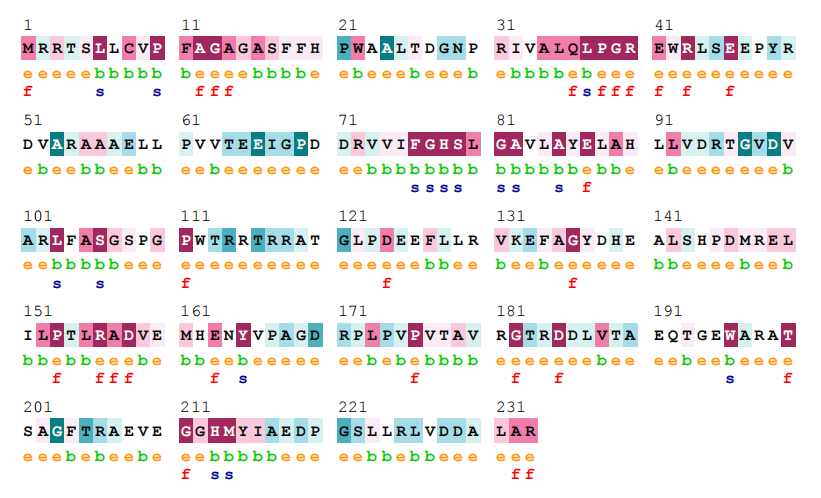


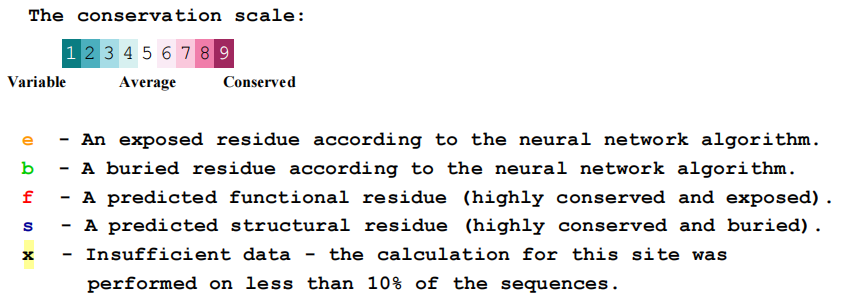


**Figure S2** Conservation analysis of amino acid residues in three TEs.


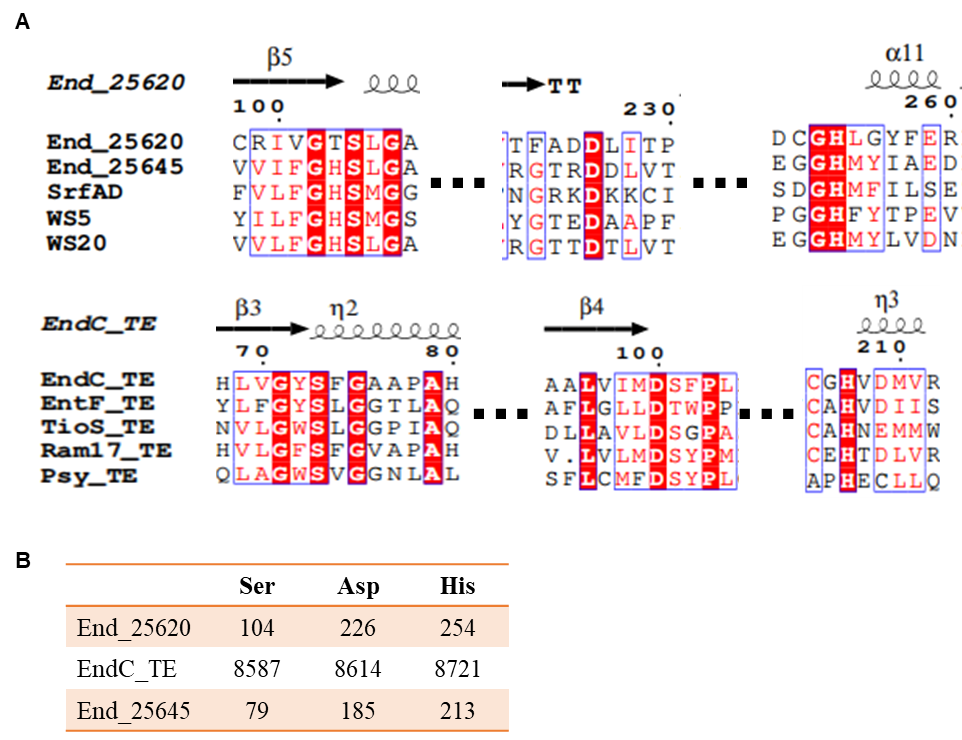


**Figure S3** Conservation of the catalytic triad in three TEs.

(A) Multiple sequence alignment of the catalytic triad. (B) Positions of the catalytic triad residues.


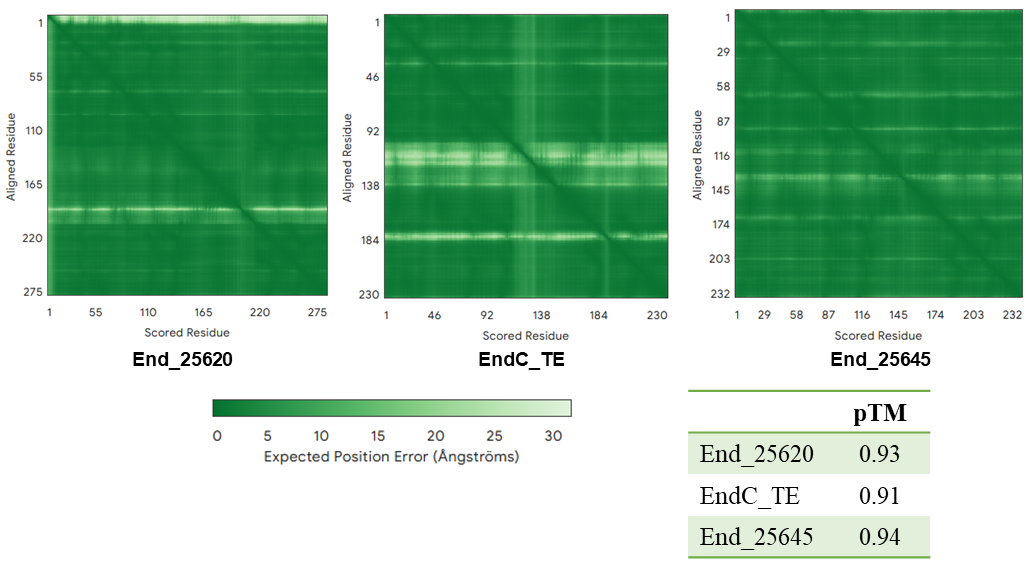


**Figure S4** Predicted aligned error (PAE) maps and predicted template modeling (pTM) scores of the three TEs predicted by AlphaFold3.


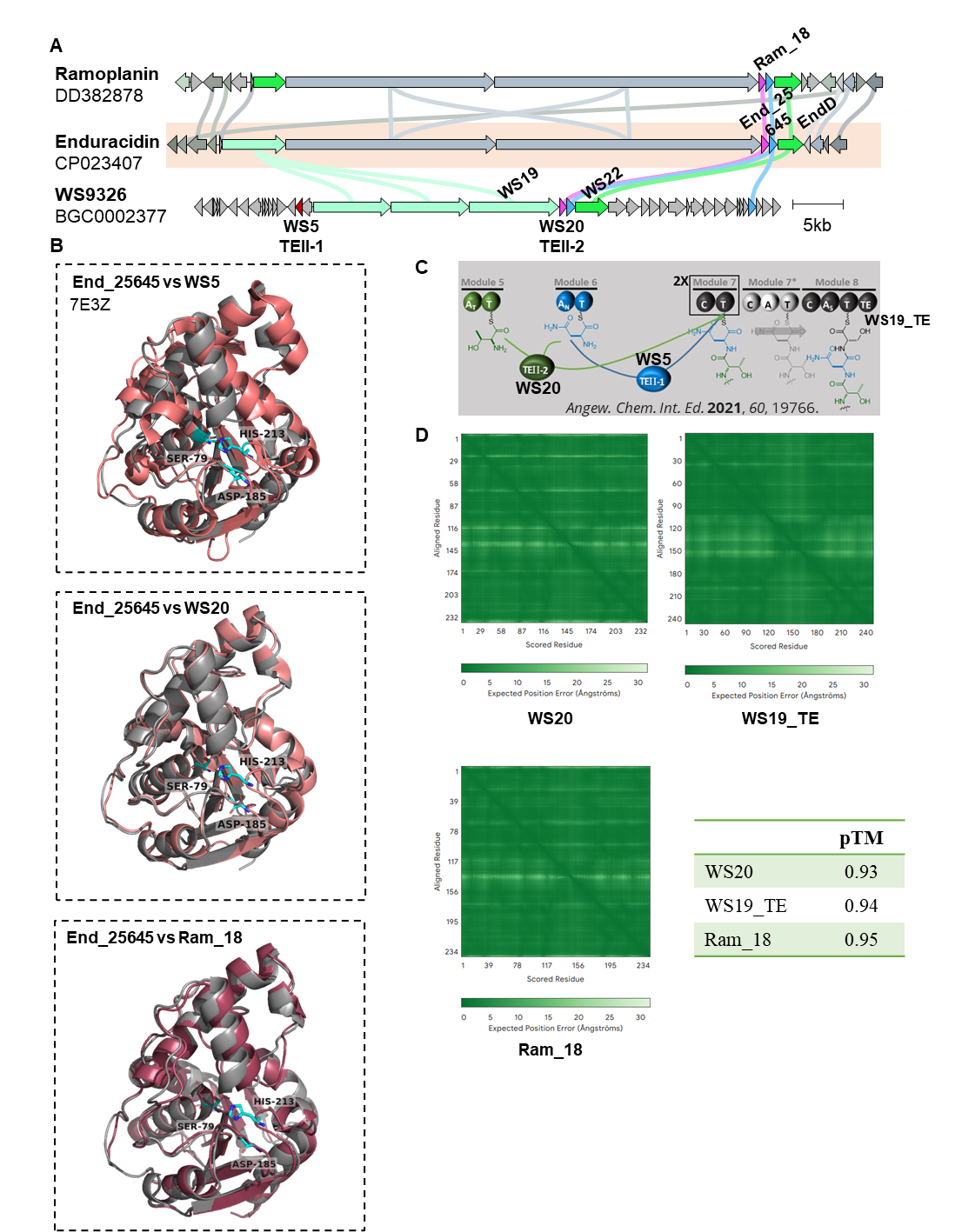


**Figure S5** Comparative gene cluster, structural, and confidence analysis of End_25645 with related TEIIs from ramoplanin and WS9326 biosynthetic gene clusters.

(A) Comparative genomic alignment of biosynthetic gene clusters from enduracidin, ramoplanin and WS9326. (B) Structural superposition of End_25645 with TEIIs WS20 and Ram_18. (C) Functional schematic of NRPS modules associated with WS20 and WS5 from WS9326 system. (D) AlphaFold3-predicted PAE maps and pTM values of the WS20, WS19_TE, and Ram_18 models. The crystal structure of WS5 was obtained from the RCSB Protein Data Bank (PDB) (PDB code: 7E3Z).


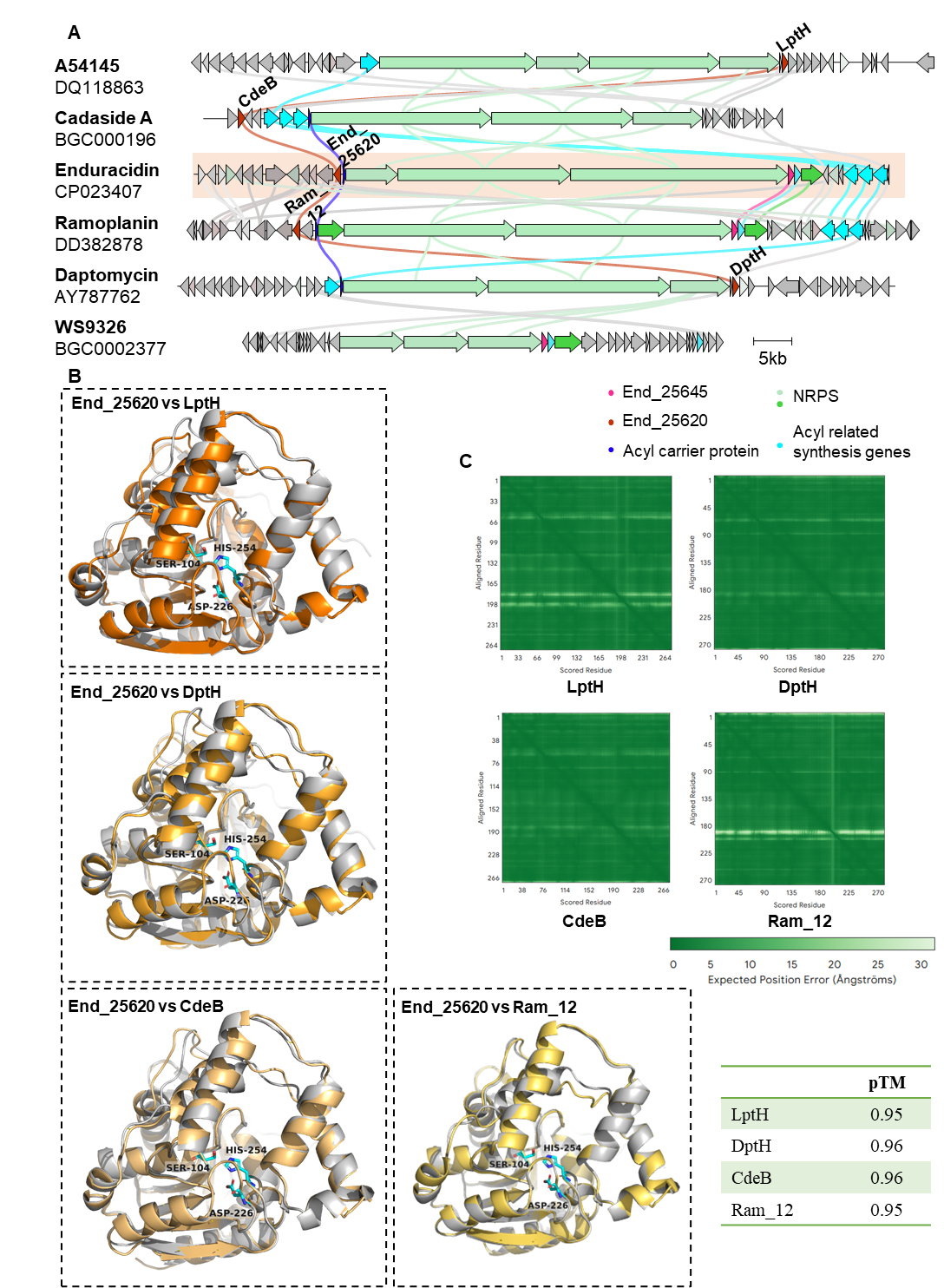


**Figure S6** Comparative gene cluster, structural, and confidence analysis of End_25620 with related TEIIs from biosynthetic gene clusters.

(A) Comparative genomic alignment of biosynthetic gene clusters from enduracidin and related gene clusters. (B) Structural superposition of End_25620 with the TEIIs LptH, DptH, CdeB and Ram_12. (C) AlphaFold3-predicted PAE maps and pTM values of LptH, DptH, CdeB and Ram_12 models.


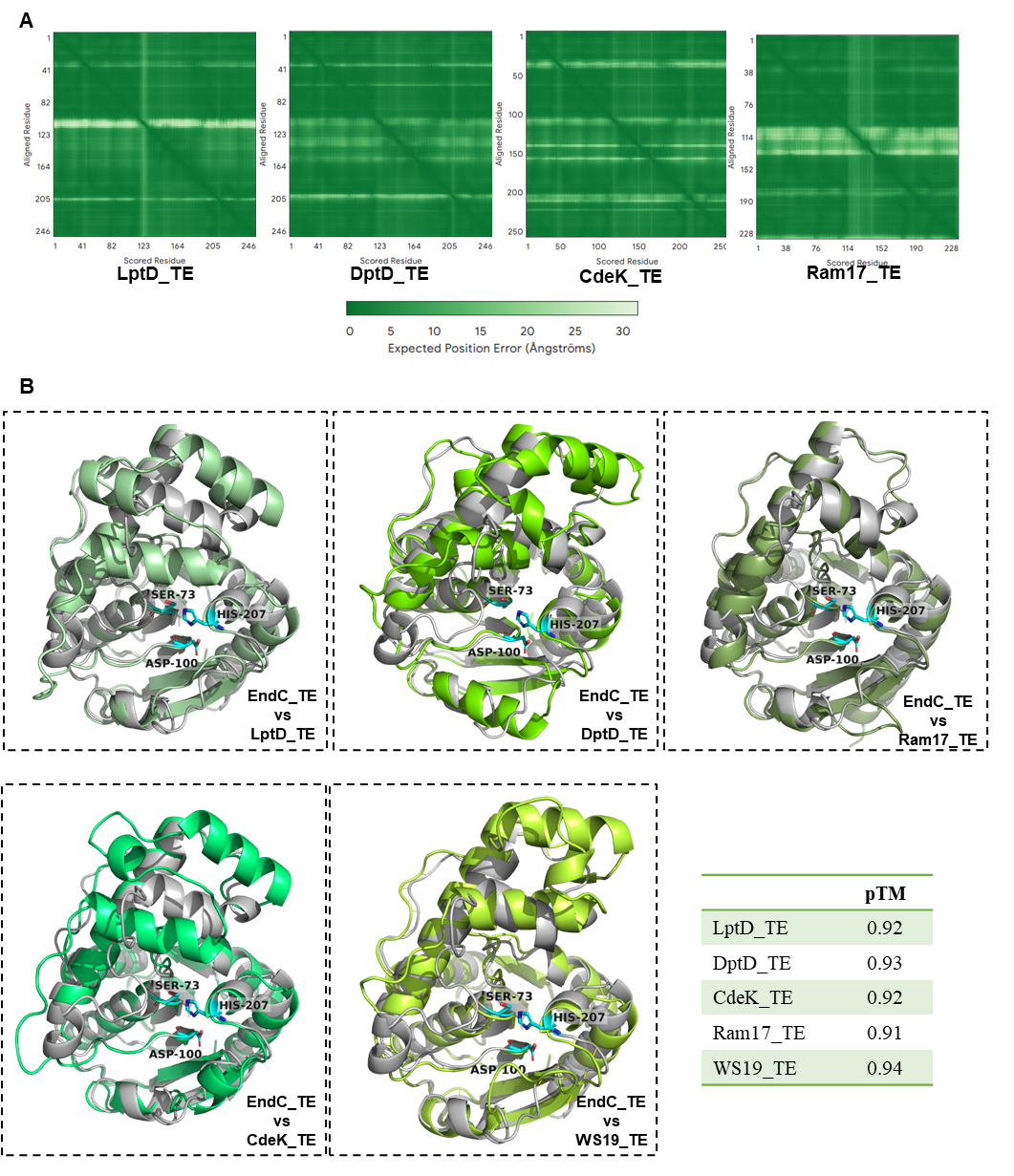


**Figure S7** Structural and confidence analysis of EndC_TE and related TEIs from biosynthetic gene clusters.

(A) AlphaFold3-predicted PAE maps and pTM values of the TEIs LptD_TE, DptD_TE, CdeK_TE, and Ram17_TE models. (B) Structural superposition of EndC_TE with related TEIs from biosynthetic gene clusters. The PAE map of WS19_TE is shown in Figure S5D.


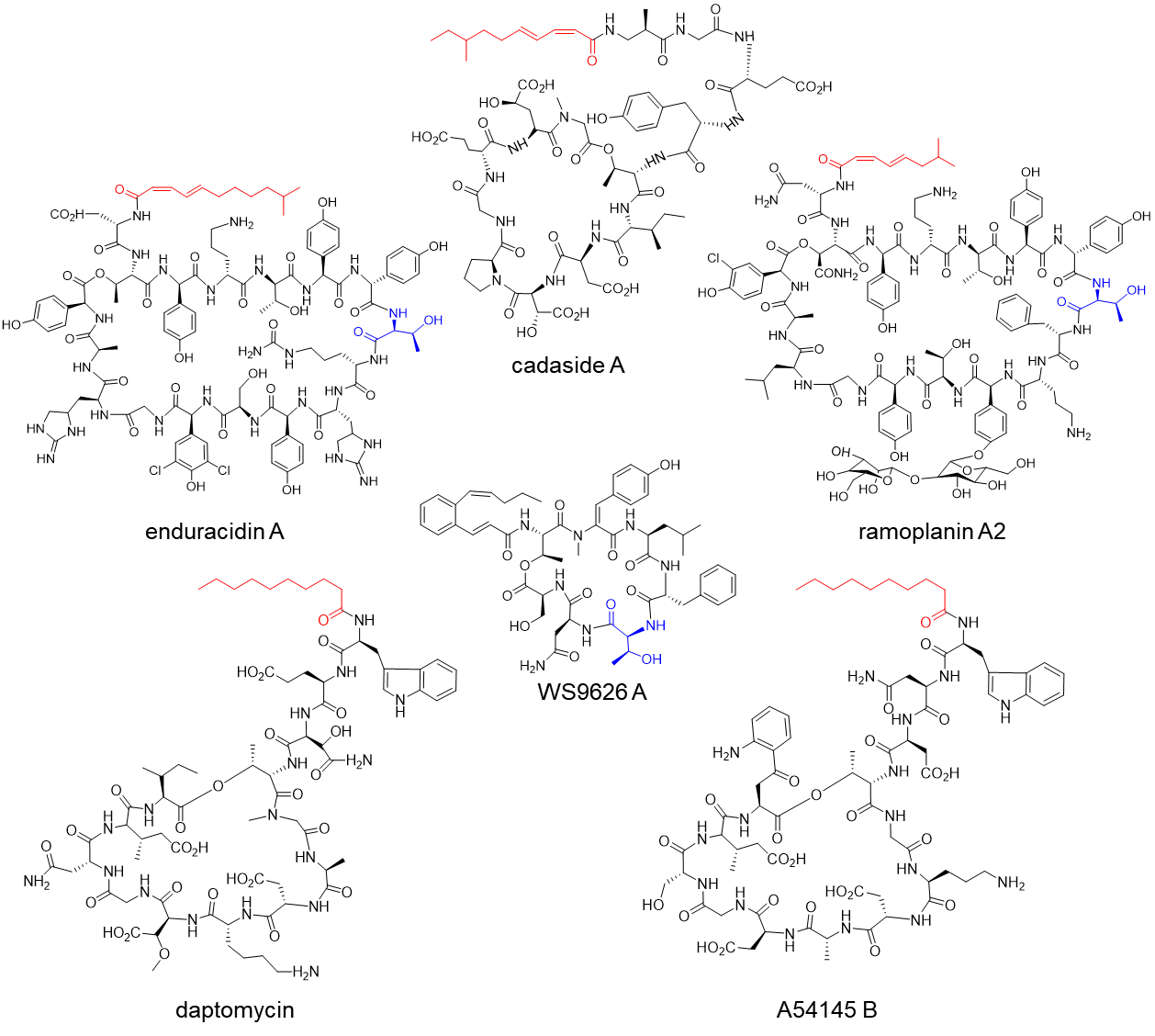


**Figure S8** Chemical structures of representative products from diverse NRPS biosynthetic gene clusters.

The fatty acyl chains are highlighted in red, and the L-allo-threonine residues are highlighted in blue.

Structures were adapted from the following references: cadaside A (*J. Am. Chem. Soc.*, 2019, 141, 9, 3910–3919), WS9626 A (*J. Org. Chem.*, 1993, 58, 1, 170–175), daptomycin and A54145 B (*Antimicrob. Agents Chemother.*, 2010, 54, 4, 1404–1413), and enduracidin A and ramoplanin A2 (*Microbiology*, 2015, 161, 7, 1338–1347).

**
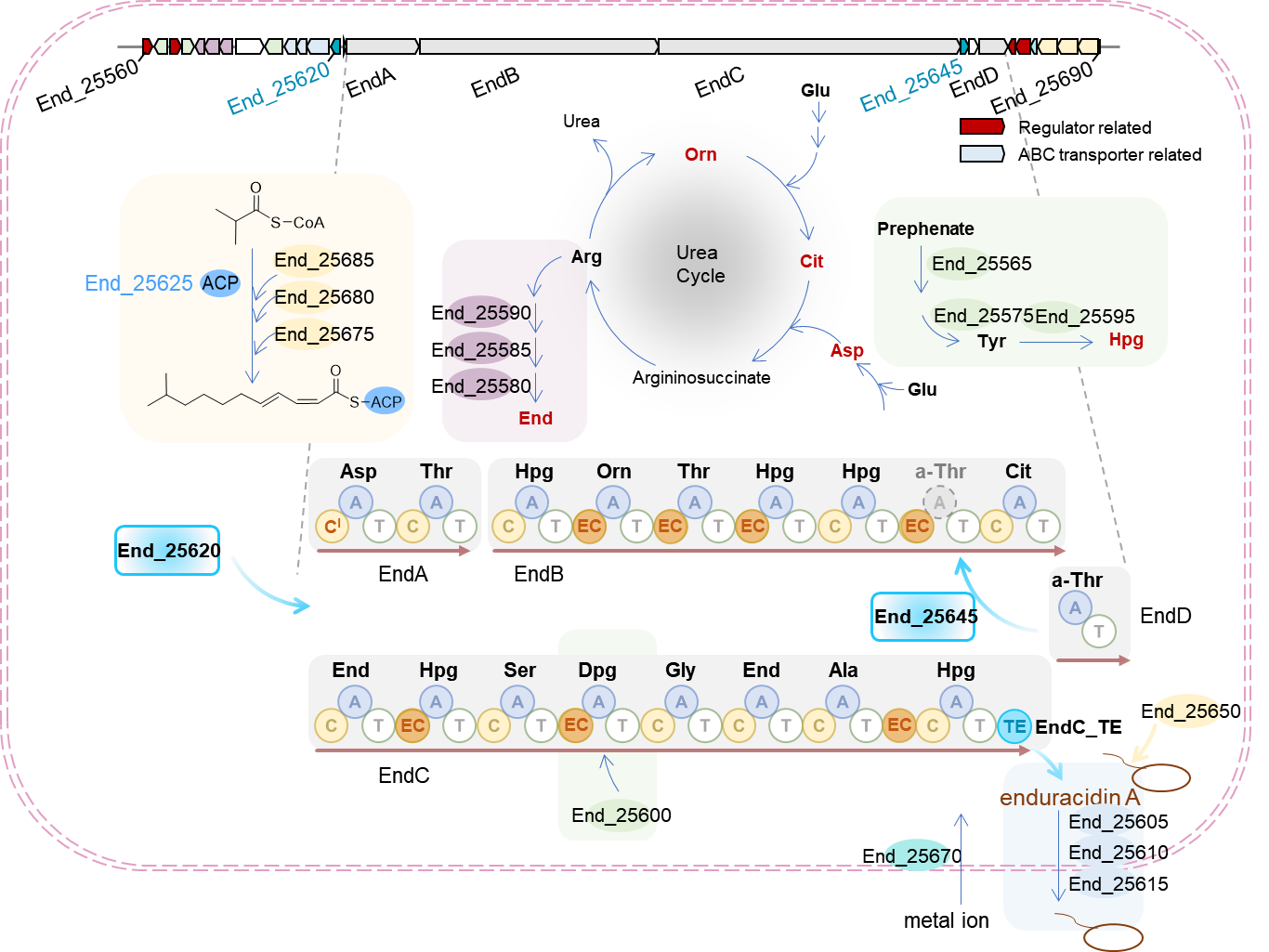
**

**Figure S9** Proposed biosynthetic pathway of enduracidin A.

This schematic illustrates the proposed biosynthetic pathway of enduracidin A, including NRPS-mediated peptide assembly, fatty acyl chain biosynthesis, and the formation of non-proteinogenic amino acids. All proteins shown are encoded within the enduracidin biosynthetic gene cluster, and the corresponding gene IDs are listed in Supplementary Table S8. Arrows indicate the proposed involvement of the corresponding proteins in the biosynthetic processes and do not necessarily represent individual catalytic steps. The pathway was proposed based on bioinformatic analysis and sequence homology.


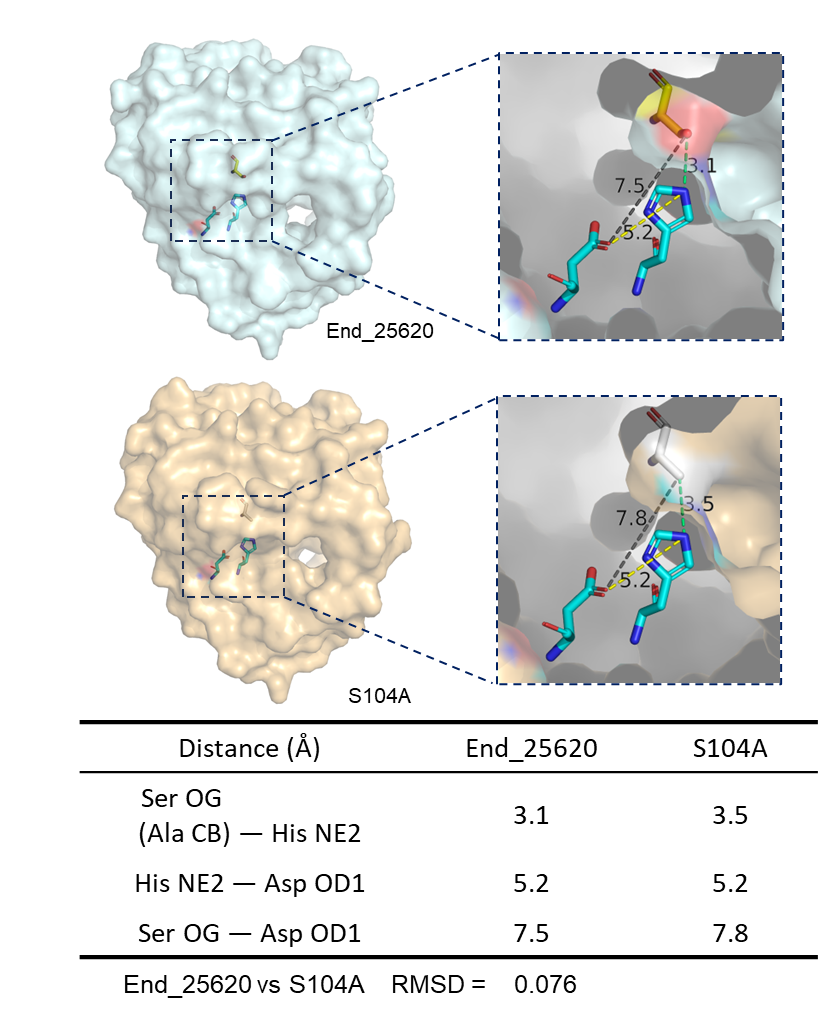


**Figure S10** Comparison of interatomic distances within the catalytic triads of End_25620 and the mutant S104A.


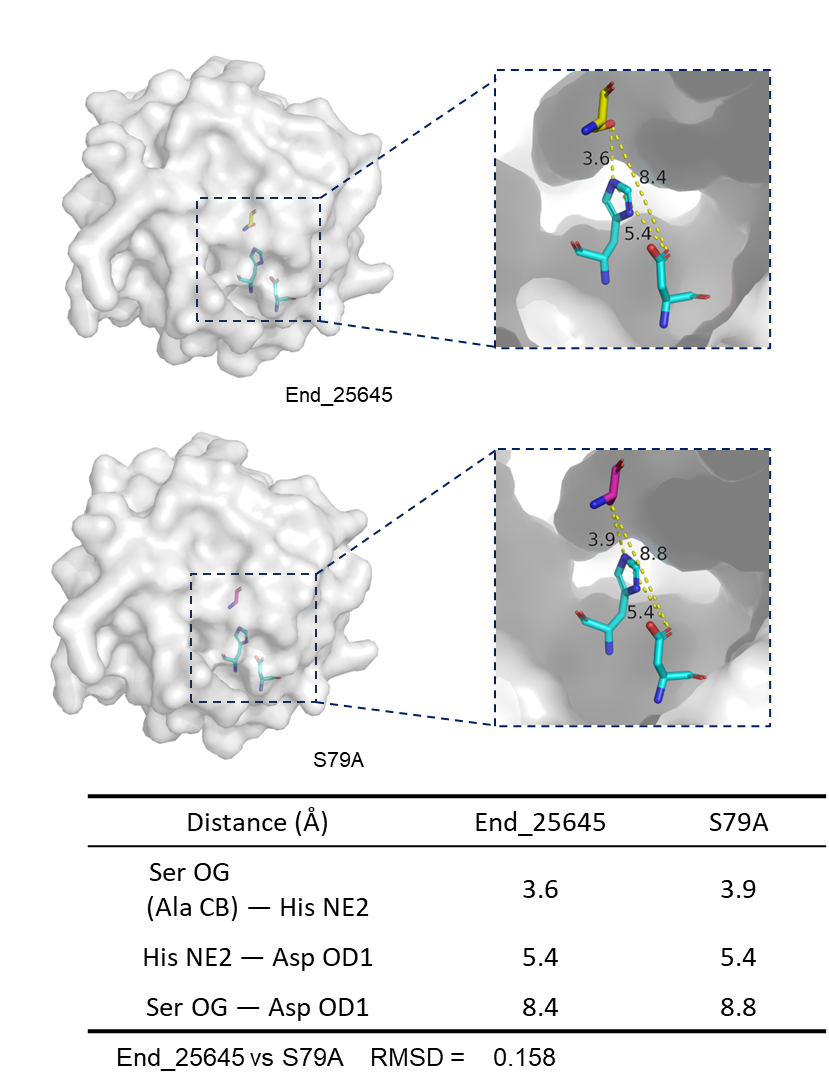


**Figure S11** Comparison of interatomic distances within the catalytic triads of End_25645 and the mutant S79A.


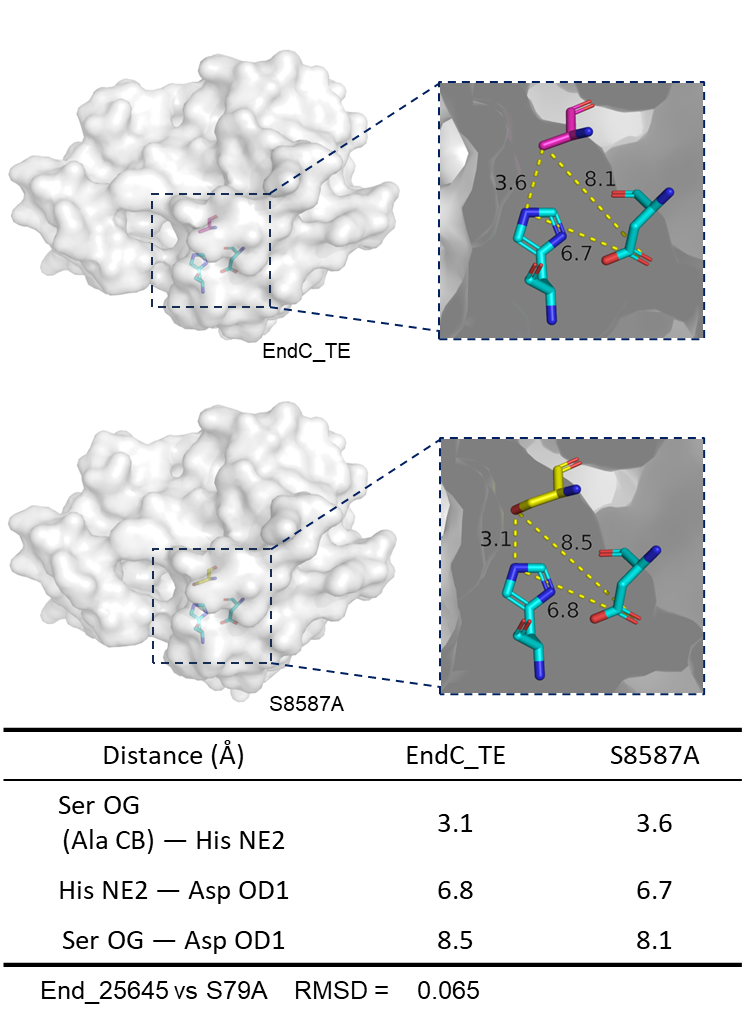


**Figure S12** Comparison of interatomic distances within the catalytic triads of EndC_TE and the mutant S8587A.


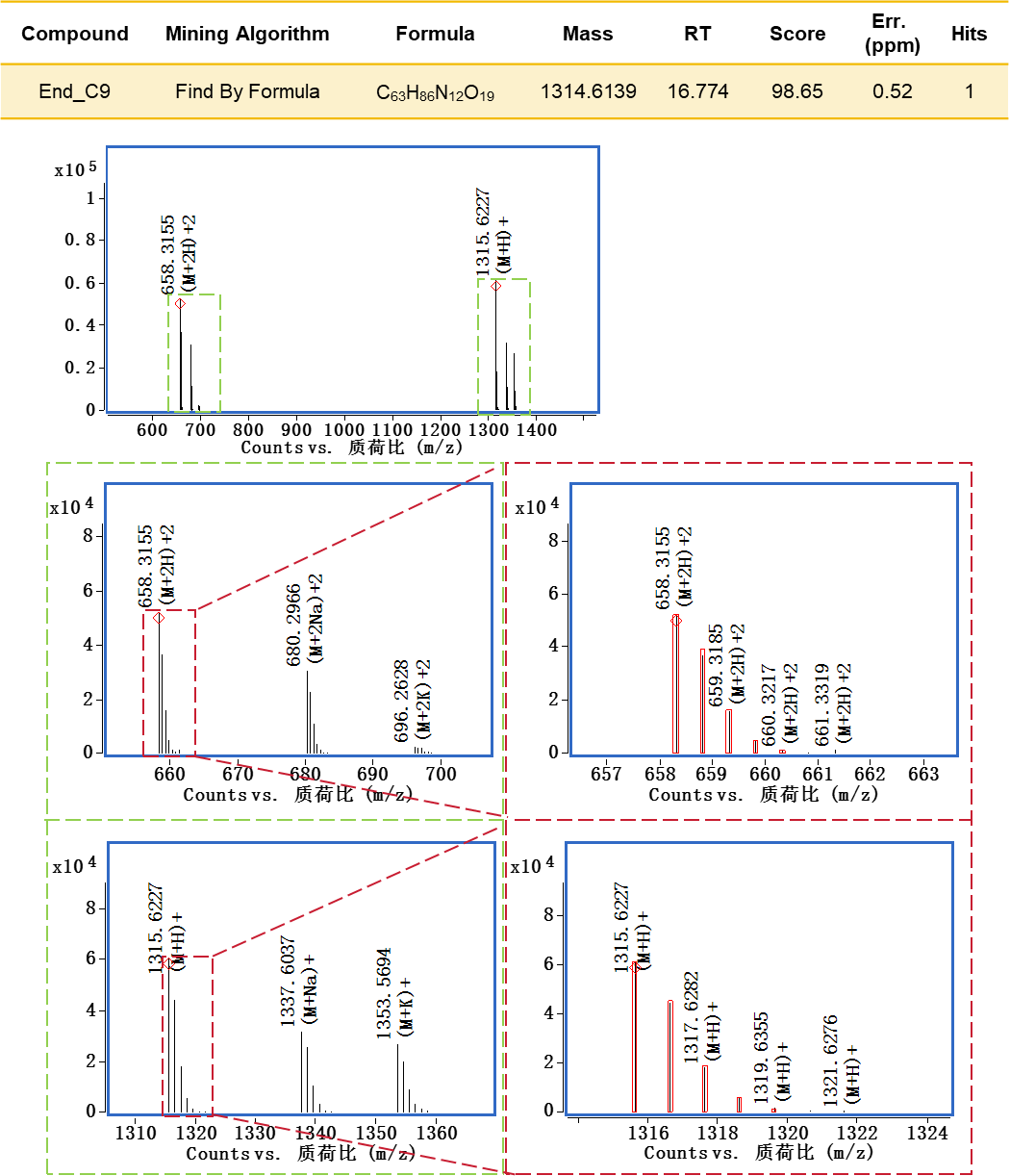


**Figure S13** LC–HRMS and isotopic distribution analysis of End_C9.

Detection of the target compound End_C9 by the Find-by-Formula algorithm, with representative MS and isotopic distribution analysis. Shown are (i) the full mass spectrum with annotated charge states, (ii) magnified views of selected charge states, and (iii) overlays of the observed and predicted isotopic distributions.


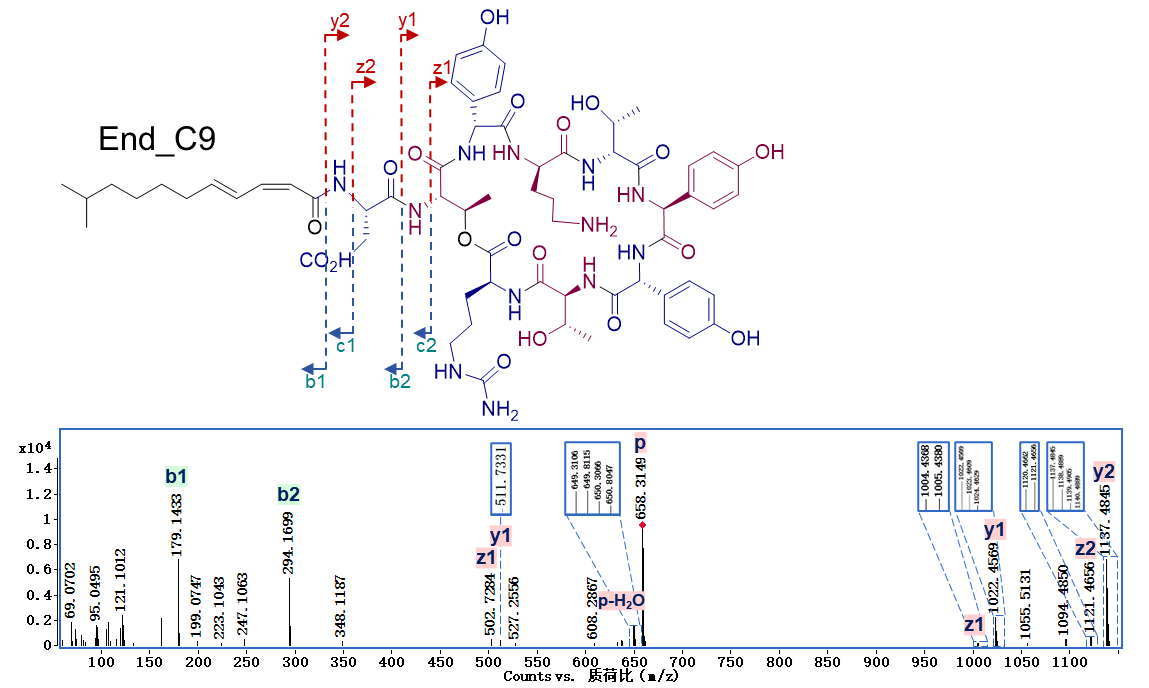


**Figure S14** LC–HRMS/MS spectrum of End_C9.

Representative MS/MS fragmentation spectrum of End_C9 with annotated fragment ions. The corresponding fragment formulas, theoretical *m/z*, observed *m/z*, and mass errors are summarized in Table S10.

End_C9 CID = 12.5 eV; prec. *m/z* 658.3155 [*z*=2]


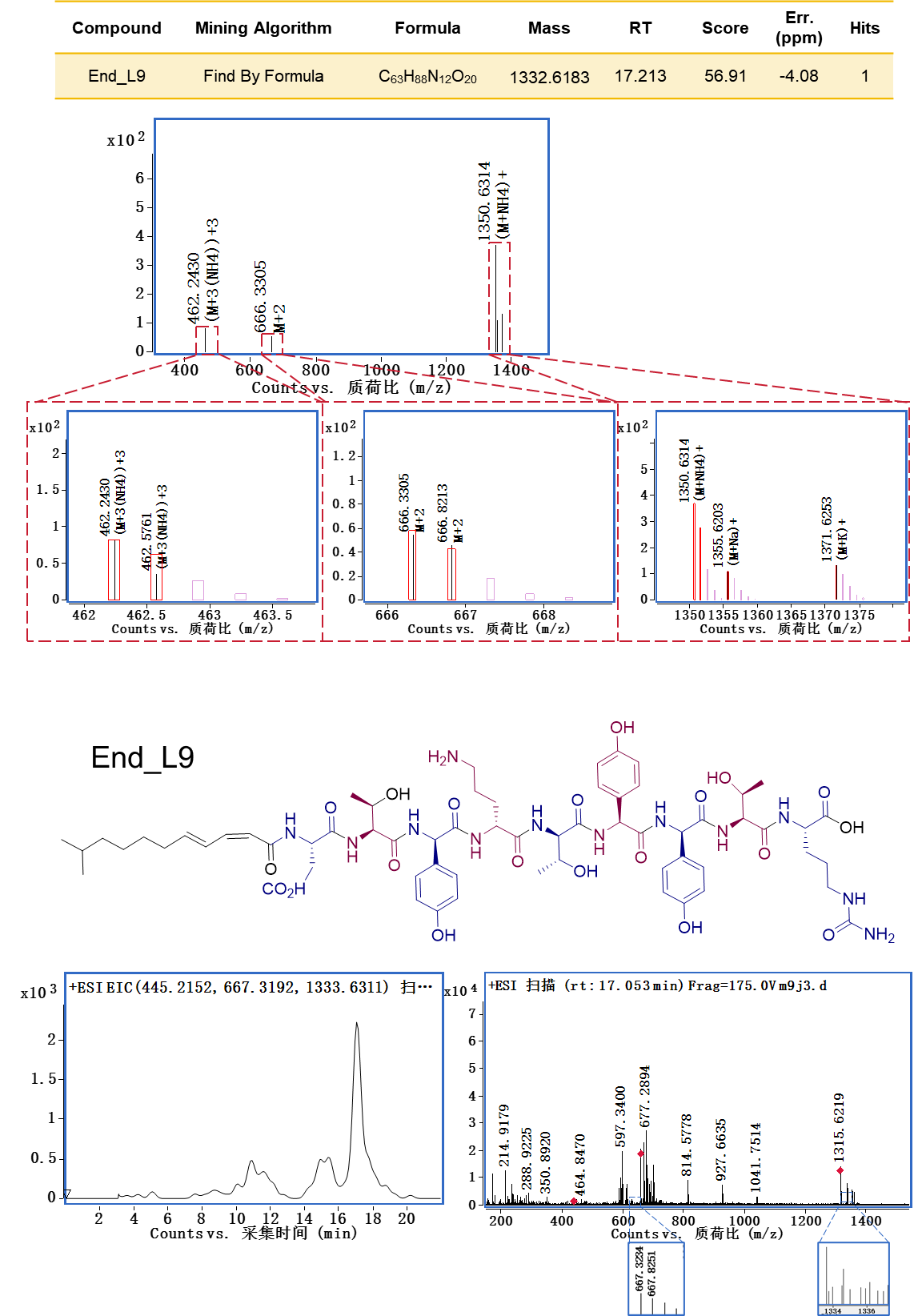


**Figure S15** LC–HRMS spectrum and isotopic distribution analysis of End_L9.

Due to low abundance (~10^2^ counts), only a limited number of ions were observed. The upper panel shows adduct ions[M+NH_4_]^+^, [M+2H]^2+^, [M+3(NH_4_)]^3+^, [M+Na]^+^, and [M+K]^+^ identified by the Find-by-Formula algorithm, with isotopic distributions matching the predicted patterns (calculated/observed m/z and mass errors in Table S9). The lower panel presents EIC traces for three charge states of End_L9, showing peaks at the expected retention time, along with the corresponding MS spectrum highlighting three red-marked ions. Due to low signal intensity, no MS/MS spectrum was obtained.


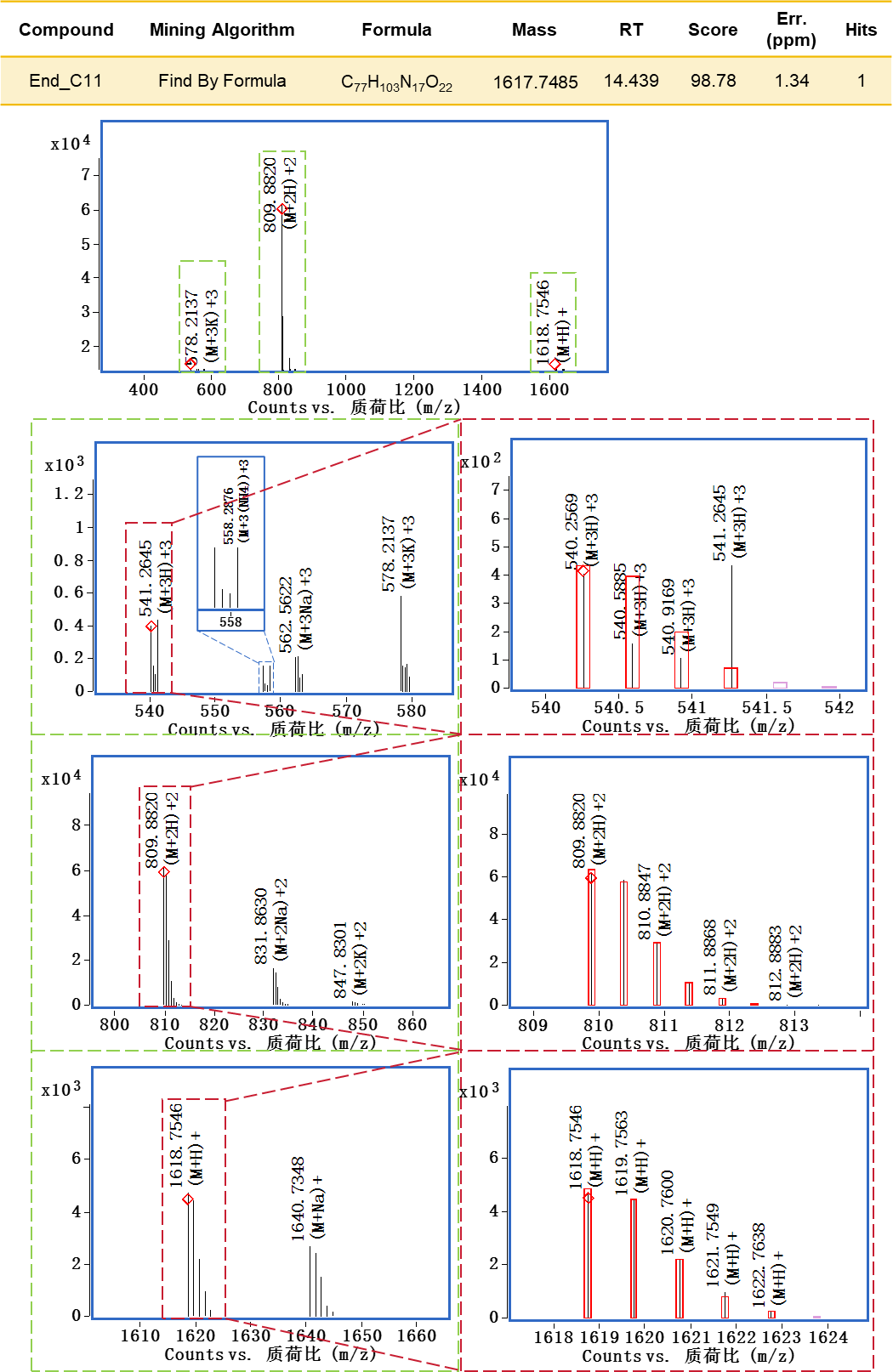


**Figure S16** LC–HRMS and isotopic distribution analysis of End_C11.

Detection of the target compound End_C11 by the Find-by-Formula algorithm, with representative MS and isotopic distribution analysis. Shown are (i) the full mass spectrum with annotated charge states, (ii) magnified views of selected charge states, and (iii) overlays of the observed and predicted isotopic distributions.


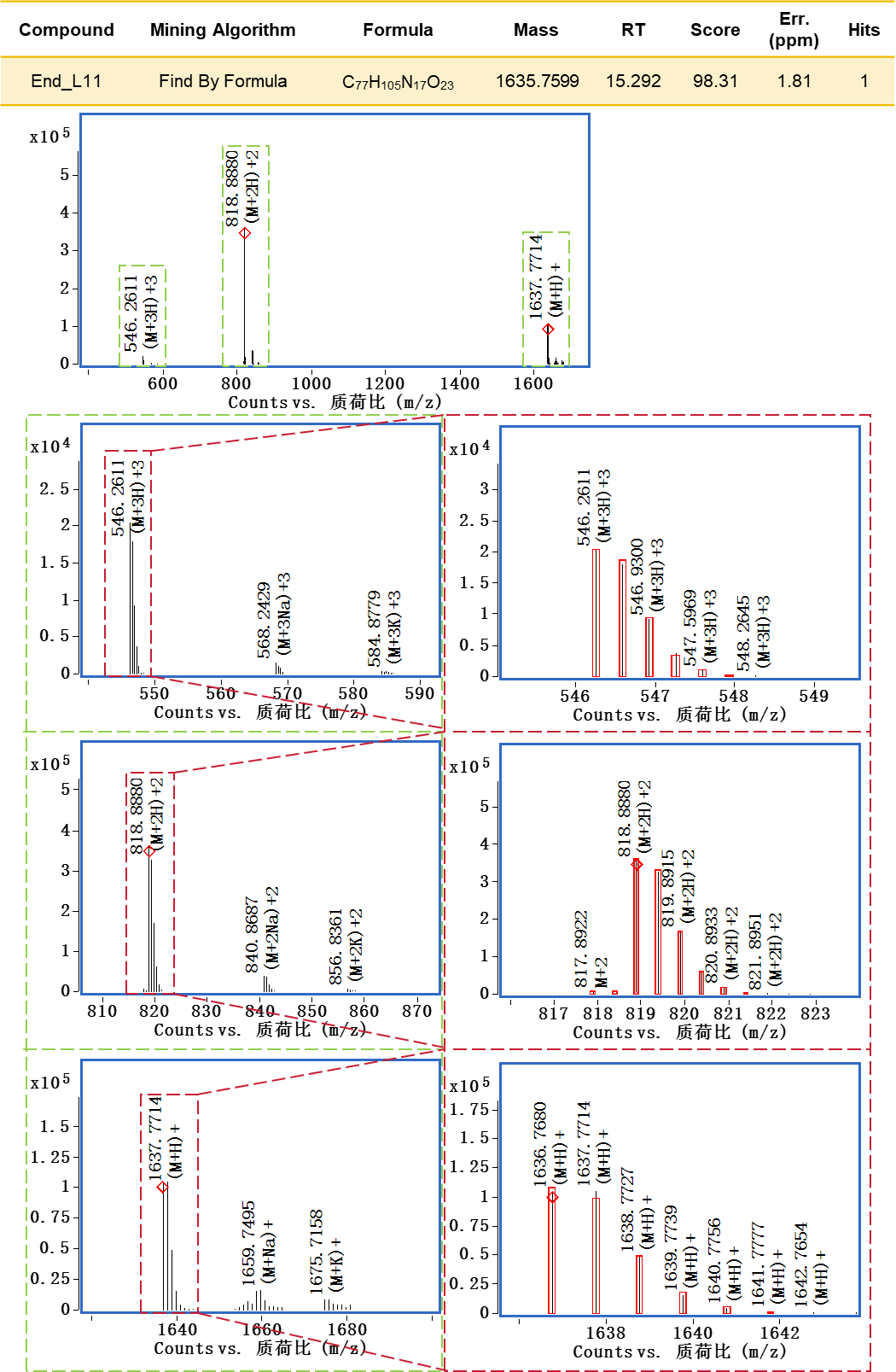


**Figure S17** LC–HRMS and isotopic distribution analysis of End_L11.

Detection of the target compound End_L11 by the Find-by-Formula algorithm, with representative MS and isotopic distribution analysis. Shown are (i) the full mass spectrum with annotated charge states, (ii) magnified views of selected charge states, and (iii) overlays of the observed and predicted isotopic distributions.


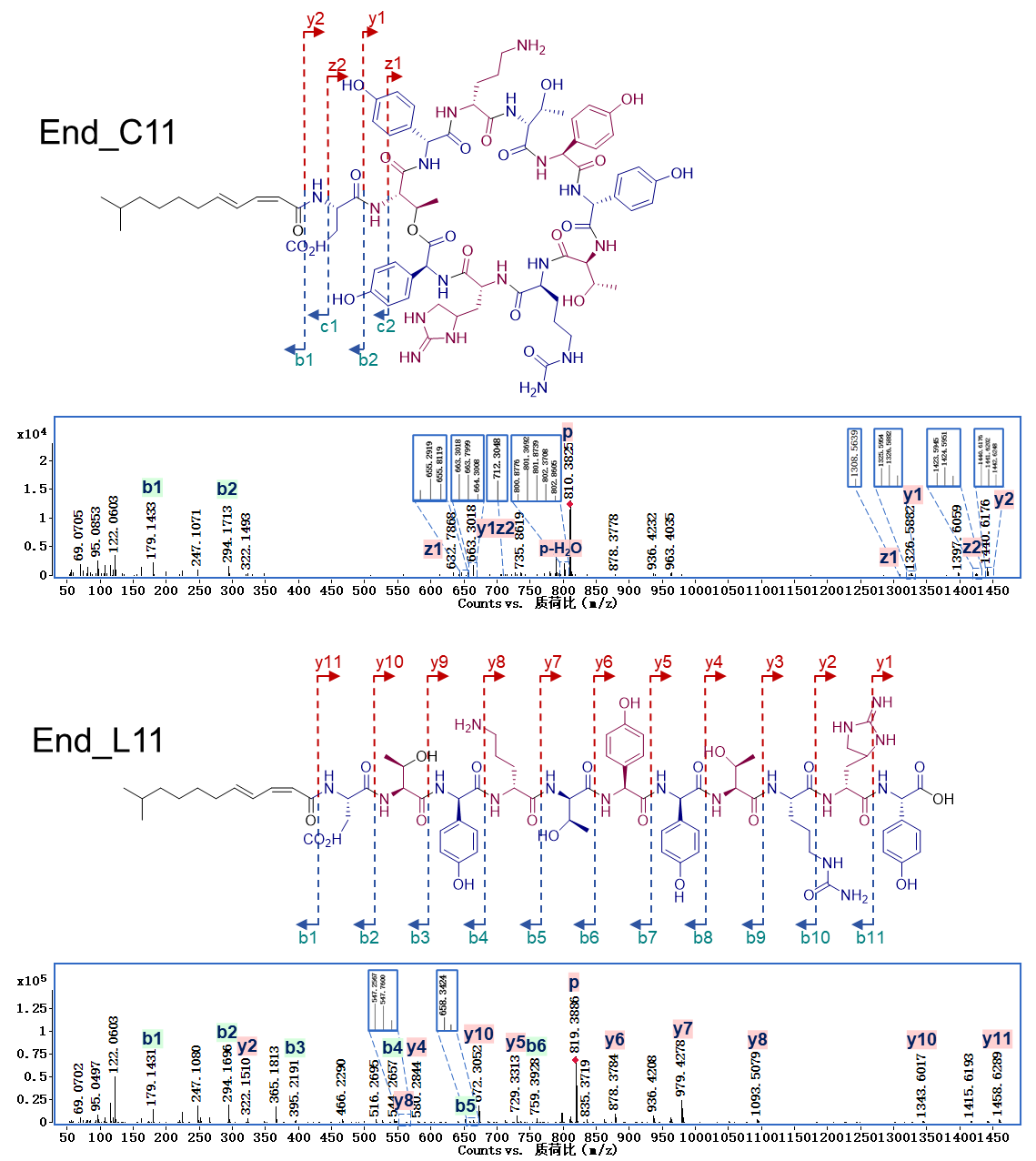


**Figure S18** LC–HRMS/MS spectra of End_C11 and End_L11.

Representative MS/MS fragmentation spectra of End_C11 and End_L11 with annotated fragment ions. The corresponding fragment formulas, theoretical *m/z*, observed *m/z*, and mass errors are summarized in Tables S11–S12.

End_C11 CID = 28.0 eV; prec. *m/z* 809.8820 [*z*=2]

End_L11 CID = 28.0 eV; prec. *m/z* 818.8862 [*z*=2]


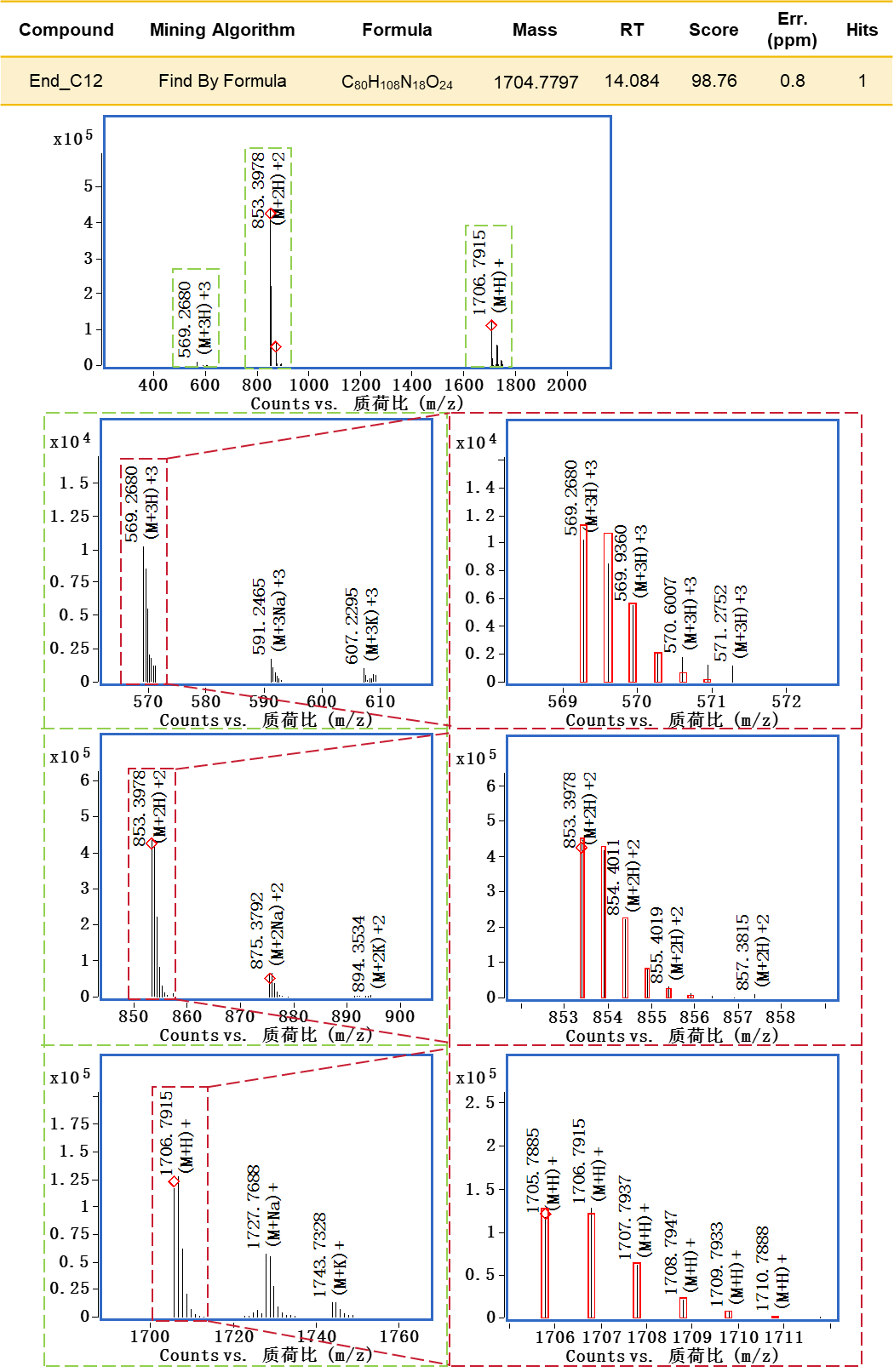


**Figure S19** LC–HRMS and isotopic distribution analysis of End_C12.

Detection of the target compound End_C12 by the Find-by-Formula algorithm, with representative MS and isotopic distribution analysis. Shown are (i) the full mass spectrum with annotated charge states, (ii) magnified views of selected charge states, and (iii) overlays of the observed and predicted isotopic distributions.


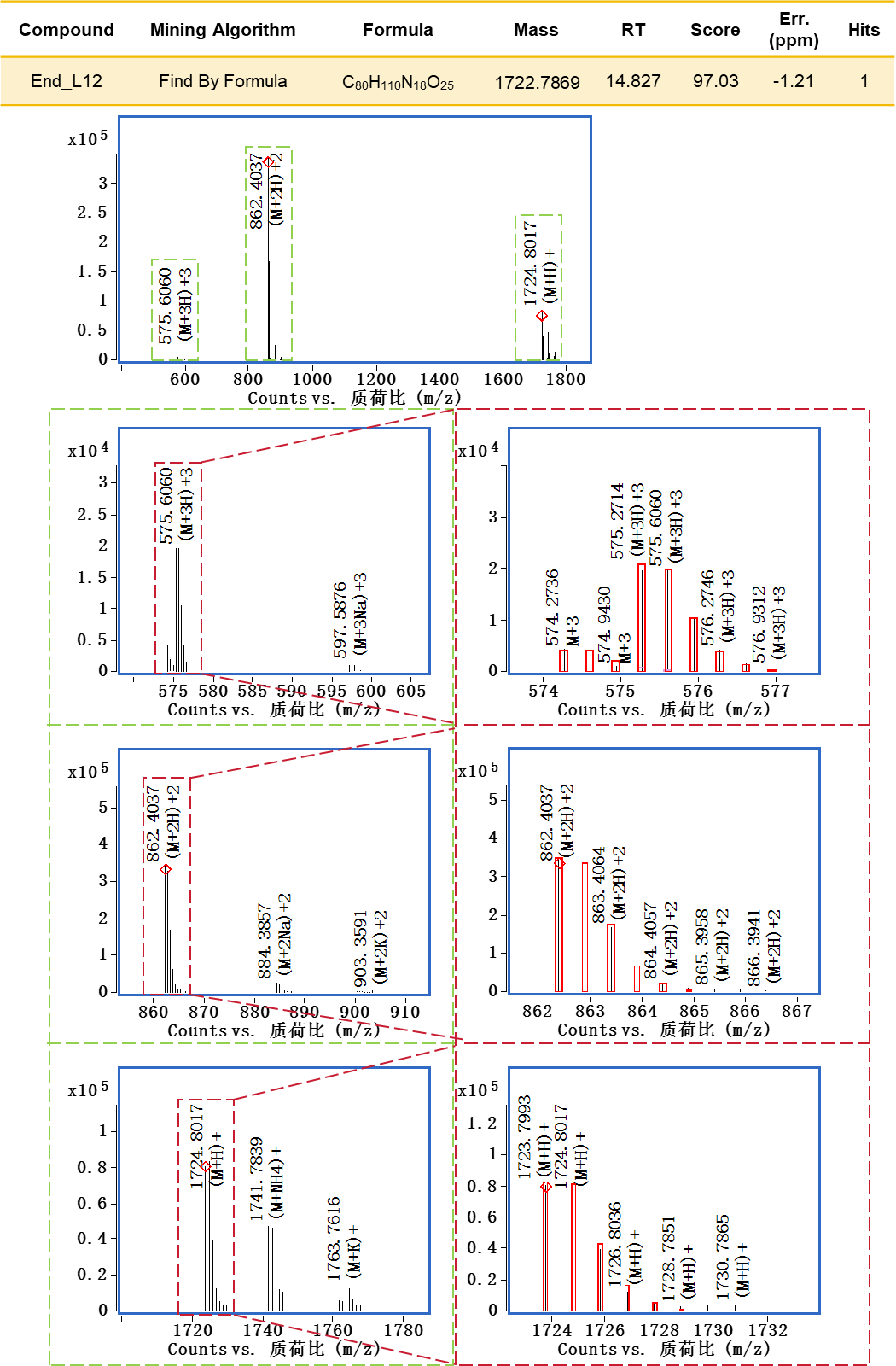


**Figure S20** LC–HRMS and isotopic distribution analysis of End_L12.

Detection of the target compound End_L12 by the Find-by-Formula algorithm, with representative MS and isotopic distribution analysis. Shown are (i) the full mass spectrum with annotated charge states, (ii) magnified views of selected charge states, and (iii) overlays of the observed and predicted isotopic distributions.


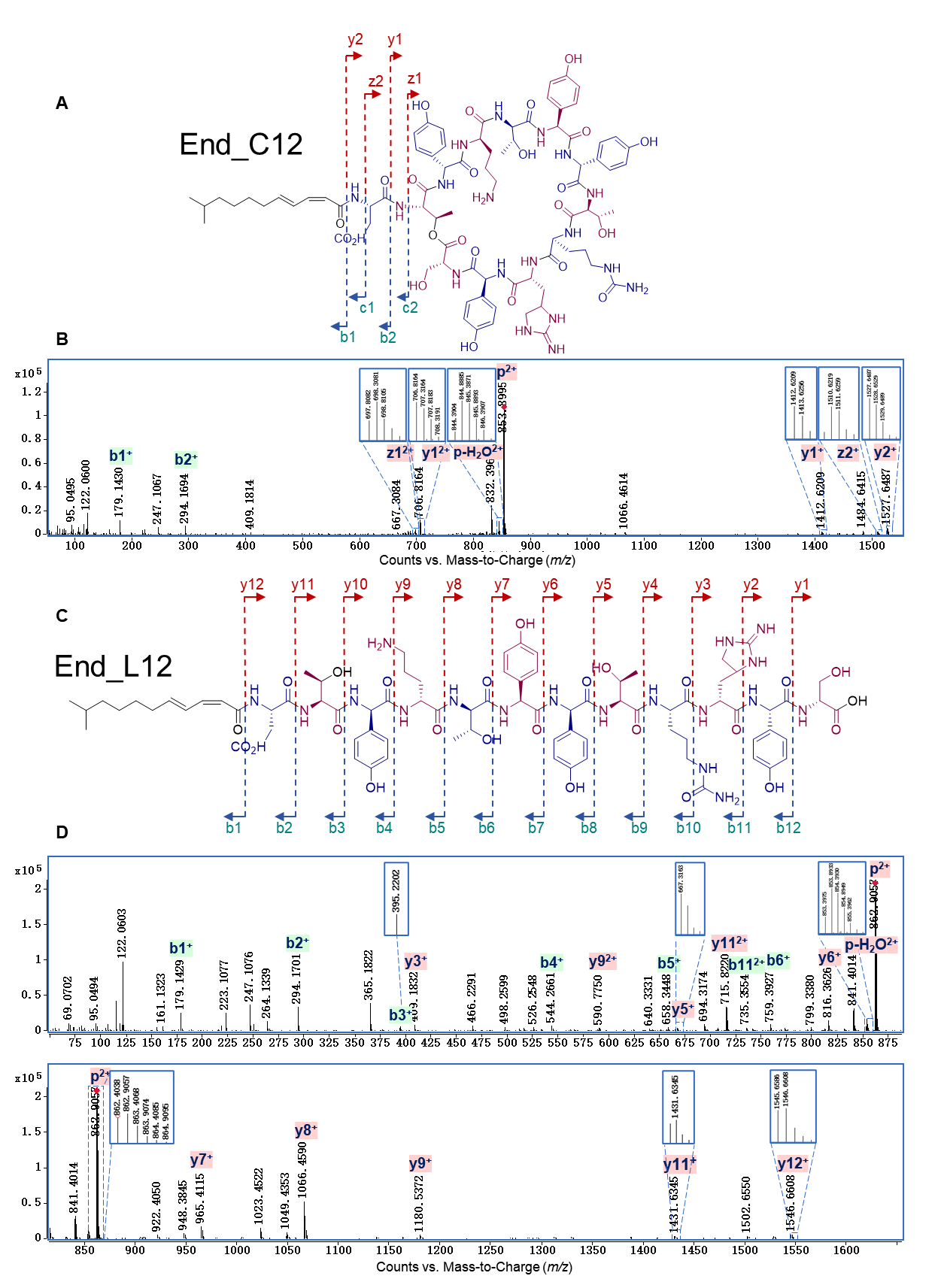


**Figure S21** LC–HRMS/MS spectra of End_C12 and End_L12.

Representative MS/MS fragmentation spectra of End_C12 and End_L12 with annotated fragment ions. The corresponding fragment formulas, theoretical *m/z*, observed *m/z*, and mass errors are summarized in Tables S13–S14.

End_C12 CID = 27.0 eV; prec. *m/z* 853.3982 [*z*=2]

End_L12 CID = 28.0 eV; prec. *m/z* 862.4038 [*z*=2]


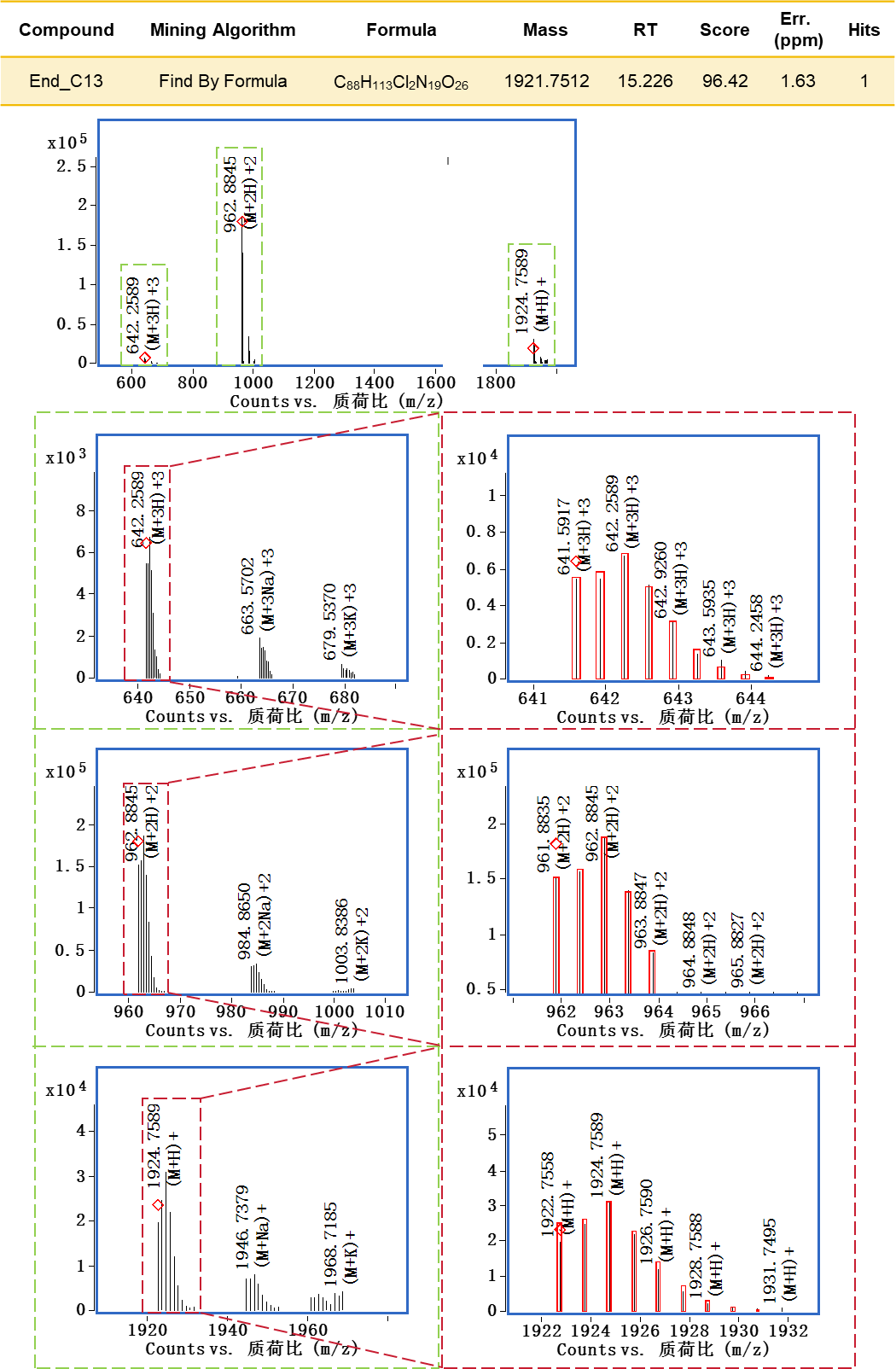


**Figure S22** LC–HRMS and isotopic distribution analysis of End_C13.

Detection of the target compound End_C13 by the Find-by-Formula algorithm, with representative MS and isotopic distribution analysis. Shown are (i) the full mass spectrum with annotated charge states, (ii) magnified views of selected charge states, and (iii) overlays of the observed and predicted isotopic distributions.


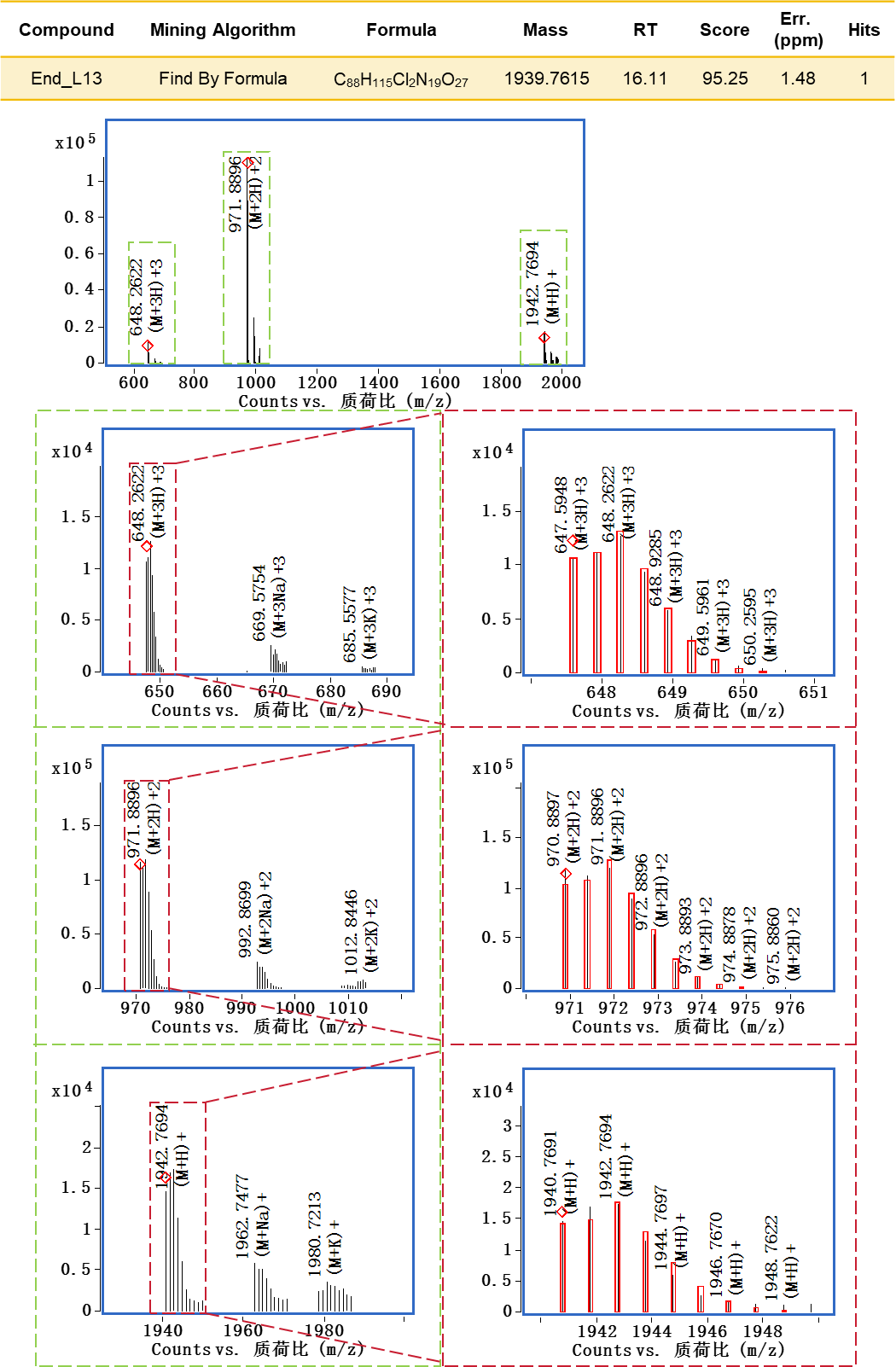


**Figure S23** LC–HRMS and isotopic distribution analysis of End_L13.

Detection of the target compound End_L13 by the Find-by-Formula algorithm, with representative MS and isotopic distribution analysis. Shown are (i) the full mass spectrum with annotated charge states, (ii) magnified views of selected charge states, and (iii) overlays of the observed and predicted isotopic distributions.


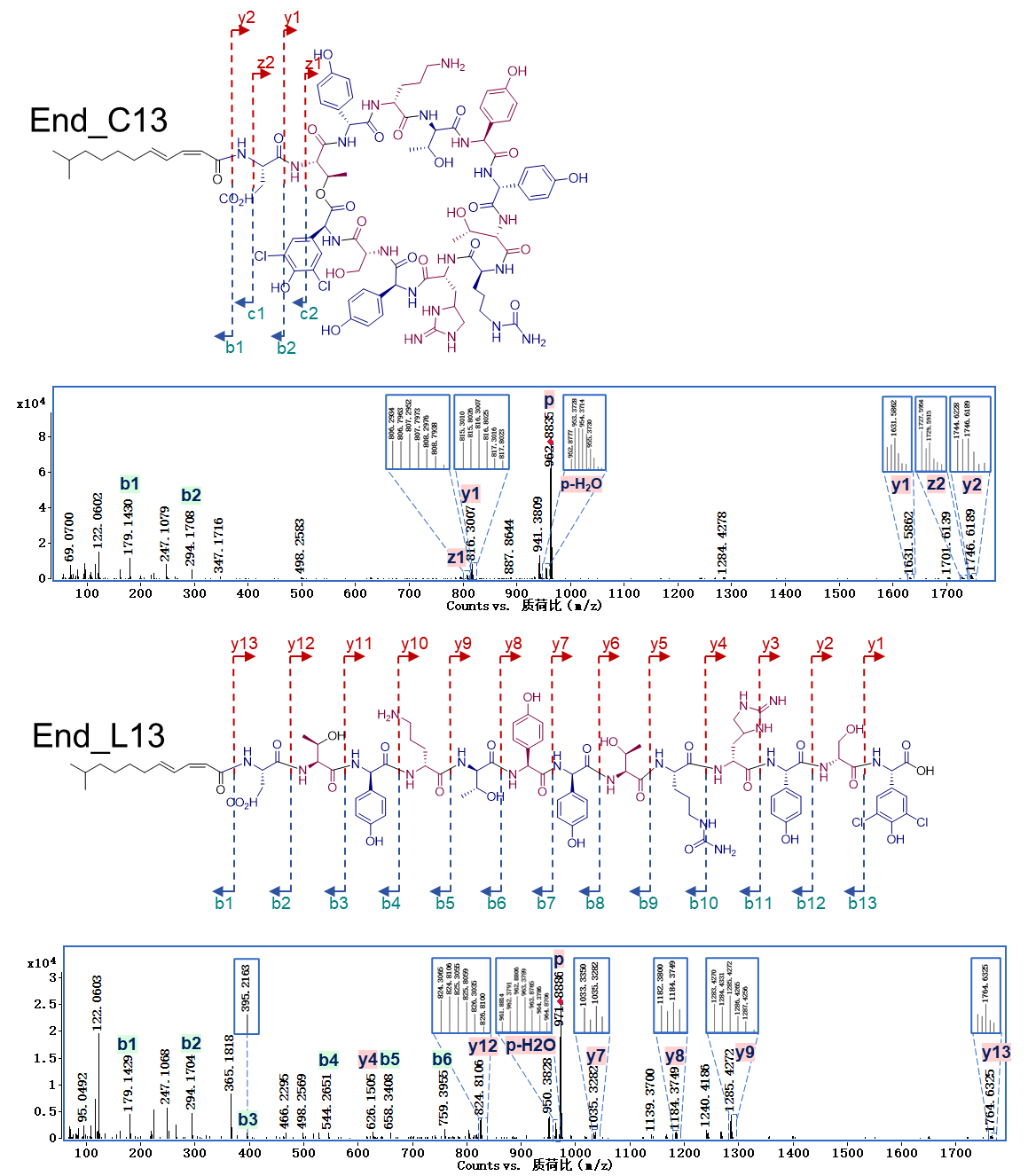


**Figure S24** LC–HRMS/MS spectra of End_C13 and End_L13.

Representative MS/MS fragmentation spectra of End_C13 and End_L13 with annotated fragment ions. The corresponding fragment formulas, theoretical *m/z*, observed *m/z*, and mass errors are summarized in Tables S15–S16.

End_C13 CID = 30.0 eV; prec. *m/z* 961.8835 [*z*=2]

End_L13 CID = 32.0 eV; prec. *m/z* 970.8897 [*z*=2]

**
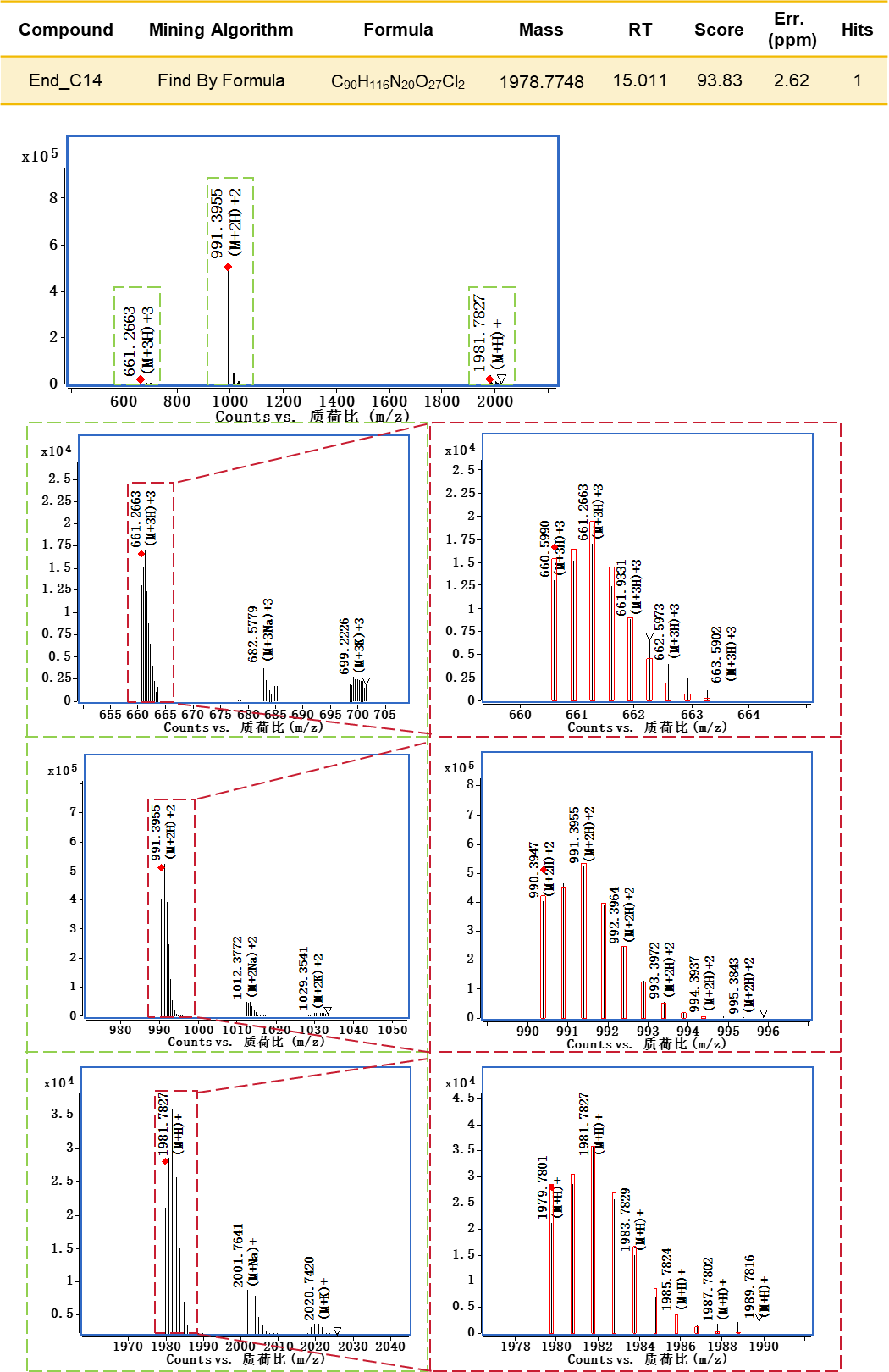
**

**Figure S25** LC–HRMS and isotopic distribution analysis of End_C14.

Detection of the target compound End_C14 by the Find-by-Formula algorithm, with representative MS and isotopic distribution analysis. Shown are (i) the full mass spectrum with annotated charge states, (ii) magnified views of selected charge states, and (iii) overlays of the observed and predicted isotopic distributions.

**
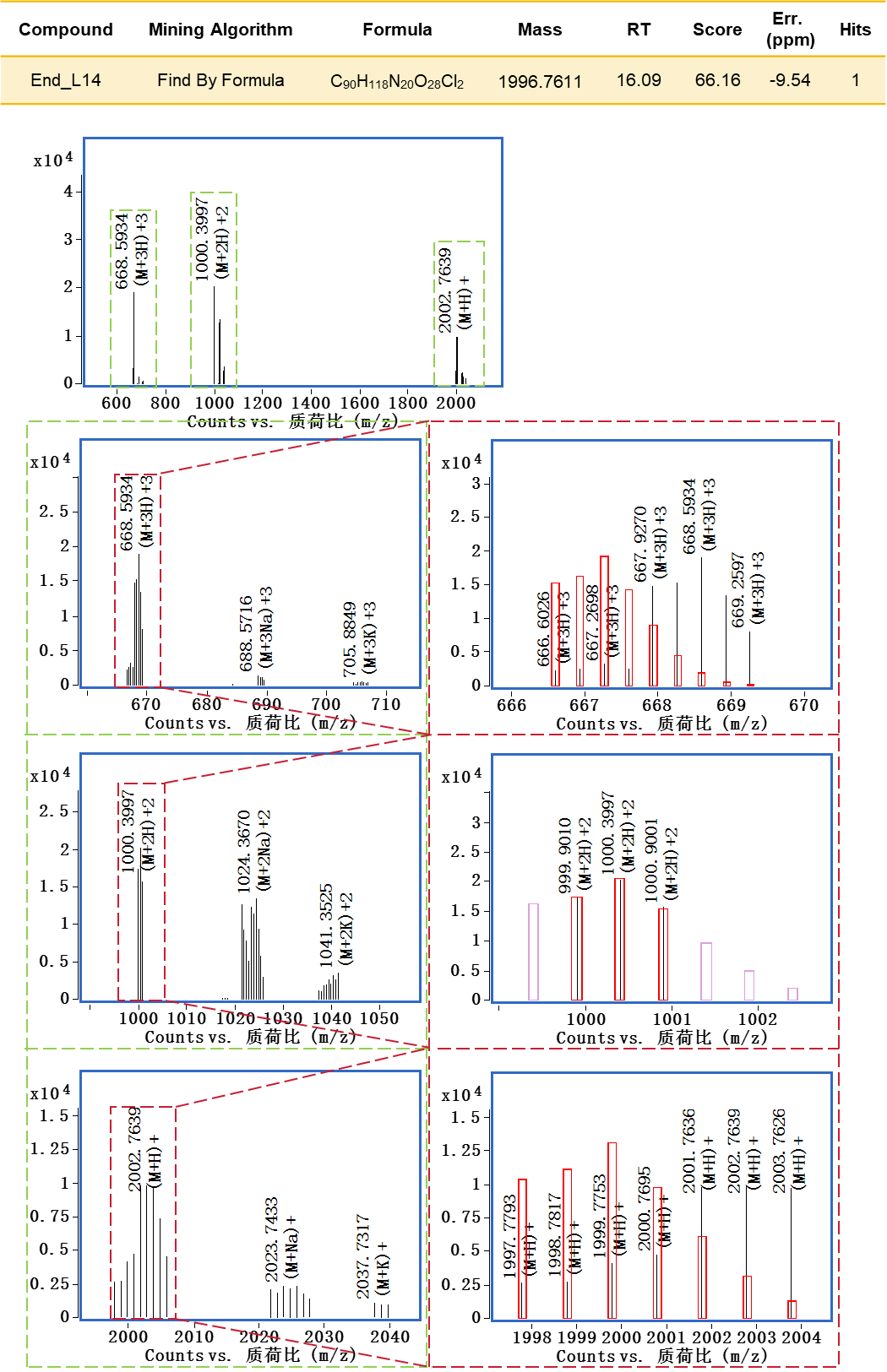
**

**Figure S26** LC–HRMS and isotopic distribution analysis of End_L14.

Detection of the target compound End_L14 by the Find-by-Formula algorithm, with representative MS and isotopic distribution analysis. Shown are (i) the full mass spectrum with annotated charge states, (ii) magnified views of selected charge states, and (iii) overlays of the observed and predicted isotopic distributions.


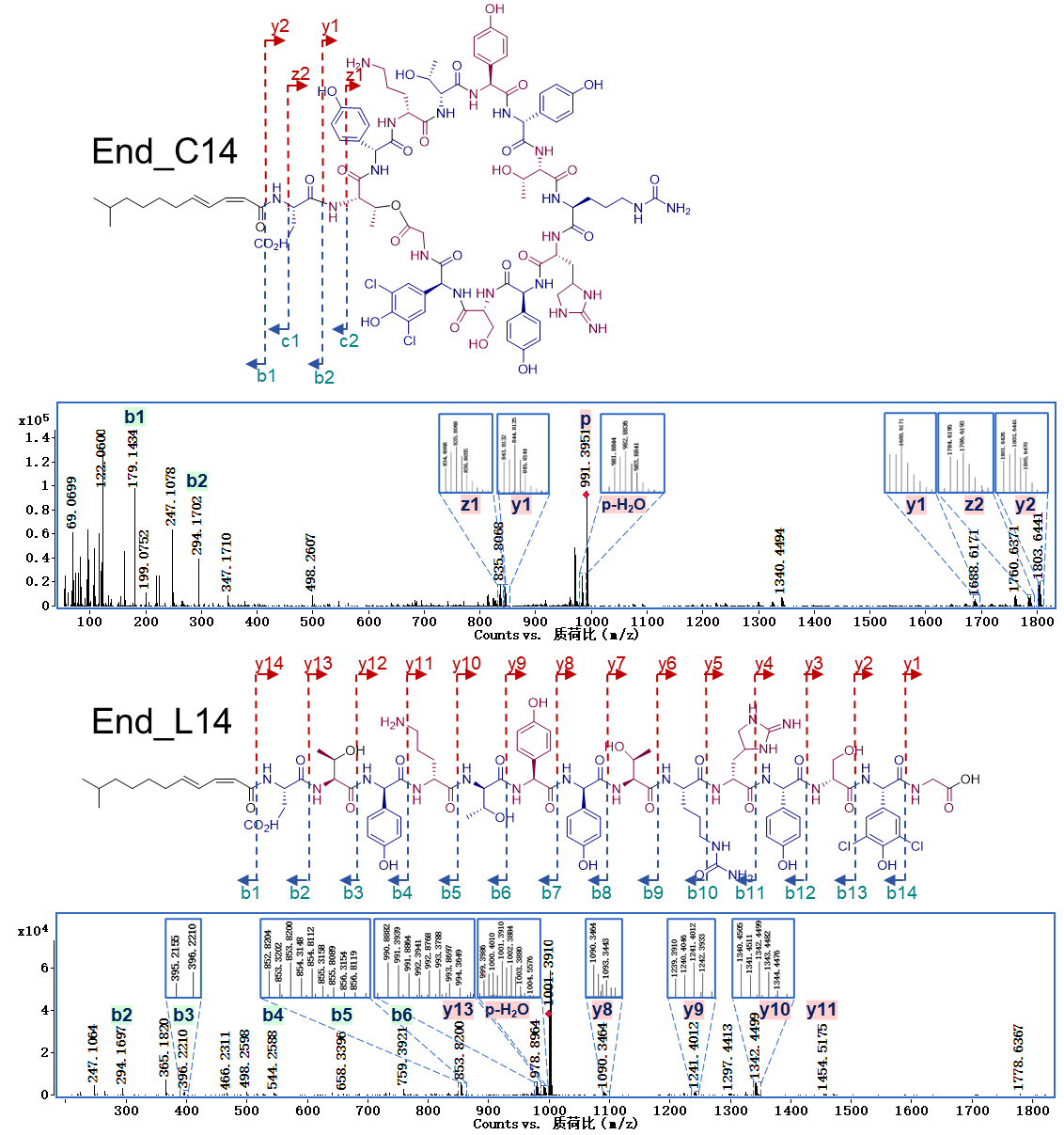


**Figure S27** LC–HRMS/MS spectra of End_C14 and End_L14.

Representative MS/MS fragmentation spectra of End_C14 and End_L14 with annotated fragment ions. The corresponding fragment formulas, theoretical *m/z*, observed *m/z*, and mass errors are summarized in Tables S17–S18.

End_C14 CID = 40.0 eV; prec. *m/z* 990.3947 [*z*=2]

End_L14 CID = 40.0 eV; prec. *m/z* 999.3986 [*z*=2]


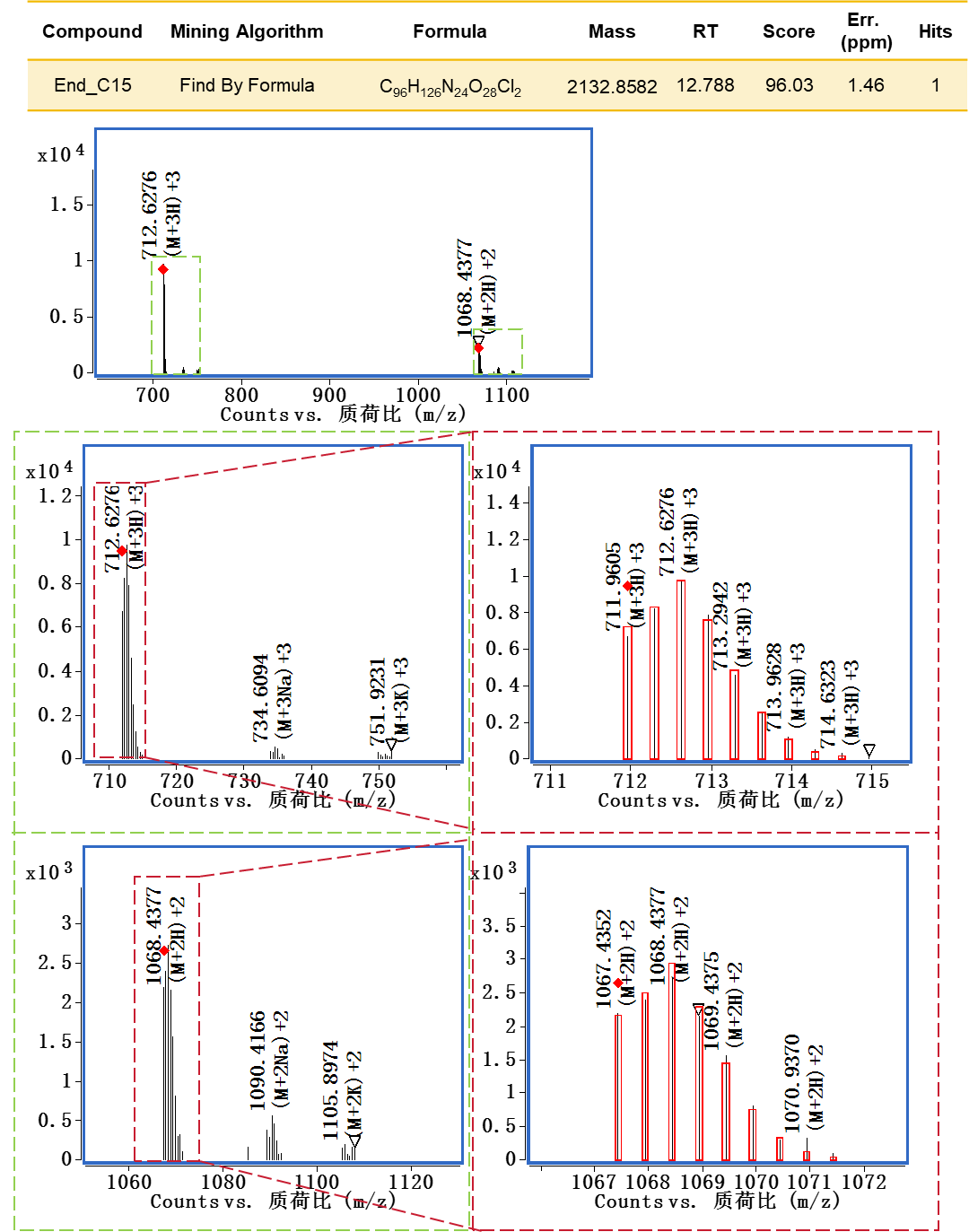


**Figure S28** LC–HRMS and isotopic distribution analysis of End_C15.

Detection of the target compound End_C15 by the Find-by-Formula algorithm, with representative MS and isotopic distribution analysis. Shown are (i) the full mass spectrum with annotated charge states, (ii) magnified views of selected charge states, and (iii) overlays of the observed and predicted isotopic distributions.


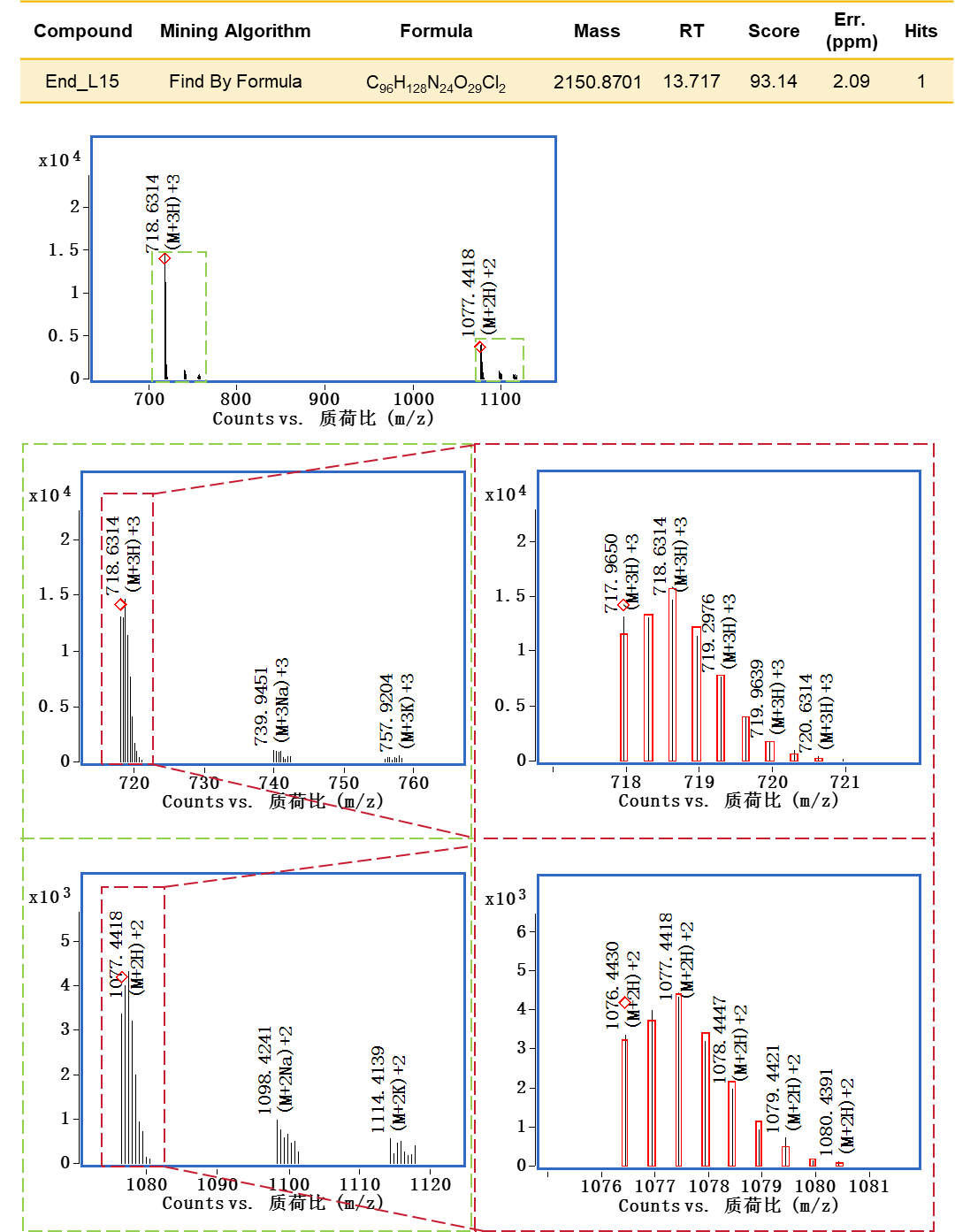


**Figure S29** LC–HRMS and isotopic distribution analysis of End_L15.

Detection of the target compound End_L15 by the Find-by-Formula algorithm, with representative MS and isotopic distribution analysis. Shown are (i) the full mass spectrum with annotated charge states, (ii) magnified views of selected charge states, and (iii) overlays of the observed and predicted isotopic distributions.


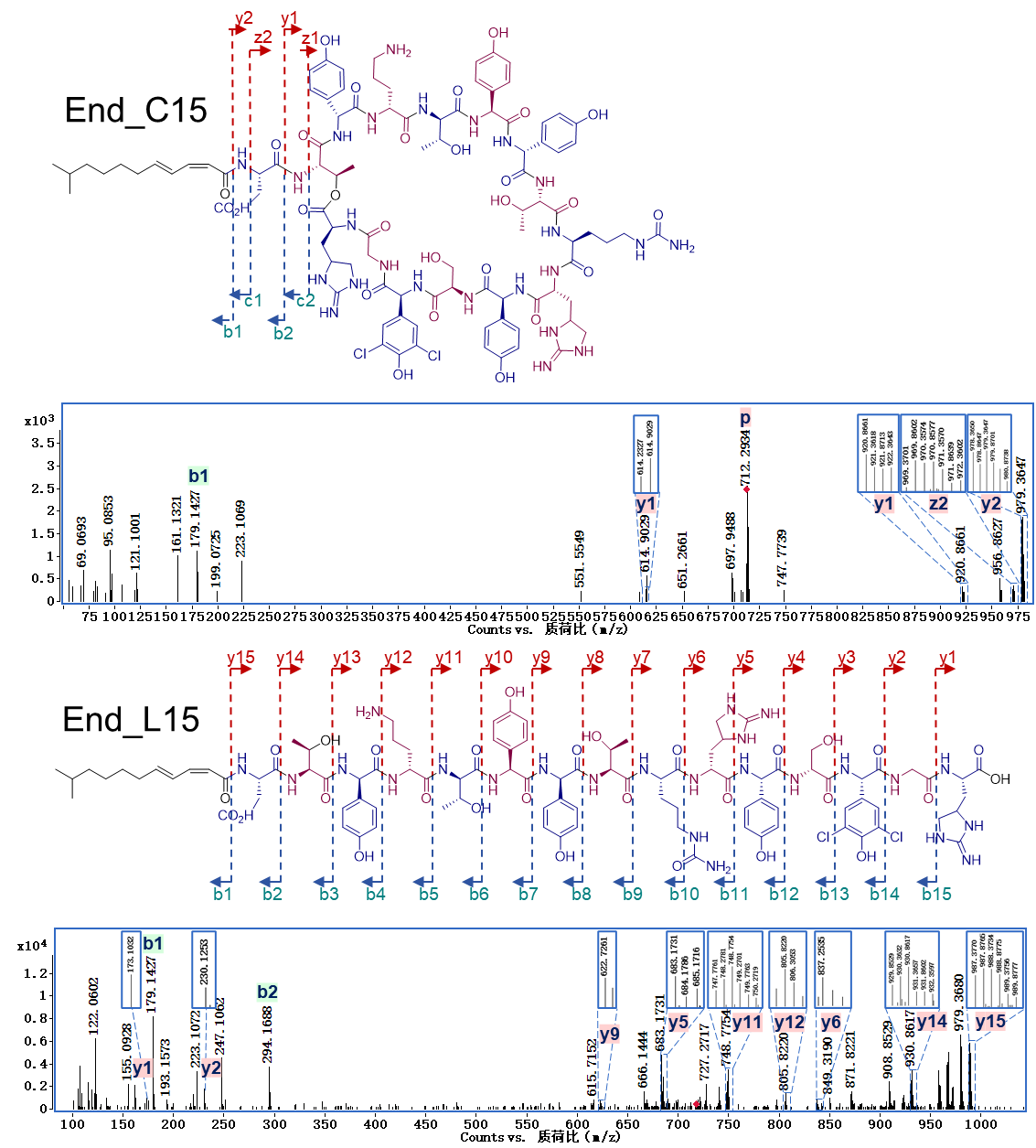


**Figure S30** LC–HRMS/MS spectra of End_C15 and End_L15.

Representative MS/MS fragmentation spectra of End_C15 and End_L15 with annotated fragment ions. The corresponding fragment formulas, theoretical *m/z*, observed *m/z*, and mass errors are summarized in Tables S19–S20.

End_C15 CID = 24.0 eV; prec. *m/z* 711.9605 [*z*=3]

End_L15 CID = 24.0 eV; prec. *m/z* 717.9650 [*z*=3]

**
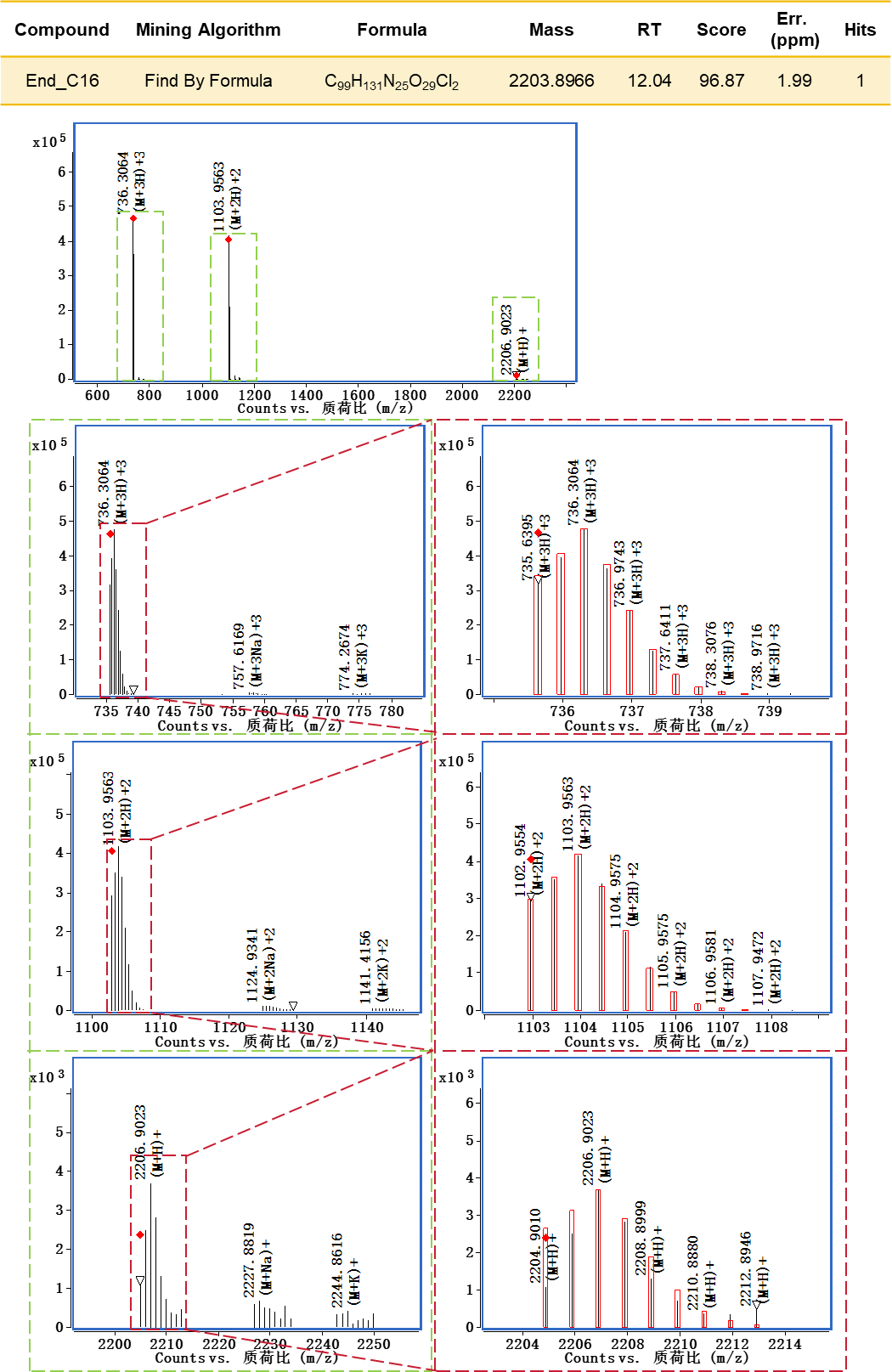
**

**Figure S31** LC–HRMS and isotopic distribution analysis of End_C16.

Detection of the target compound End_C16 by the Find-by-Formula algorithm, with representative MS and isotopic distribution analysis. Shown are (i) the full mass spectrum with annotated charge states, (ii) magnified views of selected charge states, and (iii) overlays of the observed and predicted isotopic distributions.


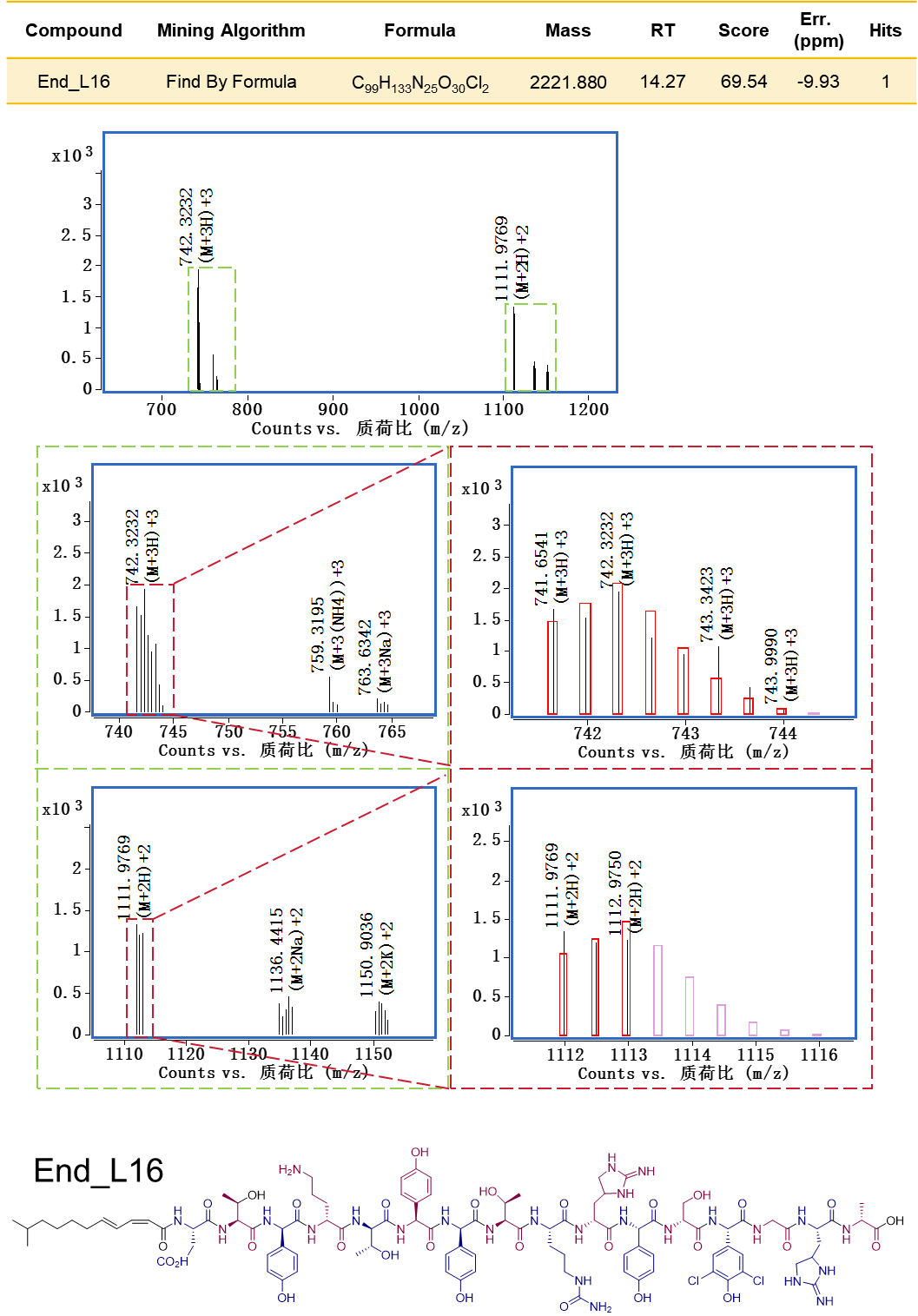


**Figure S32** LC–HRMS and isotopic distribution analysis of End_L16.

Detection of the target compound End_L16 by the Find-by-Formula algorithm, with representative MS and isotopic distribution analysis. Shown are (i) the full mass spectrum with annotated charge states, (ii) magnified views of selected charge states, and (iii) overlays of the observed and predicted isotopic distributions. Due to low signal intensity (~10^3^ counts), no MS/MS spectrum was obtained.


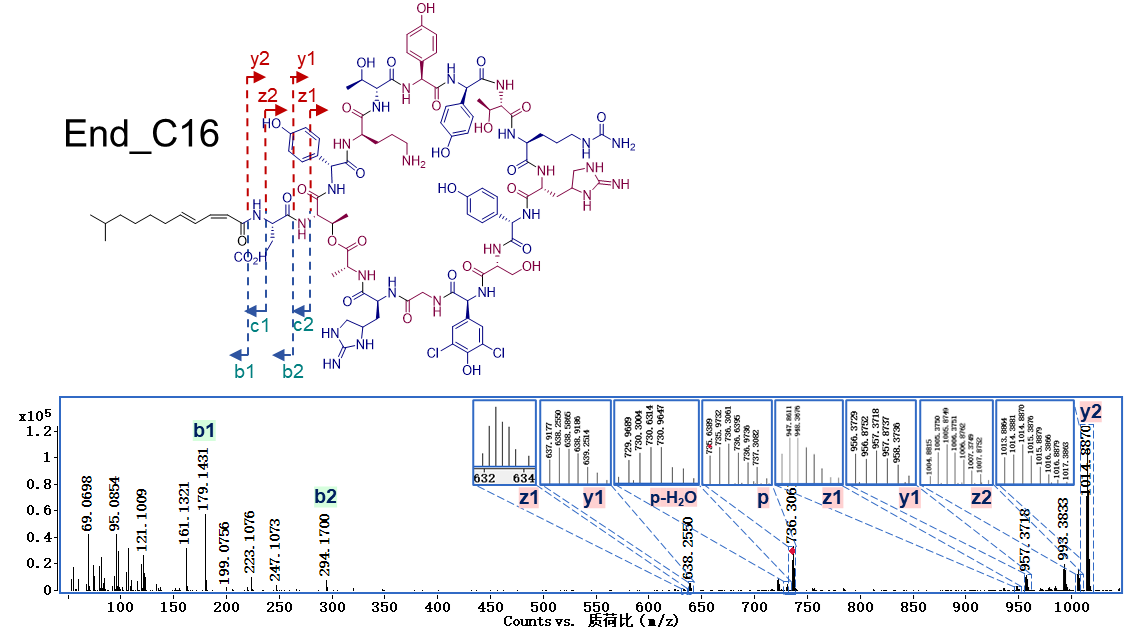


**Figure S33** LC–HRMS/MS spectrum of End_C16.

Representative MS/MS fragmentation spectrum of End_C16 with annotated fragment ions. The corresponding fragment formulas, theoretical *m/z*, observed *m/z*, and mass errors are summarized in Table S21.

End_C16 CID = 27.0 eV; prec. *m/z* 735.6389 [*z*=3]


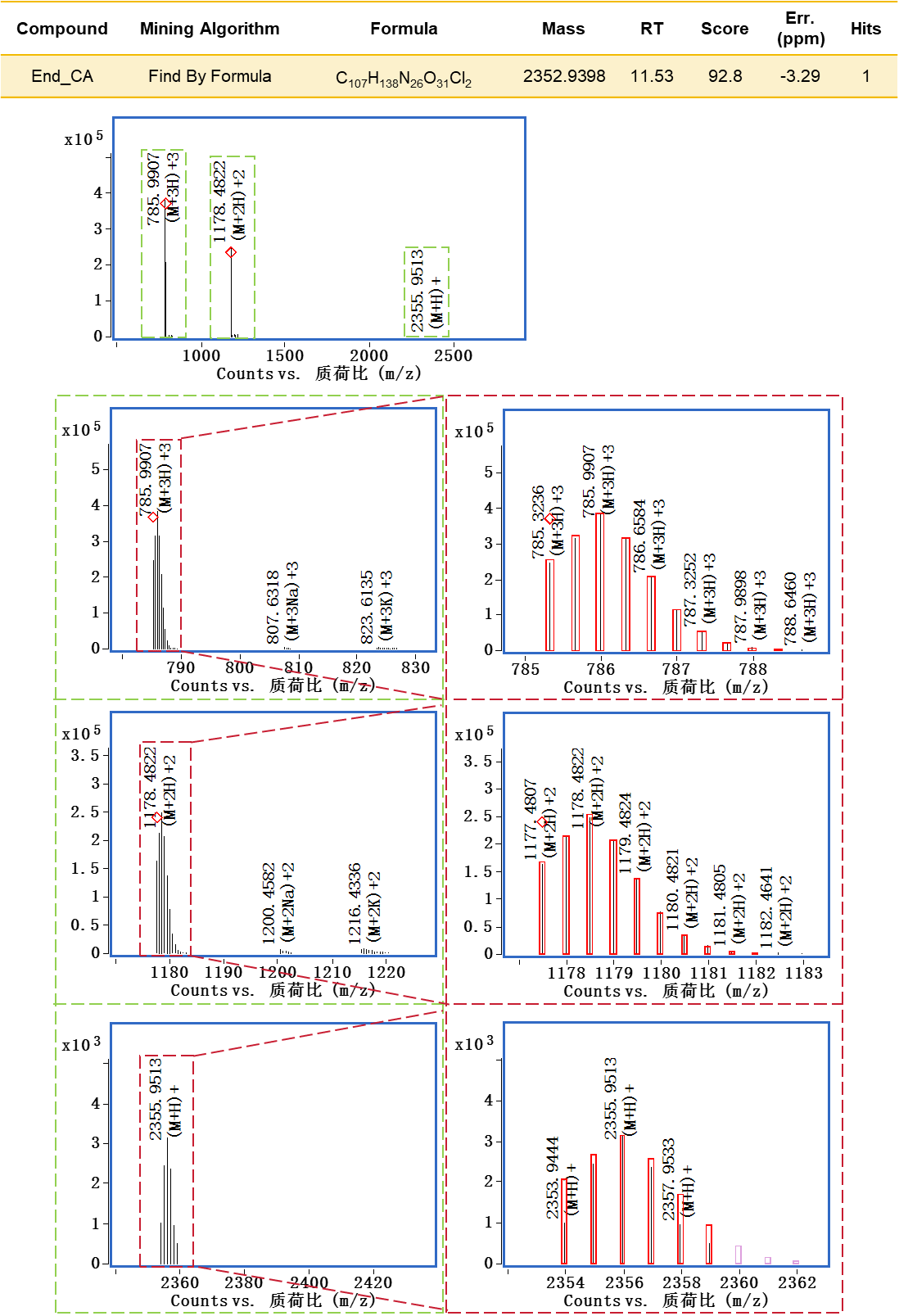


**Figure S34** LC–HRMS and isotopic distribution analysis of End_CA.

Detection of the target compound enduracidin A (End_CA) by the Find-by-Formula algorithm, with representative MS and isotopic distribution analysis. Shown are (i) the full mass spectrum with annotated charge states, (ii) magnified views of selected charge states, and (iii) overlays of the observed and predicted isotopic distributions.


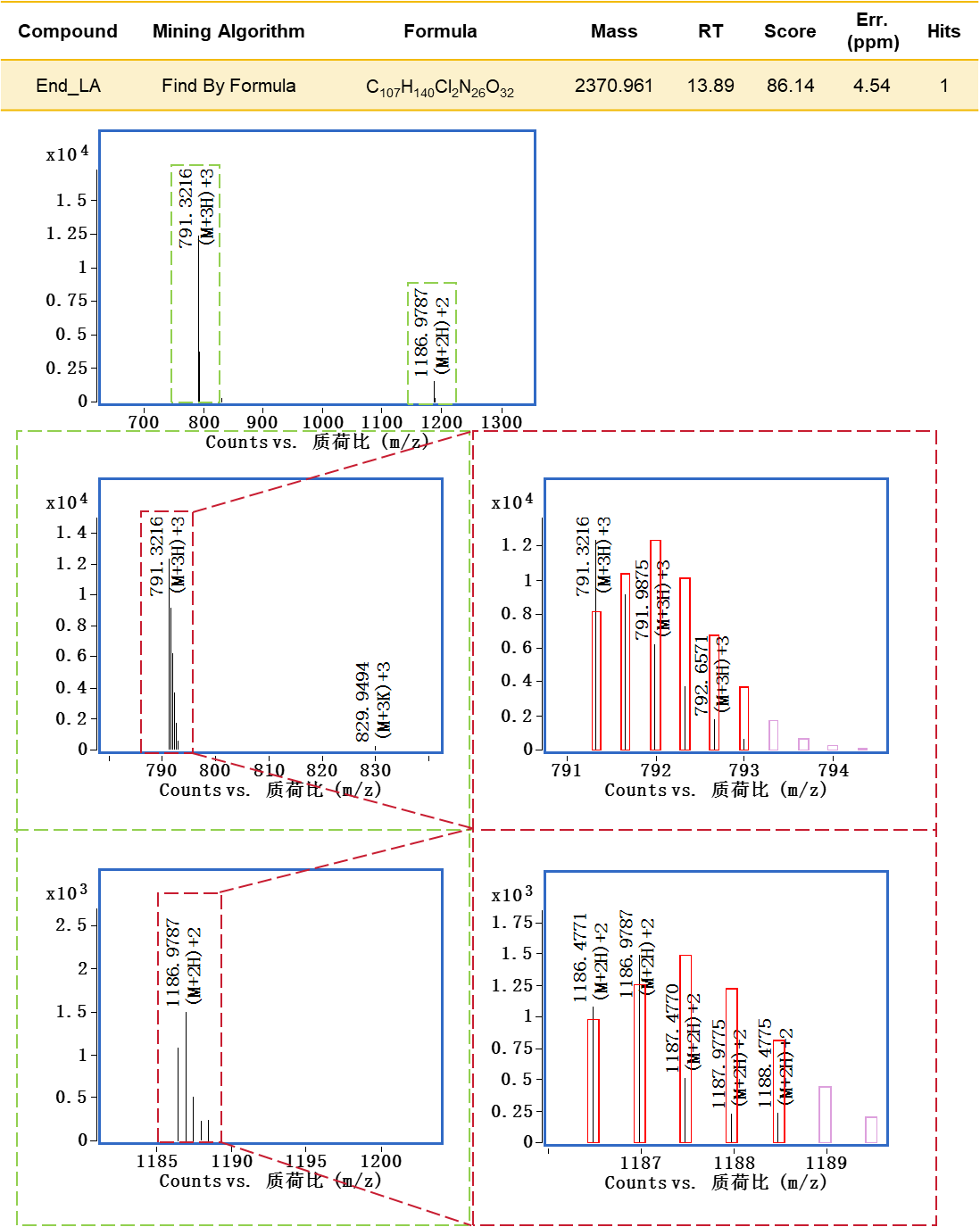


**Figure S35** LC–HRMS and isotopic distribution analysis of End_LA.

Detection of the target compound enduracidin linear A (End_LA) by the Find-by-Formula algorithm, with representative MS and isotopic distribution analysis. Shown are (i) the full mass spectrum with annotated charge states, (ii) magnified views of selected charge states, and (iii) overlays of the observed and predicted isotopic distributions.


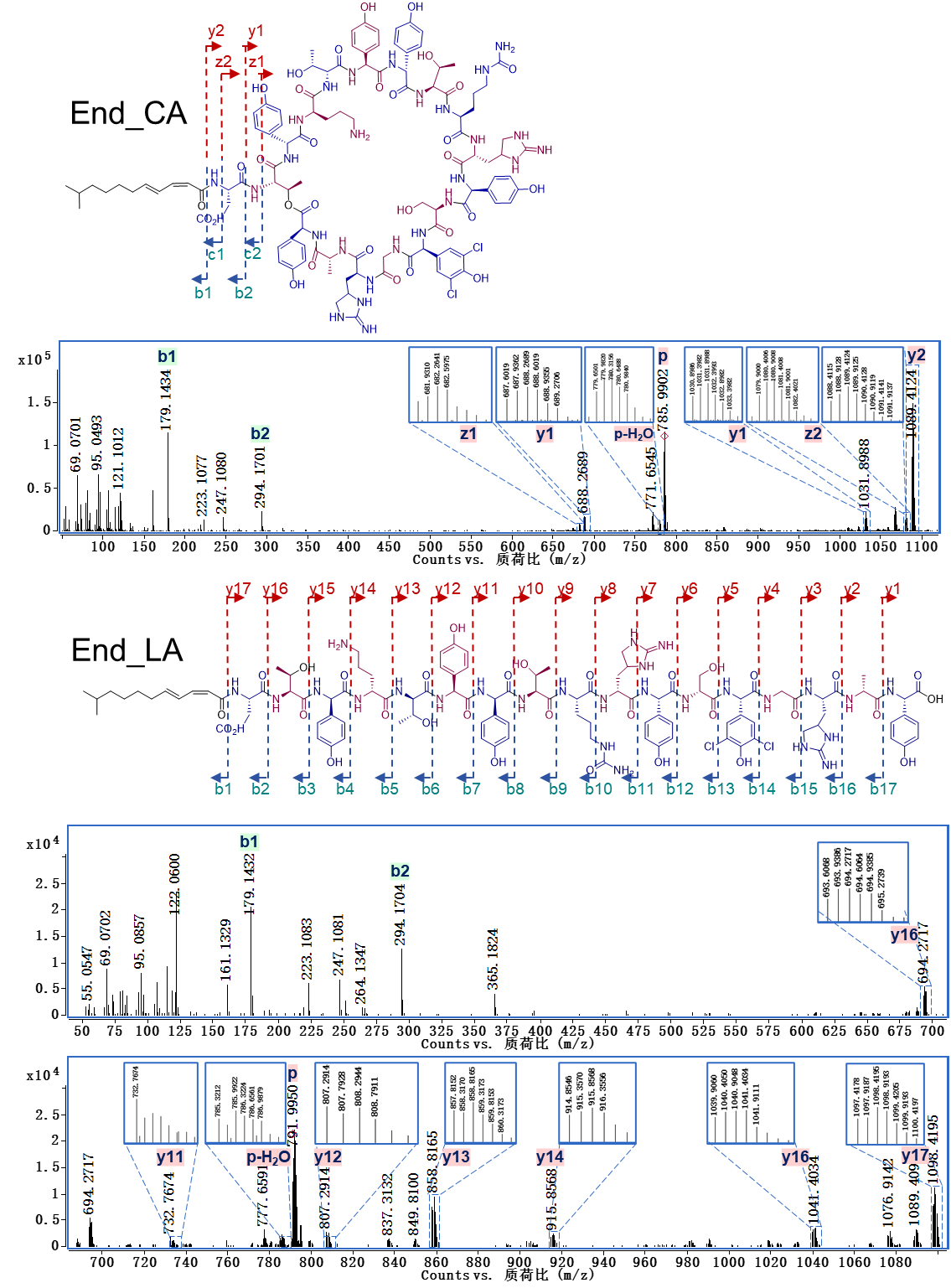


**Figure S36** LC–HRMS/MS spectra of End_CA and End_LA.

Representative MS/MS fragmentation spectra of End_CA and End_LA with annotated fragment ions. The corresponding fragment formulas, theoretical *m/z*, observed *m/z*, and mass errors are summarized in Tables S22–S23.

End_CA CID = 25.0 eV; prec. *m/z* 785.3229 [*z*=3]

End_LA CID = 25.0 eV; prec. *m/z* 791.3277 [*z*=3]


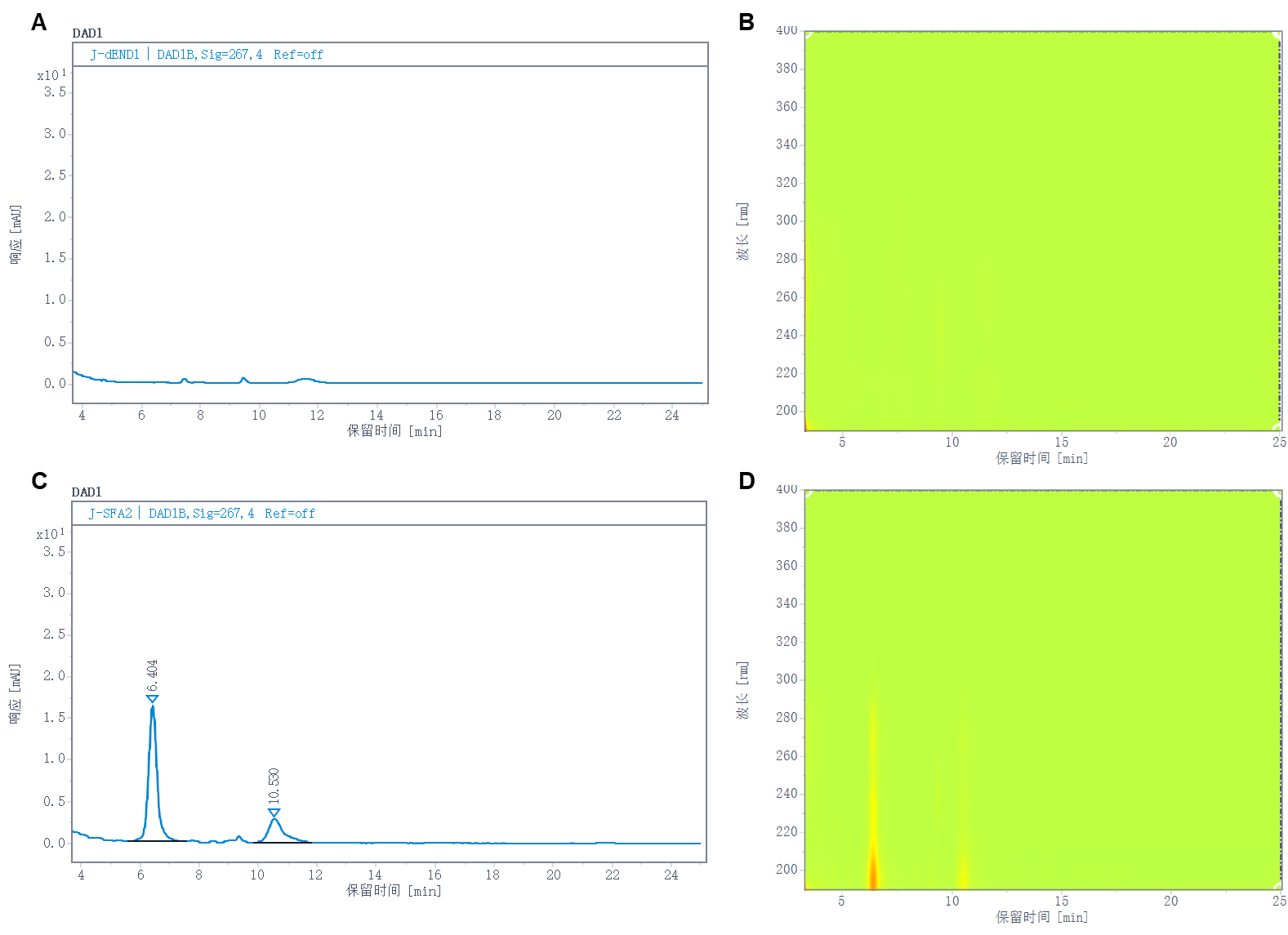


**Figure S37** HPLC–DAD analysis of the EndD (*CNQ36_25655*) in-frame deletion mutant and wild-type strain SFA.

(A) HPLC chromatogram of the ΔendD mutant extract monitored at 267 nm. No characteristic peaks corresponding to enduracidin A and B were observed at the expected retention times. (B) 2D UV absorbance contour plot of the ΔendD mutant extract (190–400 nm). No distinct UV-absorbing signals for enduracidin A, enduracidin B or other metabolites were detected across the entire detection range. (C) HPLC chromatogram of the wild-type strain SFA extract monitored at 267 nm. Two prominent peaks corresponding to enduracidin A (at 6.404 min) and enduracidin B (at 10.530 min) were observed at their expected retention times, respectively. (D) 2D UV absorbance contour plot of the wild-type strain SFA extract (190–400 nm). Distinct UV-absorbing spots corresponding to enduracidin A and B was detected at the expected retention times and wavelengths


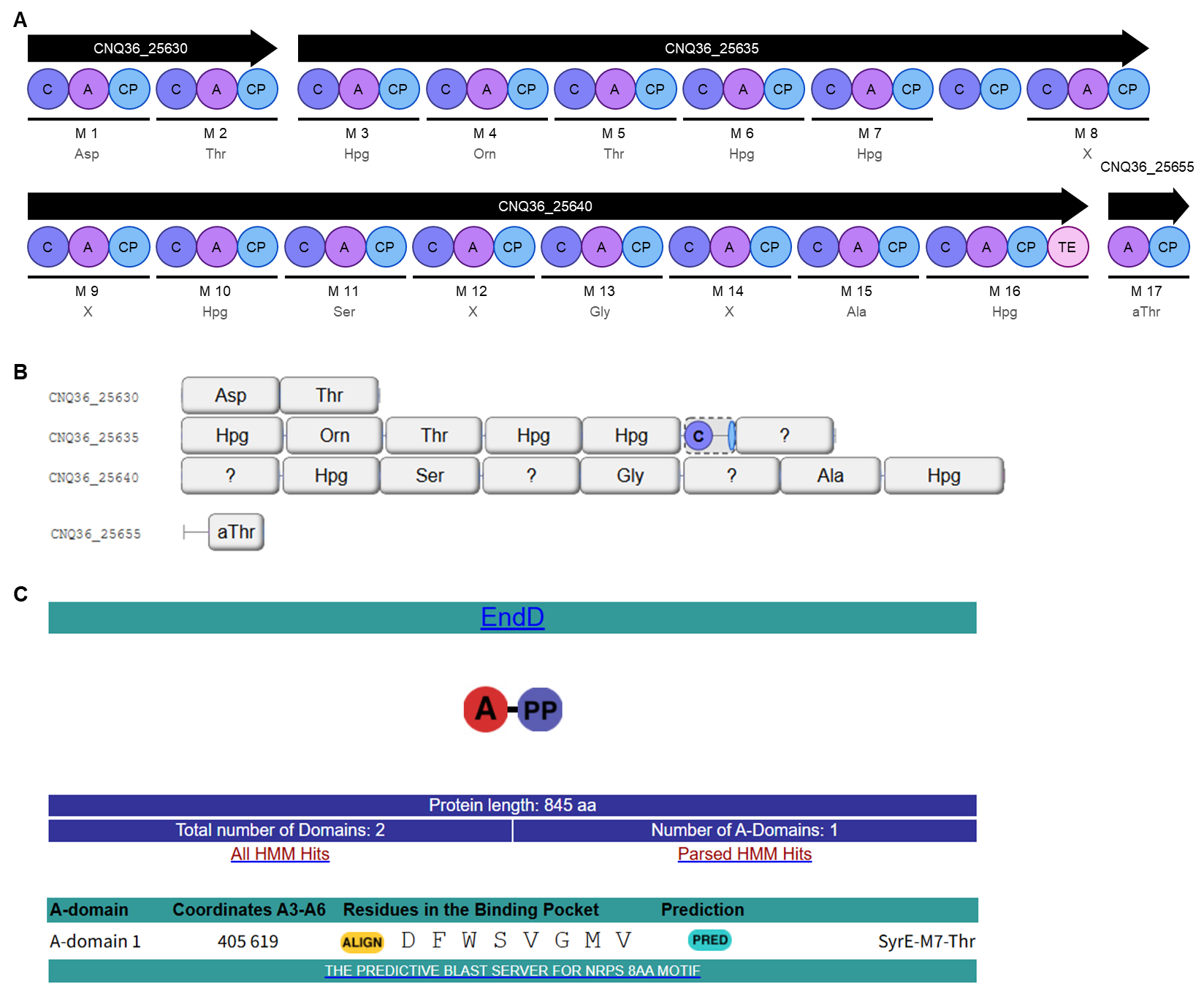


**Figure S38** Bioinformatic analysis of NRPS modules and domain organization in the enduracidin biosynthetic gene cluster.

(A) Module organization of the enduracidin NRPS assembly line predicted by antiSMASH 6.0.1 (NRPS/PKS modules view). The domain architectures of the four core NRPS genes (*CNQ36_25630*, *CNQ36_25635*, *CNQ36_25640*, and *CNQ36_25655*) are shown, illustrating the modular arrangement and domain composition across the assembly line.

(B) Detailed domain annotation and substrate specificity prediction generated by antiSMASH 6.0.1 (NRPS/PKS domains view). The predicted amino acid specificities of individual adenylation (A) domains within each module of the four NRPSs are indicated.

(C) Domain architecture of EndD predicted by the PKS/NRPS Analysis Web-site. EndD is predicted to be a single-module NRPS containing an A and a thiolation (T) domain. The A domain is predicted to recognize threonine, with the substrate-binding pocket residues identified as DFWSVGMV.


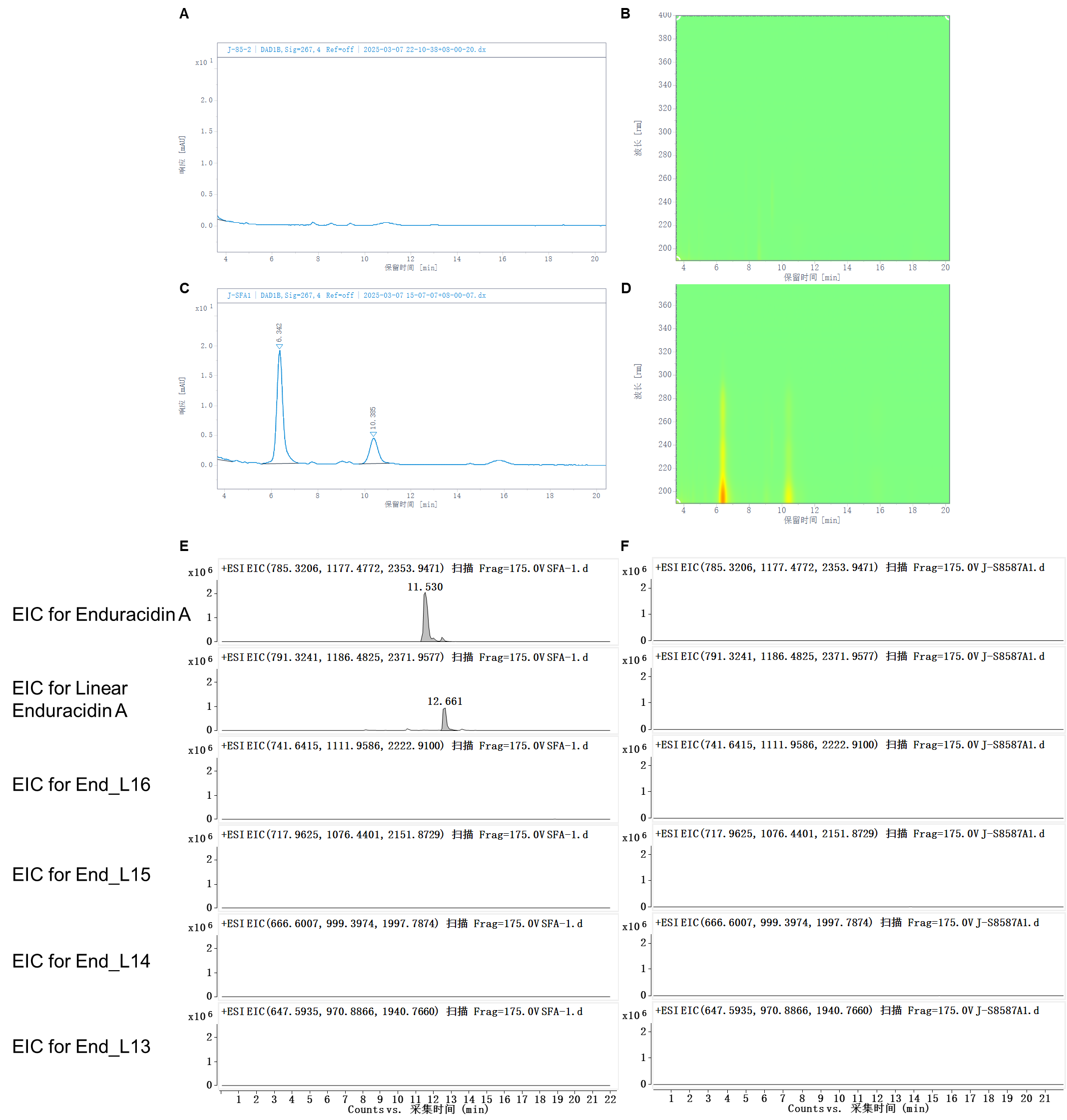


**Figure S39** HPLC–DAD and LC–HRMS analyses of the EndC_TE S8587A mutant and wild-type strain SFA.

(A) HPLC chromatogram of the S8587A mutant monitored at 267 nm. (B) 2D UV absorbance contour plot of the S8587A mutant (190–400 nm). (C) HPLC chromatogram of the wild-type strain SFA monitored at 267 nm. (D) 2D UV absorbance contour plot of the wild-type strain SFA (190–400 nm). (E) EICs for enduracidin A, linear enduracidin A, and truncated intermediates (End_L13 to End_L16) from the wild-type strain. (F) EICs for enduracidin A, linear enduracidin A, and truncated intermediates (End_L13 to End_L16) from the S8587A mutant.


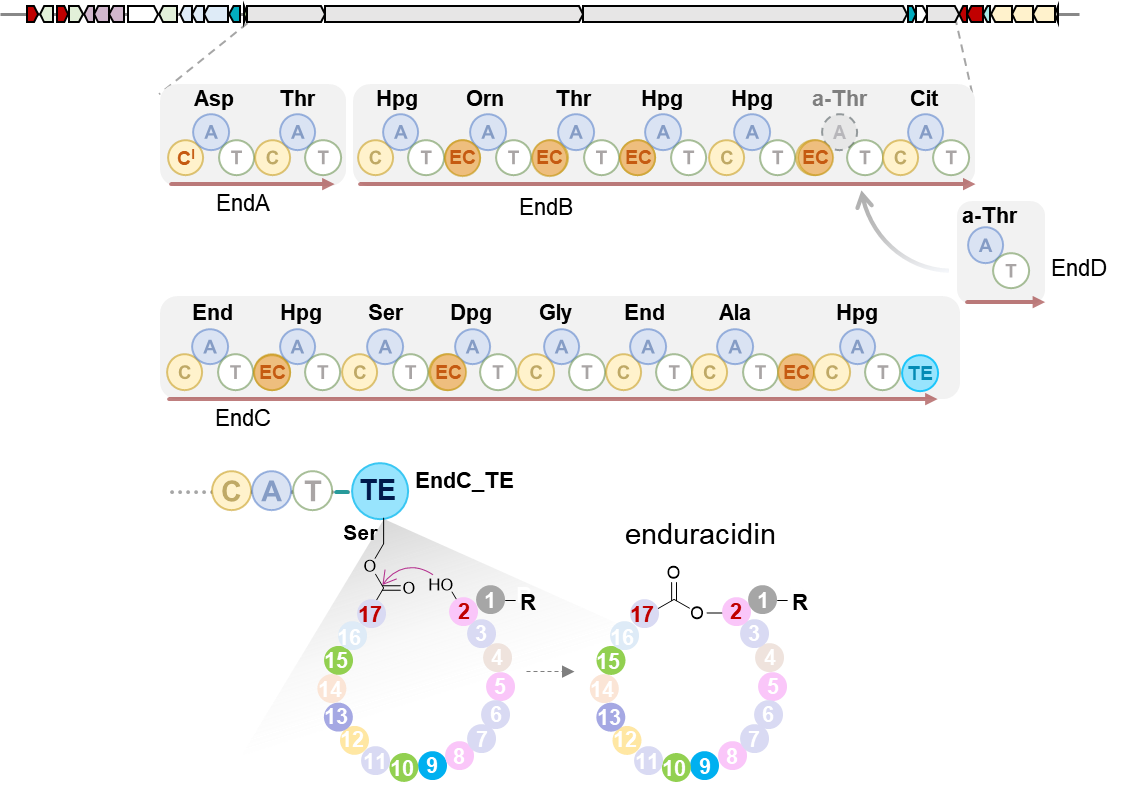


**Figure S40** Schematic illustration of the biosynthetic logic of enduracidin.

EndC_TE catalyzes macrocyclization through formation of an intramolecular ester bond between the hydroxyl group of the second residue (threonine, Thr) and the carboxylate of the seventeenth residue (hydroxyphenylglycine, Hpg). The letter R denotes the fatty acid chain of enduracidin.
